# A Rapid fMRI Paradigm for Localisation of the Language Network

**DOI:** 10.1111/ejn.70448

**Published:** 2026-03-06

**Authors:** Swati Jain, Emmanuel A. Stamatakis, Stephen J. Price

**Affiliations:** ^1^ Department of Clinical Neurosciences University of Cambridge Cambridge UK; ^2^ Division of Anaesthesia University of Cambridge Cambridge UK

**Keywords:** fMRI, language, rapid paradigms

## Abstract

Language assessment using imaging remains an arduous task due to the complexity of the language network. Given the complex neural instantiation of the language function, structural imaging is insufficient in determining the extensive network of relevant cortical and subcortical areas in patients with intrinsic brain tumours. Functional magnetic resonance imaging (fMRI) provides a non‐invasive method for the localisation of language‐dominant areas using task‐specific brain activation maps. In this study, the authors provide the results of a rapid four‐task‐based fMRI paradigm that can be used to determine an individual's specific language network. Fifteen participants were recruited prospectively for this study. A 10‐min pre‐scan preparation was done with each participant prior to the scan on the various tasks they were expected to complete during the fMRI scan. The following four tasks were conducted: covert naming, overt naming, sentence completion and pyramids and palm trees test (PPTT). The average time taken for all four tasks was 25.9 ± 1.8 min (23–31 min). All tasks were successfully completed in less than 30 min for all but one volunteer (31 min). Subject specific task‐based fMRI maps were generated for each task, providing overall insights into the bilaterally represented language network. Tasks were completed in a reasonable time duration, providing maximal information that can be translated into intraoperative testing during awake surgeries. Further research is needed to understand any limitations of this type of testing in patients with pre‐existing neurological deficits and brain lesions.

## Graphical abstract

This study describes a succinct four‐task fMRI paradigm to identify the language network, which can be translated to intraoperative tasks during direct electrical stimulation. The results show it is feasible within a reasonable time duration, providing maximal information of the accepted dual‐stream language network.

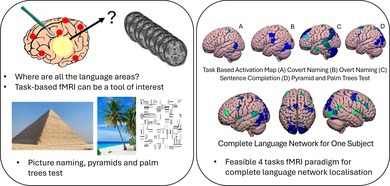

**Abbreviations**
BOLD: blood oxygen level dependent
EHQ: Edinburgh Handedness Questionnaire
fMRI: functional magnetic resonance imaging
GLM: General Linear Model
PPTT: Pyramids and Palm Trees Test
tSNR: temporal signal to noise ratio

## 1 Introduction

Awake craniotomy with intraoperative direct cortical stimulation is now considered the gold standard for patients with brain tumours affecting the eloquent areas of the brain. The more aggressive the resection, the greater the likelihood of progression‐free survival. This must be balanced against neurological deficits that can significantly impact their quality of life and subsequent treatment (Duffau and Mandonnet 2013; Duffau et al. 2022; Mandonnet and Duffau 2018). This is even more concerning when the tumour is in proximity or invading the cortical and subcortical language hubs of the brain. Intraoperative ‘language mapping’ is recommended to achieve the aim of maximal tumour resection while preventing any neurological deficits. To assess and preserve language function in these patients, a rigorous battery of language tests during the awake surgery is required (De Martino et al. 2021; Morshed et al. 2021).

Based on the techniques developed by Ojemann and Penfield (Ojemann 1983; Penfield and Boldrey 1937), stimulation of the regions of interest with short electrical impulses during the execution of a language task results in transient disruption of networks, which, if involved in language processing, results in the inability of the patient to complete the given task. These tasks (Chang et al. 2018; Collée et al. 2023; Morshed et al. 2021) can include picture naming, counting, word repetition, reading, writing and language syntax assessment. The type and duration of the task chosen are dependent on several factors (Mandonnet and Duffau 2018; Morshed et al. 2021; Pascual et al. 2024). This includes the location of the tumour and ‘expected’ areas of language affected, allowing the surgeon to choose a set of tasks that will give maximal information. Patient characteristics such as body habitus, pre‐existing neurological deficits and ability to be awake for a reasonable amount of time to avoid erroneous results leading to incomplete and inadequate tumour resection play a significant role as well. In the presence of a tumour in an eloquent area of the brain, if the patient is deemed unfit for awake surgery or is unable to complete the intraoperative tasks during the awake surgery, preoperative knowledge regarding the possible areas that are involved in language control becomes prudent and essential.

Language assessment using task‐based functional magnetic resonance imaging (fMRI) remains an arduous task. Anatomical structural imaging provides insight into the classical Lichtheim and Geschwind language model dependent on Broca's (Broadmann areas 44 and 45) and Wernicke's area (Broadmann area 22). Given the complexity of language area connectivity, structural imaging is insufficient in determining the involved cortical and subcortical areas. fMRI (Kokkinos et al. 2023; Stippich 2022) provides a non‐invasive method for localisation of language dominant areas using task‐specific brain activation maps. The maps are generated from changes in the blood oxygen level dependent (BOLD) signal related to changes in neural activity in response to a task. Language lateralisation using fMRI has been well studied and compared to preoperative gold standard test of intracarotid amobarbital test (‘Wada Testing’) showing high sensitivity (83.5%) and specificity (88.1%) (Htet et al. 2021). However, there are challenges in feasibly conducting tasks that assess various aspects of language function—syntax, semantics, phonological processing—in a reasonable time. These challenges include logistical issues, availability and training of personnel, and training patients to perform the tasks. Additionally, different neurosurgical centres have developed their own paradigms and protocols, making it difficult to replicate results or make comparisons.

Research into the neurobiology of language has led to the understanding of the language network and is widely discussed both in terms of production and comprehension. A dominant language model is that discussing the dual stream network (Hickok 2012; Hickok 2022)—a bilaterally represented organised ventral stream is responsible for mapping sound to meaning, and the left lateralised dorsal stream which maps the acoustic signal to the articulatory motor commands. The interactions between these two streams allow real life conversations to happen via production and semantic lexical processing (Kargar and Jalilian 2024; Nozari 2023). A visual or auditory input is processed by the language network which then leads to a motor output in the form of speech production. Intraoperative tasks during awake surgery are often focussed heavily on picture naming due to the generalised involvement of frontal, parietal and temporal lobes in visual processing and motor output. Picture naming tasks are easy to administer, and minimal preoperative training is required. Counting is often used for ease of administration and understanding speech arrest when areas involved are stimulated. Tasks such as writing and language syntax are often more complex to set up and thus their usage during the surgery is minimal.

The American Society of Functional Neuroradiology (Black et al. 2017) released a white paper in 2017 recommending sentence completion, silent word generation, rhyming, object naming, and/or passive story listening. While these tasks are meant to provide adequate information for preoperative language assessment, this kind of protocol requires significant preparation and personnel in administration. For a successful preoperative fMRI to be accomplished in patients, it should be able to fulfil the following requirements: (1) pretest and preoperative training that can be performed in a reasonable time, (2) tasks during fMRI that can be seamlessly transferred to intraoperative awake testing to allow comparisons, (3) completion of all standard preoperative sequences along with task‐based fMRI within a reasonable time to avoid patient fatigue in the scanner, (4) contingency plans for patients who suffer from neurological deficits that are unable to complete the proposed tasks in the scanner and (5) results of the fMRI should be reliable and can replace/augment information obtained during awake craniotomies. The authors here queried if a succinct set of tasks can provide maximal information of the language network across its various domain fulfilling the above objectives.

To fulfil the aims the authors evaluated the available paradigms that would activate both dorsal and ventral streams, and classical Broca's and Wernicke's area. The tasks should also provide information with regard to language lateralisation to allow the surgeon to have insights with regard to hemispheric dominance. The following task paradigms were chosen: covert naming, overt naming, sentence completion, and pyramids and palm trees test given the current recommendations for preoperative assessment of language paradigms.

In this study, the authors propose and evaluate a rapid four‐task‐based fMRI paradigm to provide maximal information on the individual's language network. The authors assess the feasibility of completing the proposed tasks within a reasonable time duration along with the standard MRI structural sequences.

## 2 Materials and Methods

### 2.1 Participant Recruitment and Demographics

Fifteen participants were recruited prospectively for this study. This study was approved by the institutional review board and all participants had provided their consent to participate in the study. A study invite was sent via email to the university staff and students for recruitment. The inclusion criteria were as follows: participants should be more than 18 years of age, have had no previous brain surgery, no contraindications to undergoing MRI scanning, subjectively reported themselves as right‐handed and have English as their first spoken and written language. The main exclusion criteria were any contraindications to undergoing MRI, claustrophobia or any other reasons requiring sedation during the scan, previous brain surgery and pre‐existing neurological deficits.

After informed consent, each participant was advised on the sequence of the various scans to be carried out. A 10‐min pre‐scan preparation was done with each participant prior to the scan on the various tasks they were expected to complete during the task‐based fMRI. For each of the tasks, a single random example from the stimuli was used to explain what the participant can expect during the scan. Demographic data including age, handedness (using Edinburgh Handedness Questionnaire [EHQ]), education level, primary and secondary language (if present) were recorded.

### 2.2 fMRI Protocol

All scans were performed at the Wolfson Brain Imaging Centre using the Siemens 3T Prisma*fit* with 80 mT/m gradient coils.

#### 2.2.1 Anatomical Images

T1 weighted images were acquired with a voxel size of 1.0 × 1.0 × 1.0 mm, TR 2300 ms, TE 2.98 ms. No contrast was administered for volunteering participants. Authors are mindful that scanning patients will require contrast enhanced imaging as part of the preoperative work up.

#### 2.2.2 Functional Images

**FIGURE 1:**
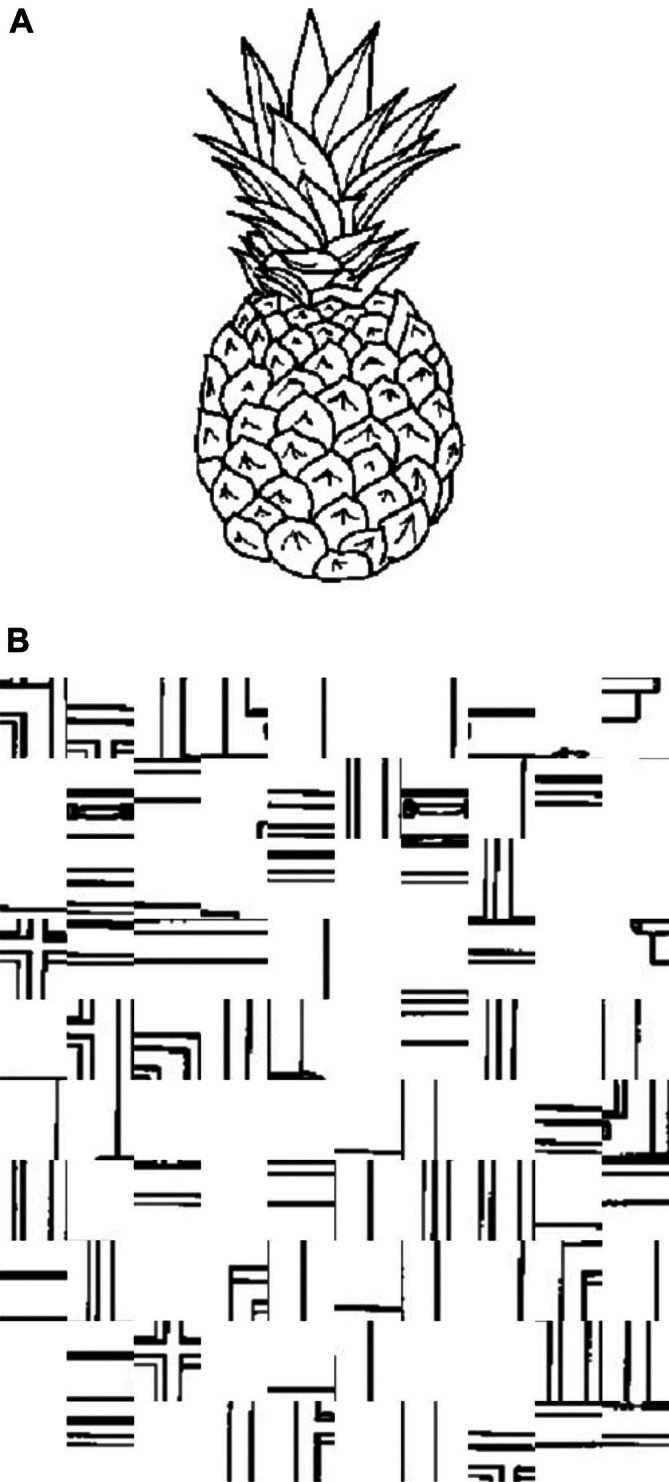
(A) Example of stimulus shown during event phase of covert naming. (B) Example of stimulus shown during control phase of covert naming.

**FIGURE 2:**
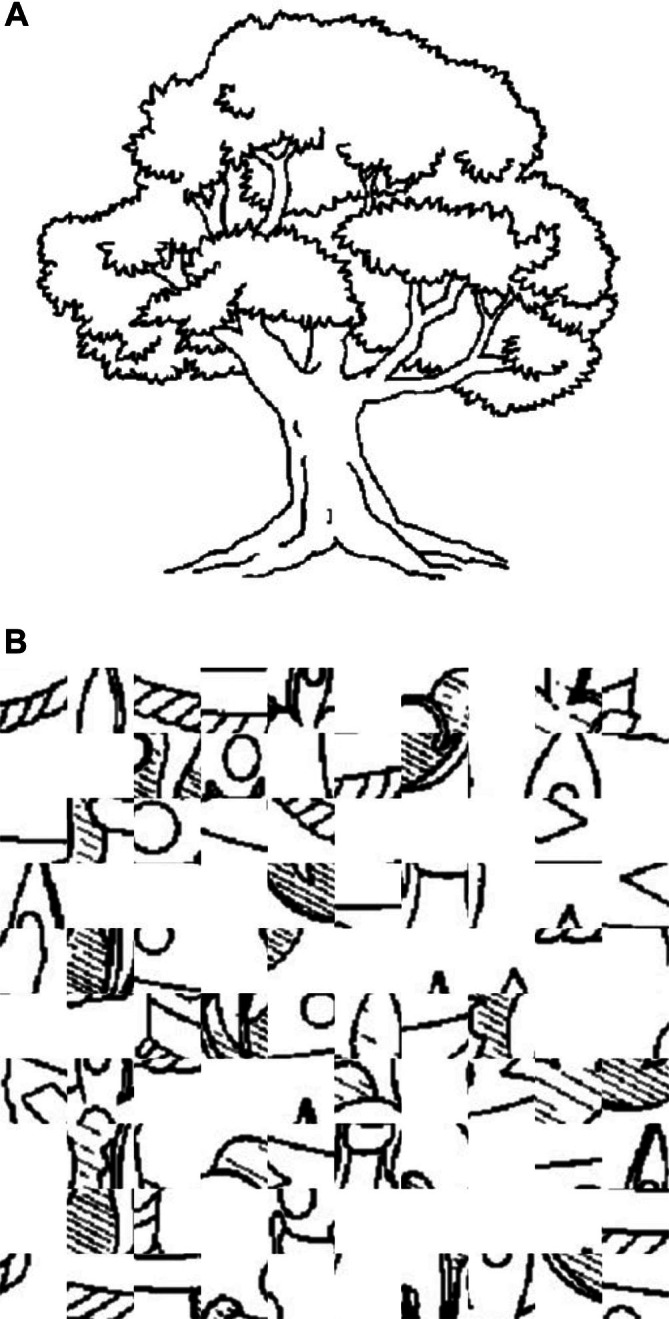
(A) Example of stimulus shown during event phase of overt naming. (B) Example of stimulus shown during control phase of overt naming.

**FIGURE 3:**
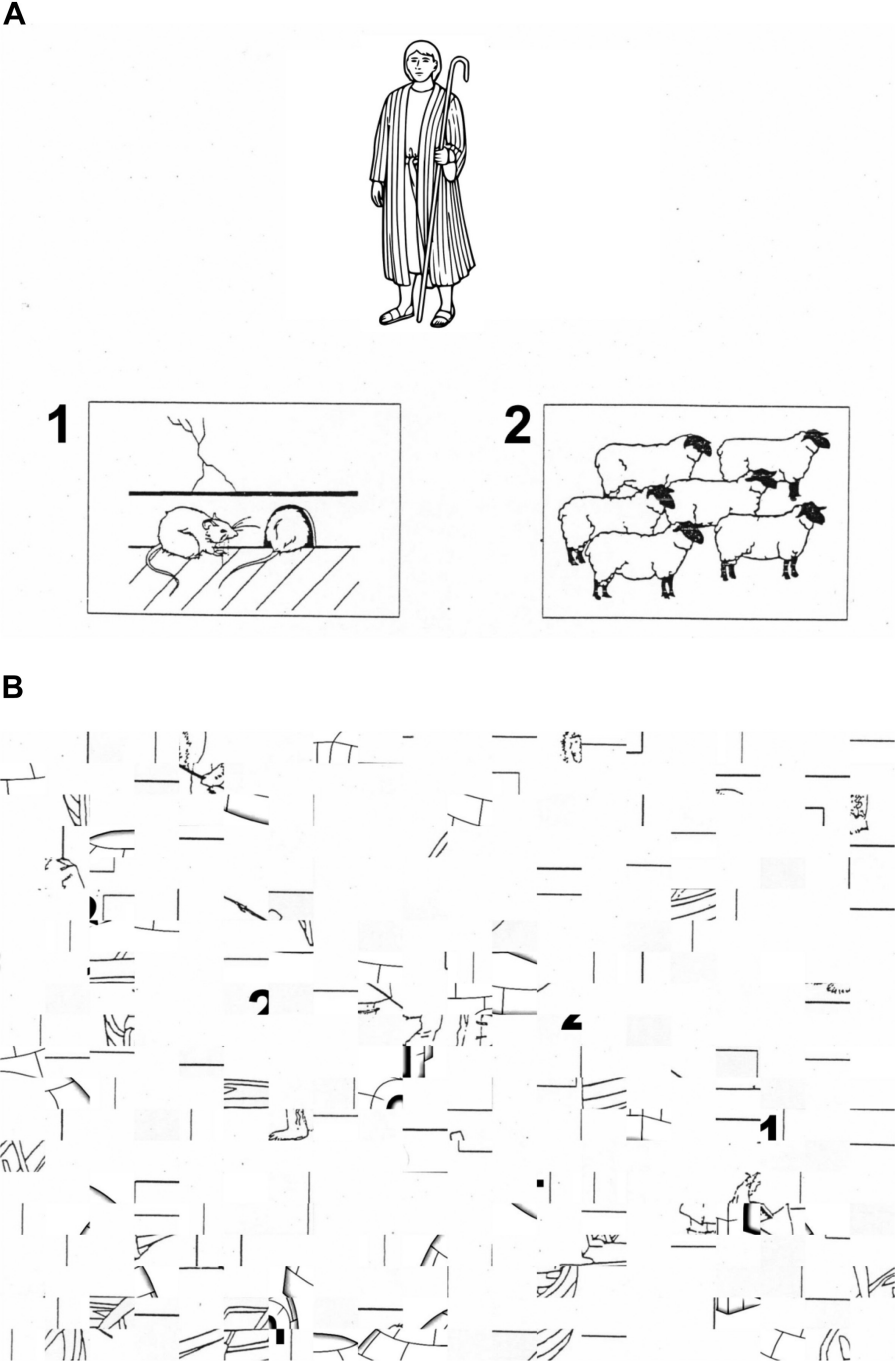
(A) Example of stimulus shown during event phase of pyramid and palm trees test. (B) Example of stimulus shown during control phase of pyramid and palm trees test.

The following functional images were acquired:

1. Resting state fMRI: Participants were advised to keep their eyes closed and think about ‘nothing in particular’. The lights in the scanner were dimmed to minimise visual stimulation during the acquisition of the images. The images were acquired over 8 min with a voxel size of 3.0 × 3.0 × 3.0 mm, TR 2600 ms and TE 28.0 ms.
2. Task‐based fMRI: After completion of anatomical and resting state fMRI, the participants were asked to perform four tasks. All images were acquired with a voxel size of 3.0 × 3.0 × 3.0 mm, TR 2000 ms and TE 30.0 ms. The flip angle was 60°, and field of view (FOV) resolution was 240 mm. 36 slices were acquired with a slice thickness of 3 mm. Jitter was added to each event and control to allow sampling along all time‐points of the haemodynamic response function (HRF) curve. For all tasks, an average jitter of 0.25 s was added with a range between 0 to 0.5 s.
  - Covert Object Naming (Silent Picture Naming, Figure 1) (Black et al. 2017; Hirsch et al. 2000; Shmuelof and Zohary 2005; Trimmel et al. 2019): Covert object naming is an expressive language paradigm in which the subject is shown an object and asked to silently name the presented object. The control task in this paradigm can be either a scrambled picture or a blank screen. This task is expected to yield activation in the inferior frontal gyrus, middle frontal gyrus, ventral occipitotemporal cortex and posterior temporoparietal cortex. This task is very useful in patients who may suffer from aphasia or dysphasia but are still able to recognise objects, is easy to administer and can be performed in any language. This task does not assess speech articulation or the motor cortex involved in speech production. Participant engagement cannot be assessed during this task as no assessable output is generated.
    No verbal or motor input was required from the participant. An event‐control‐resting state sequence was used. During the event (Figure 1), the participant is shown an object for 3 s. They are asked to mentally verbalise the name of the object shown without any verbal output. During the control condition (Figure 1B), they are shown a scrambled image of a different object for 3 s. During the fixation cross block, a black cross on a white background is shown for 5 s.
  - Overt Object Naming (Loud Picture Naming, Figure 2) (Barch et al. 1999; Htet et al. 2021; Shmuelof and Zohary 2005): Overt object naming in an expressive language paradigm focusing on speech product in which the subject is shown an object and asked to loudly name the presented object so that the person administering the test can hear. The control task in this paradigm can be either a scrambled picture or a blank screen. This task focuses on the cortical motor pathway for speech production. Patients who have dysphasia or aphasia in the setting of neurological conditions or tumours often are unable to articulate well despite being able to think about the name of the object during the covert naming task (Martin et al. 2005). Without audible responses as in covert naming, it would not be possible to tell if the patient has had any naming errors, which is essential to understanding the localisation during direct cortical stimulation intraoperatively. As tumours may involve both language processing and articulation, it is prudent to test overt object naming. Problems with head movements during overt object naming can be tackled by restricting movement of the jaw, and this has been discussed further in materials and methods. The authors postulate that overt object naming will provide further insights into the language network beyond the advantages of covert object naming.
    Participants were advised to say the name of the object. An event‐control‐resting state sequence was used. During the event (Figure 2), they were shown an object for 3 s. They were asked to verbalise the name of the object shown. During the control condition (Figure 2B), participants were shown a scrambled image of a different object for 3 s. During the fixation cross block, a black cross on a white background was shown for 5 s. This series was repeated for 30 times. To avoid excessive head movements during this task, participants were advised to avoid any significant jaw and mouth movements to prevent movement artefacts. A Durapore medical tape was placed along the jaw to the head shield to further prevent excessive movements during the task (Krause et al. 2019). A set of 30 stimuli used in the overt task was the same set of images used in the covert task. However, they were presented in a pseudo‐randomised order to avoid repetitions for both covert and overt naming.
  - Silent Sentence Completion (Table 1) (Ashtari et al. 2005; Black et al. 2017; Salek et al. 2017; Zacà et al. 2013): Silent sentence completion involves visual display of incomplete simple sentences with the subject asked to read the sentence silently and complete it without verbalising it. The control block involves gibberish or jumbled up words that are displayed in the same format. The sentence completion task is a semantic language paradigm that is involved in activating the superior temporal gyrus (Wernicke's territory) and inferior frontal gyrus (Broca's area) in the dominant hemisphere. The sentence completion task, when employed intraoperatively, facilitates the evaluation of both reading comprehension and syntactic abilities. It has been shown across multiple studies to have more robust activation when compared to a verbal fluency task (Salek et al. 2017; Zacà et al. 2013). Having a robust paradigm that assesses comprehension is prudent in understanding the language network distribution across hemispheres.
    This task is completed in event‐control format. The participant is shown four incomplete sentences and asked to complete the sentence mentally in as many iterations as possible prior to the next sentence being shown. They are then shown a series of four gibberish scrambled incomplete sentences as control. This cycle is repeated 4 times with each event and control lasting for 20 s each.
  - Pyramids and Palm Trees Test (PPTT) (Figure 3) (Duffau et al. 2005; Mehri et al. 2018; Pulvermüller 2013): PPTT was designed by Howard and Patterson in 1992 as a means of non‐verbal semantic memory test to assess cognition in brain disorders, semantic dementia, Alzheimer's disease and aphasia. As semantic processing can be affected at both cortical and subcortical levels in brain tumours involving frontal, temporal, and insula, it is prudent that semantic processing is studied beyond what is understood from silent sentence completion. Patients with aphasia and dysphasia may still have intact semantic processing. Without preoperative or intraoperative assessment of semantic processing, it may be inadvertently damaged during tumour resection. The authors decided to use this test to understand the underlying semantic functional network. During an event (Figure 3), the participant is shown three pictures arranged in the form of a triangle, with pictures labelled as 1 and 2 at the inferior border of the triangle. They are asked to choose 1 or 2 on the button box depending on which picture they think is closest to the picture at the vertex of the triangle. Once the button box is pressed, the screen moves to a control event (Figure 3B) where scrambled pictures are shown. The participant is asked to press 1 or 2 on the button box. This is followed by a fixation cross block; a black cross on a white background is shown for 5 s. This series would be repeated for 30 times.
    The task for each paradigm was displayed using a script running on the Pyschtoolbox code via MATLAB 2023a (https://www.mathworks.co.uk/products/matlab/; Mathworks, Sherborn, MA, USA). The MATLAB script allowed capture of timings of task displays and any participant input that was required during the PPTT task. The imaging protocol also included the acquisition of diffusion tractography imaging. However, these images were not analysed for this study.

**TABLE 1:** Sentences used in sentence completion task.

‘I feel happiest when…’,
‘My biggest fear is…’,
‘In a stressful situation, I usually…’,
‘I wish I could go to…’,
‘If I see a lion, I would…’,
‘When I am angry, I…’,
‘My favourite colour is…’,
‘The cat chased the…’,
‘Something that makes me unique is…’,
‘The colour of my eyes is …’,
‘When the rat saw the dog, it…’,
‘My favourite TV show is…’,
‘The elephant is…’,
‘If I could go on a holiday, I would…’,
‘Last night, my dinner was…’,
‘Between tea and coffee, I…’,
‘I wake up at …’,
‘On weekends, I usually…’,
‘My favourite place in this city is …’,
‘Kangaroos are usually found in…’,
‘If I exercise, I usually…’,
‘My family consists of…’,
‘The last holiday I had, I…’,
‘My favourite animal is…’

### 2.3 fMRI Data Pre‐Processing

Statistical Parametric Mapping (SPM) software, Version 12 (Wellcome Department of Cognitive Neurology, implemented in MATLAB) was used for preprocessing and analysis of fMRI data. The images were preprocessed using slice timing correction, within participant re‐alignment, i.e., movement correction, structural scan co‐registration to the mean fMRI‐EPI image, co‐registered structural image segmentation and derivation of spatially normalised images into MNI space. Functional images were smoothed using an isotropic 6 mm full‐width half‐maximal (FWHM) Gaussian kernel.

### 2.4 fMRI Data Analysis and Statistical Modelling

Preprocessed functional images for each participant were entered within a first‐level general linear model (GLM) framework. The GLM model was generated for each paradigm by modelling the trial stimulus onset times, time to next event in the paradigm or response times in Paradigm 4 (PPTT) and HRF. A high pass filter with a period of 128 s was used to remove low‐frequency scanner noise. A design matrix was generated for all four tasks. To understand areas of activation involved in a task, a subtractive analysis was carried out between the event and the control block for each of the tasks. Resulting contrast images were entered into a second level group analysis to obtain within group activations for each task described earlier. For each participant, cluster defining voxel threshold to identify potential clusters for activation was set at *p* < 0.001. Cluster size threshold was defined using False Discovery Rate (FDR) at *p* < 0.05.

### 2.5 Laterality Index

To calculate the laterality index (LI) (Rolinski et al. 2020; Wegrzyn et al. 2019), right and left hemispheric masks of the cerebrum were generated. These masks were applied to extract the activation clusters. The LI was calculated using the following formula:

$$\mathrm{LI}=\frac{Q_{L}-Q_{R}}{Q_{L}+Q_{R}}$$

where Q_LH_ and Q_RH_ are representative numbers of voxels above the thresholds measured by fMRI for the left and right hemisphere contributions respectively. LH can range from −1 to +1, with values closer to −1 referring to a right hemispheric dominance and those to +1 to a left hemispheric dominance.

Additional statistical analyses were carried out on R (R Core Team 2024).

## 3 Results

Fifteen healthy volunteers with no previous medical and surgical history were included in this study. Table 2 provides detailed demographics of these volunteers. There were 6 females and 9 males with a mean age of 28.2 ± 5.6 years. All volunteers were right‐handed, with their 1st language of proficiency being English. Nine volunteers were bilingual, where they were able to speak, read, and write in the 2nd reported language. All tests were conducted in English as described in the materials and methods section earlier. Only 10 min were allocated to preparation time during which the participant interacted with the person administering the experiment prior to the acquisition of the scan. No extra time was allocated for preparation.

**TABLE 2:** Baseline demographics of the participants.

Subject	Age	Sex	Primary language	Secondary language (if any)	Handedness	Education level
1	27	F	English	Italian	Right	Post graduate
2	32	M	English	Thai	Right	Post graduate
3	22	M	English	—	Right	Post graduate
4	23	M	English	—	Right	Post graduate
5	32	M	English	Urdu	Right	Post graduate
6	24	F	English		Right	Post graduate
7	43	M	English	Chinese	Right	Post graduate
8	28	M	English	Tamil	Right	Post graduate
9	23	M	English	—	Right	Post graduate
10	29	F	English	—	Right	Post graduate
11	26	M	English	Spanish	Right	Post graduate
12	26	F	English	Arabic	Right	Graduate
13	23	M	English	—	Right	Post graduate
14	35	F	English	Ukrainian	Right	Post graduate
15	30	F	English	Bulgarian	Right	Post graduate

### 3.1 Duration of Scans and Task‐Based Paradigms

The start and end time of the scan were recorded for each participant. The average total duration for the entire MRI sequences (T1‐weighted anatomical scan, four‐task‐based fMRI paradigms, resting state fMRI and diffusion tensor imaging) was 61.8 ± 5.7 min, with maximal time taken by a participant being 80 min due to issues with the running of the task paradigm. To understand the time taken by each participant for each task including the setting up for paradigms, the time from the start of acquisition of the images for each task to the start of the next paradigm was recorded. Table 3 provides the average time taken for each task. The average time taken for all four tasks was 25.9 ± 1.8 min (23–31 min). All tasks were successfully completed in less than 30 min for all but one volunteer (31 min).

**TABLE 3:** Time taken to complete all tasks.

	Time (minutes)
1st Task	7.2 ± 0.9
2nd Task	6.5 ± 0.5
3rd Task	5.9 ± 1.0
4th Task	6.2 ± 1.1
Total duration of all tasks	25.9 ± 1.8

### 3.2 Language Network Localisation

#### 3.2.1 Task‐Based Localisation

All task‐based fMRI images were analysed using SPM as described in materials and methods. Distinct maps were generated for all subjects for each task. For covert naming, overt naming and PPTT tasks, a contrast was formed by assigning 1 and −1 weights to event and control block respectively. No contrast was assigned to the fixation cross. For sentence completion task, 1 and −1 were assigned to the event and control block respectively.

**FIGURE 4:**
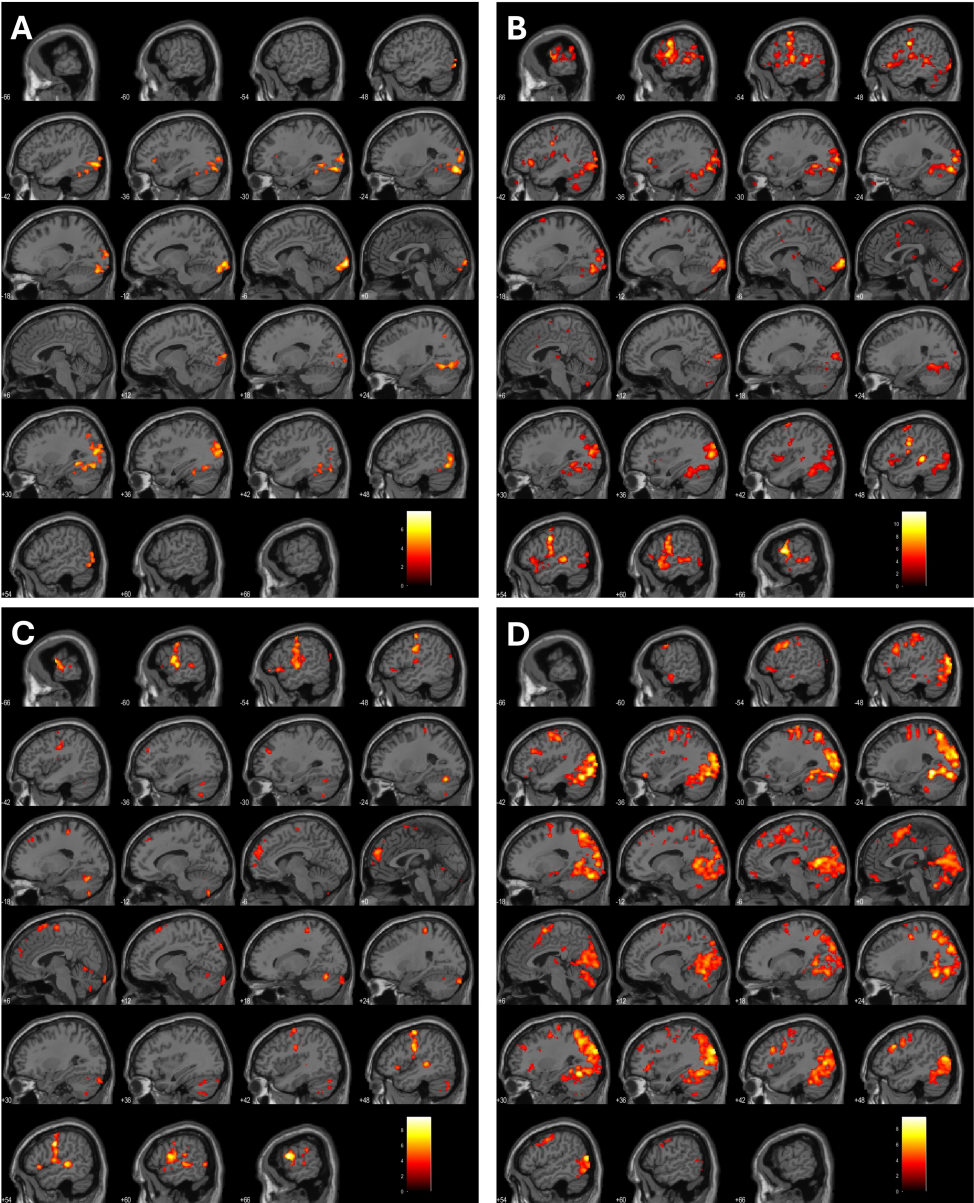
(A–D) activation areas for all four tasks for Subjects 8.

**FIGURE 5:**
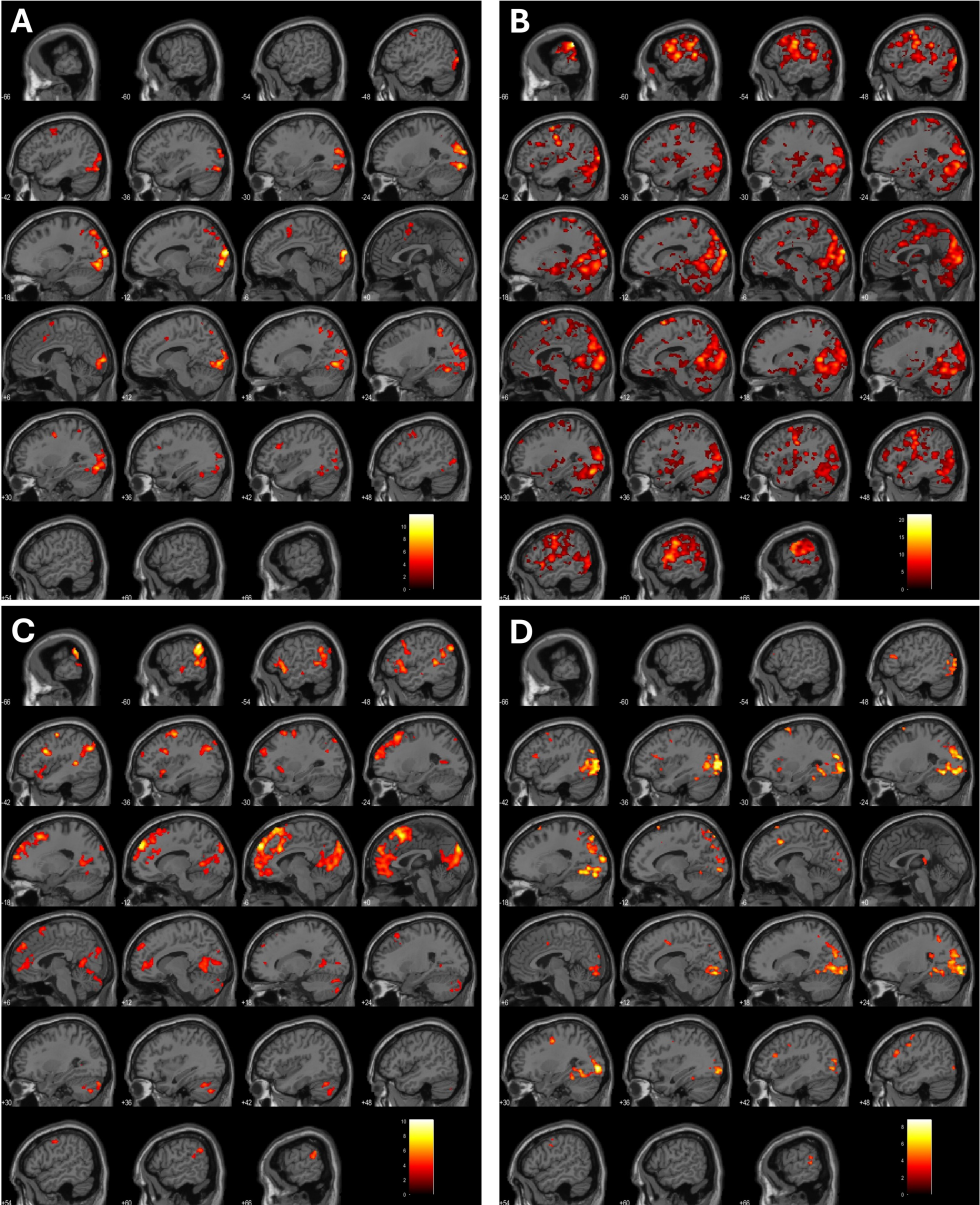
(A–D) activation areas for all four tasks for Subjects 10.

**FIGURE 6:**
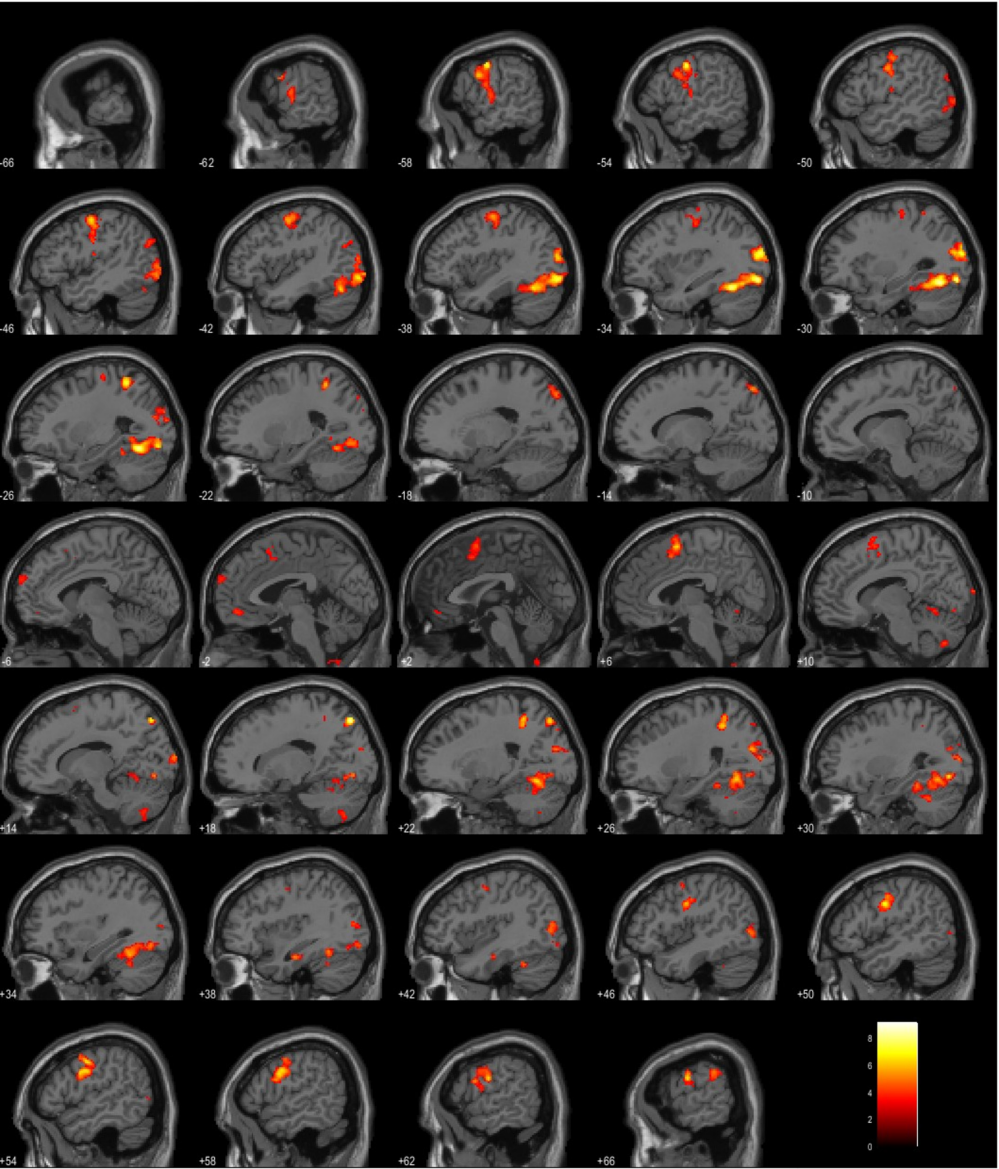
Combined language network obtained for Subject 9.

The authors evaluated the areas of activations for each task. Figures 4A–D and 5A–D show two example activation areas for all four tasks for Subjects 8 and 10. Supporting Information S1 shows the anatomical locations for each task and subject. There is no significant activation noted in either the left or right inferior frontal gyrus or superior temporal gyrus across all 15 subjects in the covert naming task. Given there was no verbal output, there was no activation of the dorsal stream/classical Broca's area. However, during the overt naming task, expected activations were found in the precentral and postcentral gyri along with temporal gyrus activation. Sentence completion tasks did not show any significant clusters in Subjects 1–3 at *p* < 0.001. Activations were noted in the left cuneus, left superior parietal lobule, and left temporal gyrus across seven out of 12 subjects. Right‐sided activations were noted in the pre and postcentral gyri. PPTT showed bilateral activations across 15 subjects, often involving the right parietal lobule or supramarginal gyrus. This was not surprising as the semantic network has been shown to have bilateral representation with involvement of the ventral stream due to the requirement of lexical‐semantic processing (Binder et al. 2009). Based on the accepted threshold, Subject 3 did not show any significant clusters. When significance at the voxel level was lowered to *p* < 0.05, activation clusters were observed at the right thalamus and right cingulate gyrus. We have created supplementary figures for all participants for all tasks. We have attached them as Supporting Information S1: with figures 1–15 (A–D) representing the four tasks in order as covert naming, overt naming, sentence completion and PPTT.

A group level analysis was performed for all tasks. Supporting Information S2 shows the group level results for each task with significant clusters displayed. The corresponding ROIs have also been tabulated in Supporting Information S2.

#### 3.2.2 Combined Language Network Localisation

To understand the language network representation, a combined model was generated for each subject using positive contrast for all events across the four tasks and negative contrast for all control blocks. This was also used to calculate the LI (Ruff et al. 2008) across all four tasks. Table 4 shows the LI for each participant calculated based on the overall language network computed from all four tasks. Figure 6 shows an example of the combined language network obtained for Subject 9. Despite all subjects being right‐handed, four patients were found to be left hemispheric dominant, three patients were right hemispheric dominant and the rest were found to not have a dominant hemisphere. Supporting Information S4 shows the combined language network for all subjects.

**TABLE 4:** Laterality index (LI) for all subjects.

Subject	LI	Hemispheric dominance
1	−0.02	Neutral
2	0.04	Neutral
3	−0.04	Neutral
4	0.19	Left dominant
5	−0.10	Right dominant
6	0.19	Left dominant
7	−0.10	Right dominant
8	0.00	Neutral
9	0.07	Neutral
10	0.03	Neutral
11	−0.15	Right dominant
12	0.01	Neutral
13	0.10	Left dominant
14	−0.14	Right dominant
15	0.00	Neutral

#### 3.2.3 Signal Stability

A signal stability analysis was performed to understand how the group level ROIs (Supporting Information S3) behaved in each subject. After performing group level analysis and extracting the significant ROIs, a time series was generated for each ROI for each task. Temporal signal to noise ratio (tSNR) was then computed. These results have been tabulated in Supporting Information S3.

In addition to assessing signal stability, task activations were analysed for stability in six regions: the left inferior frontal gyrus, left precentral gyrus, left middle frontal gyrus, left supramarginal gyrus and left superior temporal gyrus. These structures comprise key components of the language network (Friederici and Gierhan 2013; Hagoort 2014). In this analysis, ROI masks were created for each area to facilitate the assessment of activation patterns in these areas associated with each task. Supporting Information S5 presents the contrast estimates at voxel of maximal activation for each ROI for each task.

## 4 Discussion

With advancements in neurolinguistics (Collée et al. 2023; De Martino et al. 2021; Nozari 2023; Zacà et al. 2013) and data from intraoperative cortical mapping, language is now understood to have a much more complex, with dual hemispheric representation beyond the classical Broca's and Wernicke's areas. To optimise clinical outcomes in patients with tumours in language areas, both preoperative assessment and intraoperative cortical mapping are required. Currently, the gold standard of language assessment is performing a series of tasks intraoperatively while the tumour is resected. The limitations of performing extensive tasks depend on the ability of the patient to stay awake with adequate participation in these tasks. fMRI provides an alternative to obtaining maximal information preoperatively, allowing the assessor to choose a selective series of tasks.

Due to intrinsically low signal to noise ratio in fMRI, multiple repeated stimulations during each task‐based fMRI are required to obtain robust results. Unlike motor or somatosensory tasks, a single task is unable to identify the ‘language network’. Studies focusing on task‐based fMRI do not assess the feasibility of completing an assessment of the language network within given time constraints. In a clinical setting, the duration of performing complete functional and structural imaging is also limited due to resource constraints and often pre‐existing neurological deficits in patients. In this study, the authors evaluated the ability to complete a language assessment in a reasonable time frame, using a set of predefined four tasks. All participants completed the functional component of the scan in less than 26 min, with an additional 30 min required for completion of structural imaging and other MRI sequences such as resting state fMRI and diffusion tractography. Patients would also require contrast enhanced scans and even with those additional sequences, the expected time of scan duration would be under 2 h. This is reassuring as we can expect a patient to complete the preoperative MRI in a reasonable time, thus reducing the chances of discomfort from prolonged lie, patient movements resulting in significant motion artefacts, or poor cooperation with the tasks.

Various studies using more than one language paradigm often document the duration of each task, often ranging between 3 and 5 min each. By adding more tasks, while it generates more information regarding the language network, it is increasing the complexity of the fMRI paradigm, prolonging the duration of the entire scan, and eventually the reliability of the results which would be impacted by the participant fatigue and cooperation in the scanner. A study by Kuan et al. (2024) that intended to look at machine learning in task‐based fMRI used a seven‐task‐based language paradigm. The time duration allocated in total to the seven tasks was 29 min but this does not describe the actual time taken during turnover between tasks and other images that need to be acquired in between tasks. A study (Heath et al. 2012) assessing using of picture naming primer prior to a semantic task required 26.95 min for an individual. Other studies (Park et al. 2020; Unadkat et al. 2019) using a combination of picture naming, auditory comprehension, and sentence completion task averaged from 3 to 4 min per tasks. These studies do not mention the eventual time taken by the individual for each task and overall scan timing. In this study, the authors calculated the time taken for the completion of each task for each participant (Table 2) which included any additional time that may be needed to switch from one task to another. This allowed for incorporation for other factors that would influence the duration of the scan such as software glitches, delayed display of images after input, and response time where participant response is expected.

The neural mechanisms underlying covert and overt naming are largely analogous, except for the additional regions engaged during articulation: these include four right‐hemispheric areas—the mid superior temporal gyrus, medial and lateral cerebellum and supplementary motor area (SMA)—as well as 11 left‐hemispheric regions, such as the posterior inferior frontal gyrus, ventral precentral gyrus, SMA, mid and posterior superior and middle temporal gyri, posterior temporal fusiform gyrus, anterior insula, thalamus and medial cerebellum (Indefrey and Levelt 2004). Results in this study are consistent with the existing literature as shown in Figures 4, 5 and Supporting Information S1 and S2 at both‐ individual and group‐level analyses. Covert naming task allows assessment of conceptual processes and overt naming additionally assesses the ability to articulate words. When working with patients with severe aphasia, it is essential to determine whether deficits stem from impairments in conceptual processing or word production. Accordingly, both covert and overt naming tasks have been incorporated into this assessment protocol. For patients who exhibit minimal or no aphasia, the covert naming task may be omitted from the paradigm to further shorten the duration of the fMRI protocol.

fMRI has often been criticised for lack of concordance with the intraoperative cortical mapping and using tasks that cannot be reciprocated intraoperatively due to time constraints (Austermuehle et al. 2017; Bajracharya and Peelle 2023). A recent meta‐analysis on the use of fMRI for language localisation in brain tumours showed that the sensitivity and specificity for Broca's area were 84% and 74%, respectively, and for Wernicke's area were 71% and 73%, respectively (Metwali et al. 2019). The authors evaluated the use of commonly used intraoperative tasks for preoperative evaluation that would provide maximal insight into the language network of the individual. In this study, the authors chose the tasks that can be easily reciprocated during awake surgery—object naming, sentence completion, and semantics association using PPTT. Other than the sentence completion task, the rest required no translation and can be easily replicated in any other language. The tasks focused on evaluating beyond the classical language model (Hickok 2022; Kargar and Jalilian 2024) as described in the introduction, focusing on evaluating the near bilateral representation of the language. The authors chose a very stringent *p*‐value of 0.001 for voxel level significance to prevent over‐reporting of activation areas. The authors acknowledge that the next step would be to correlate the results with direct cortical stimulation for patients with tumours, and this study does not address that.

By using covert and overt naming tasks, the authors evaluated the results of visual input with the overt naming allowing assessment of the motor output of the language. Healthy volunteers in this study did not have any difficulty naming the objects. By placing a MRI compatible microphone during the scanner, it is possible to listen to the correct and incorrect responses to the stimulus presented to the patients. Any hesitancy in naming or lack of response would be assessed. As some patients are likely to have word finding difficulties or dysphasia, by assessment of both covert and overt naming allows accurate assessment of the visual processing and final output. Intraoperatively, it is not possible to assess patient's functional areas using covert naming as the surgeon would be unable to tell if the patient's thought process is interrupted during stimulation of the regions of concern (Austermuehle et al. 2017; Chang et al. 2018; Collée et al. 2023; De Martino et al. 2021). Studies evaluating overt picture naming in patients with epilepsy (Rolinski et al. 2020; Trimmel et al. 2019) have shown that the temporal language areas are susceptible to dysfunction and re‐organisation. Results from lesional studies such as stroke (Martin et al. 2005; Skipper‐Kallal et al. 2017) have evaluated overt naming in fMRI to assess recovery in speech. These studies continue to emphasise the role of the superior temporal gyrus in overt naming with roles of pars opercularis affecting naming ability. Zhuang et al. (2014) showed the functional differentiation of frontotemporal systems for processing spoken language. As seen in Supporting Information S1 there was minimal activation of the classical language areas (Broca's and Wernicke's) during covert naming despite activations seen in the parietal lobe and other association areas. This is in keeping with the absence of involvement of the dorsal inferior frontal gyrus due to lack of articulation. During overt naming, significant activation across superior and inferior temporal gyri, precentral and postcentral gyri was in keeping with expected language areas involved during processing and speech production, in keeping with current literature (Barch et al. 1999; Martin et al. 2005; Shmuelof and Zohary 2005; Skipper‐Kallal et al. 2017). These results were visible on the individual language maps where these cortical areas were easily identified (Figures 4 and 5 and Table S1).

The sentence completion task involves reading comprehension, semantic processing and word retrieval. Unlike the covert and overt naming tasks that can be translated into any language, the sentence completion task will require translation and contextual changes, if the paradigm is being used for a different language. In this study all the volunteers used English as their first language. Ashtari et al. (2005) showed that silent sentence completion provided activation of the posterior left temporal cortex extending into the inferior parietal lobule. This task has been shown to assess receptive language (Zacà et al. 2013) and when combined with tasks such as silent word generation, has been shown to provide adequate information on language lateralisation. Results in our study were consistent with the literature (Black et al. 2017; Salek et al. 2017). Activations beyond the classical language areas were seen including the middle frontal gyrus, cuneus and parietal lobule, consistent with the language network being more diffused than localised to specific cortical areas. Both dorsal and ventral streams are critical for syntax (Griffiths et al. 2013), and sentence completion provides an adequate method of assessment for syntax preservation.

The authors did a formal evaluation of Pyramids and Palm Tree Test (PPTT) in this study using fMRI. PPTT is a semantic memory test that measures the capacity to access detailed semantic information about pictures, necessary for the identification of the analogies, which link conceptually two perceptually and functionally distinct entities. While this has been sporadically used in real‐time neuropsychology testing for intraoperative assessment (Howard et al. 1992; Martin and Chao 2001; Moritz‐Gasser et al. 2013; Rami et al. 2007), this study provides an early assessment of using this test as a preoperative fMRI paradigm. Occipital lobe involvement was regularly noted due to the constant visual stimulation. Involvement of the supramarginal gyrus and superior parietal lobule was consistent in keeping with the association areas involvement to complete the task (Binder et al. 2009; Giovagnoli et al. 2005; Martin and Chao 2001; McGeown et al. 2009; Price et al. 1999).

Handedness alone cannot reliably predict functional areas, highlighting the importance of the language function assessment using a large battery of tests regardless of tumour location (Duffau et al. 2008). Left‐handed or ambidextrous individuals have a higher likelihood (22%–30%) of right‐brain dominance for language compared to right‐handed people (4%–12%) (Voets et al. 2025). Studies using direct electrical stimulation (Martín‐Monzón et al. 2024; Vassal et al. 2010; Vilasboas et al. 2017) in right‐handed patients with right‐sided lesions (in language areas) found articulatory, naming and semantic disorders, underscoring that surgeons should not rely solely on handedness to determine hemispheric dominance.

fMRI offers a non‐invasive approach to assessing language lateralisation. The calculation of the LI is a straightforward method for determining dominance, though it is highly task‐dependent, particularly in language studies where different tasks target distinct domains (Seghier 2008). Task repetition has the potential to influence fMRI‐based measures of language lateralisation, sometimes resulting in increases in apparent bilaterality (Lohmann et al. 2004). According to Ramsey et al. (2001) LI derived from combined task analysis (antonym generation, verb generation and categorical naming) demonstrates greater consistency across varying statistical thresholds for signal change significance in fMRI data when compared to individual task analysis. For this study, all four task results were combined to generate the LI. These tasks spanned across various domains of language including comprehension, articulation and semantic processing. Only two volunteers were found to be left hemisphere dominant in this study.

Fedorenko et al. (2010) highlighted the value of functionally robust localisers in language assessment using a set of predetermined ROIs. However, functional localisers may not be practical for patients with significant anatomical changes from lesions, requiring tailored assessments rather than fixed ROIs. Providing this ultra‐rapid paradigm balances both time constraints and individualised assessment of the language network. Reliance on a single task to understand hemispheric dominance can result in pitfalls (Parker et al. 2022; Rolinski et al. 2020; Wegrzyn et al. 2019). This can be seen in this study where the hemispheric dominance differed across the individuals despite being conventionally right‐handed on the original assessment. The authors want to highlight the distributive nature of the language network. Reliance on single task to localise the language network can lead to underestimation of the areas involved. This is prudent in patients with brain lesions in which using a single task may result in underestimation of language representation on the traditional non dominant hemisphere leading to significant post‐operative neurological deficits in communication and affecting the quality of life. The interpretation of signal stability in task‐based fMRI remains an area of ongoing discussion, as BOLD responses are inherently variable across individuals. This variability often reflects genuine neural dynamics rather than mere noise. As demonstrated in Supporting Information S3, each subject exhibited high tSNR within the group‐level ROIs. Further research is necessary to determine whether these ROIs exhibit comparable behaviour in patients with underlying pathology.

As shown in Supporting Information S5, the activation patterns observed across established language‐network ROIs for each task align with findings reported in the existing literature. Covert and overt naming elicited comparable activation patterns across all ROIs except ones responsible for articulation, indicating that both tasks engage similar neural mechanisms (Indefrey and Levelt 2004). The left supramarginal gyrus activation observed during sentence completion is consistent with its established role in reading across task contexts (Stoeckel et al. 2009). Likewise, the involvement of the superior temporal gyrus aligns with prior descriptions by Salek et al. (2017) and is replicated in the present findings. The fusiform gyrus supports high‐level visual recognition, including faces, objects and visual word forms (Weiner and Zilles 2016). Its activation during the non‐verbal pyramids and palm trees task is therefore not unexpected. Activations were observed in the left inferior frontal gyrus and left superior temporal gyrus across all four tasks underscoring the significance of the traditional Wernicke's and Broca's areas in multiple language domains (Lacey et al. 2017; Moriai‐Izawa et al. 2012; Salek et al. 2017).

All testing paradigms used in this study can be easily translated and repeated intraoperatively. There are significant benefits as the patients would not require additional training to perform a different set of tasks for fMRI and for intraoperative testing. The neurosurgical team can assess and train in a single session prior to performing any preoperative imaging. Furthermore, time allocated to each event in the task presented is the same as that in intraoperative testing corresponding to the time used for direct cortical stimulation, allowing seamless translation between the two.

## 5 Limitations

This study did not evaluate the paradigms in patients with brain lesions. Intra‐procedural cooperation with tasks and task performance in the setting of pre‐existing deficits has not been evaluated in the study. The study does not provide any results in patients speaking languages other than English.

## 6 Conclusions

The study assesses the feasibility of a rapid four‐task‐based fMRI paradigm evaluating the language network in healthy volunteers. The tasks are completed in a reasonable time duration and provide maximal information about the language network—both from classical‐ and dual‐stream‐model points of view. As these tasks can be translated easily into intraoperative testing during awake surgeries, these paradigms pave the way for providing an individualised information of the language network. Further research is needed to understand any limitations in patients with pre‐existing neurological deficits and brain lesions.

## Author Contributions

**Swati Jain:** conceptualization, data curation, formal analysis, investigation, project administration, software, validation, writing – original draft. **Emmanuel A. Stamatakis:** conceptualization, data curation, formal analysis, investigation, methodology, project administration, supervision, validation, writing – review and editing. **Stephen J. Price:** conceptualization, funding acquisition, supervision, writing – review and editing.

## Funding

This study was supported by National Institute for Health and Care Research (NIHR), Career Development Fellowship (CDF‐2018‐11‐ST2‐003).

## Conflicts of Interest

The authors declare no conflicts of interest.

## Supporting information


**Data S1:**Task specific activations for each subject.

**Data S2:**Group level activations for each task have been shown on axial slices. Corresponding ROIs have been documented under each task.

**Data S3:**Signal stability for each task for each subject in each task‐level group level ROIs. Signal to noise ratio (SNR) has been documented for each subject for each task.

**Data S4:**Combined language network for each subject.

**Data S5:** Activations for each task in known language nodes. Contrast estimates have been shown in these language nodes.

## Data Availability

The data collected in this research study are bounded by ethics and are not available to be shared. The authors can be contacted for further information.
