# Supplementary material for: A Rapid fMRI Paradigm for Localisation of the Language Network
Source: Eur J Neurosci. 2026 Mar 6;63(5):e70448. doi: 10.1111/ejn.70448 (PMC12964186; doi:10.1111/ejn.70448)

Subject 1

Covert Naming

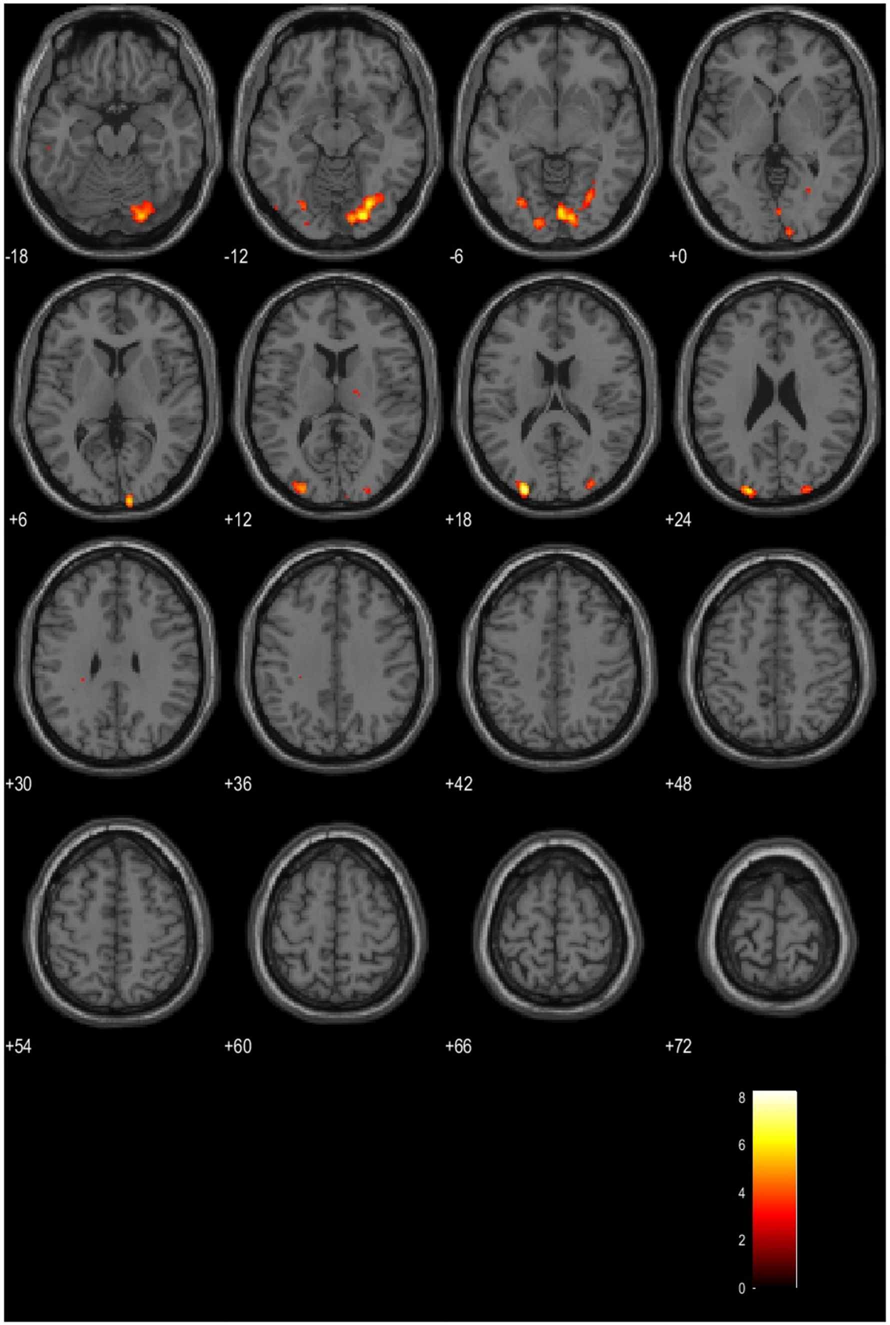

Overt Naming

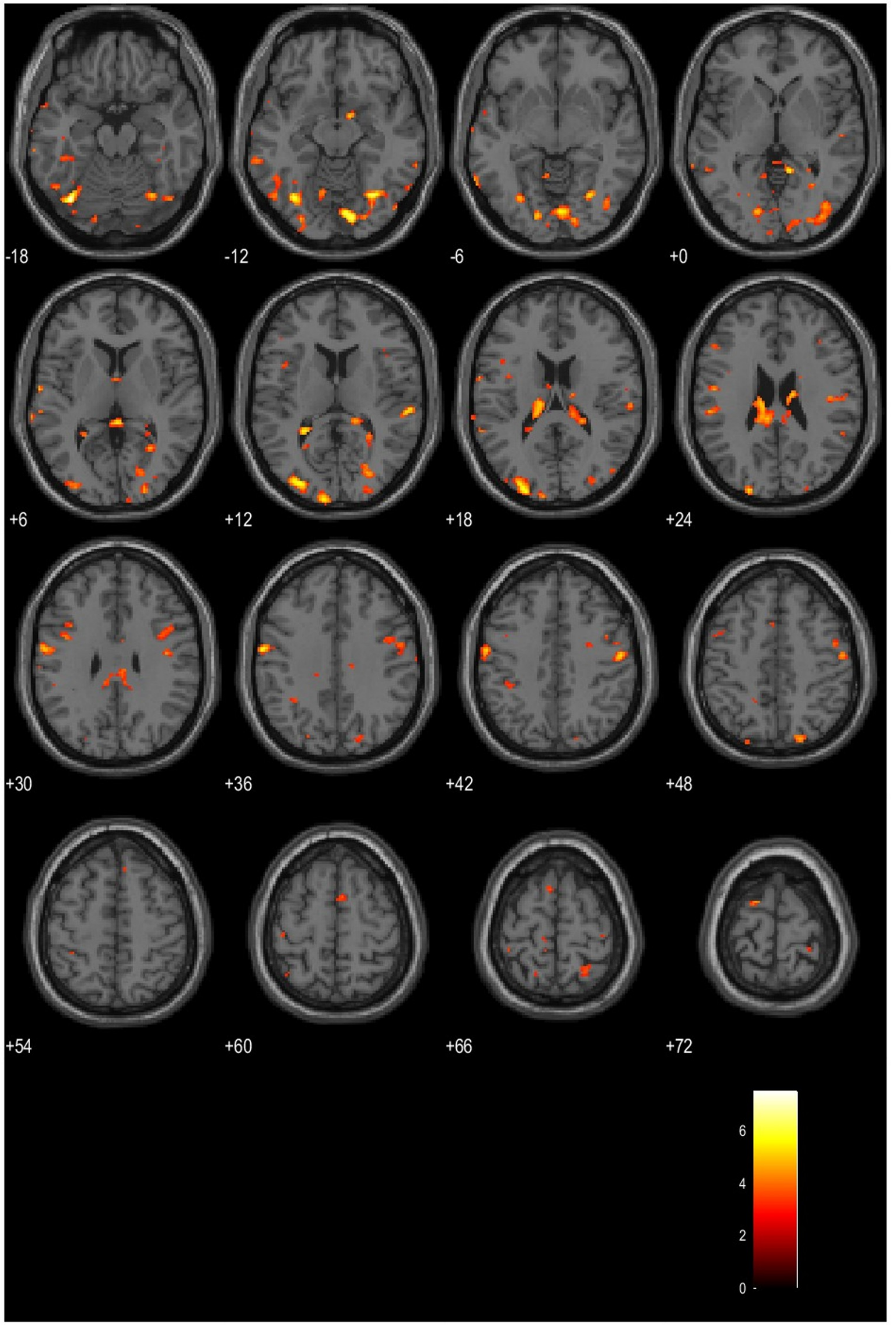

Subject 2

Covert Naming

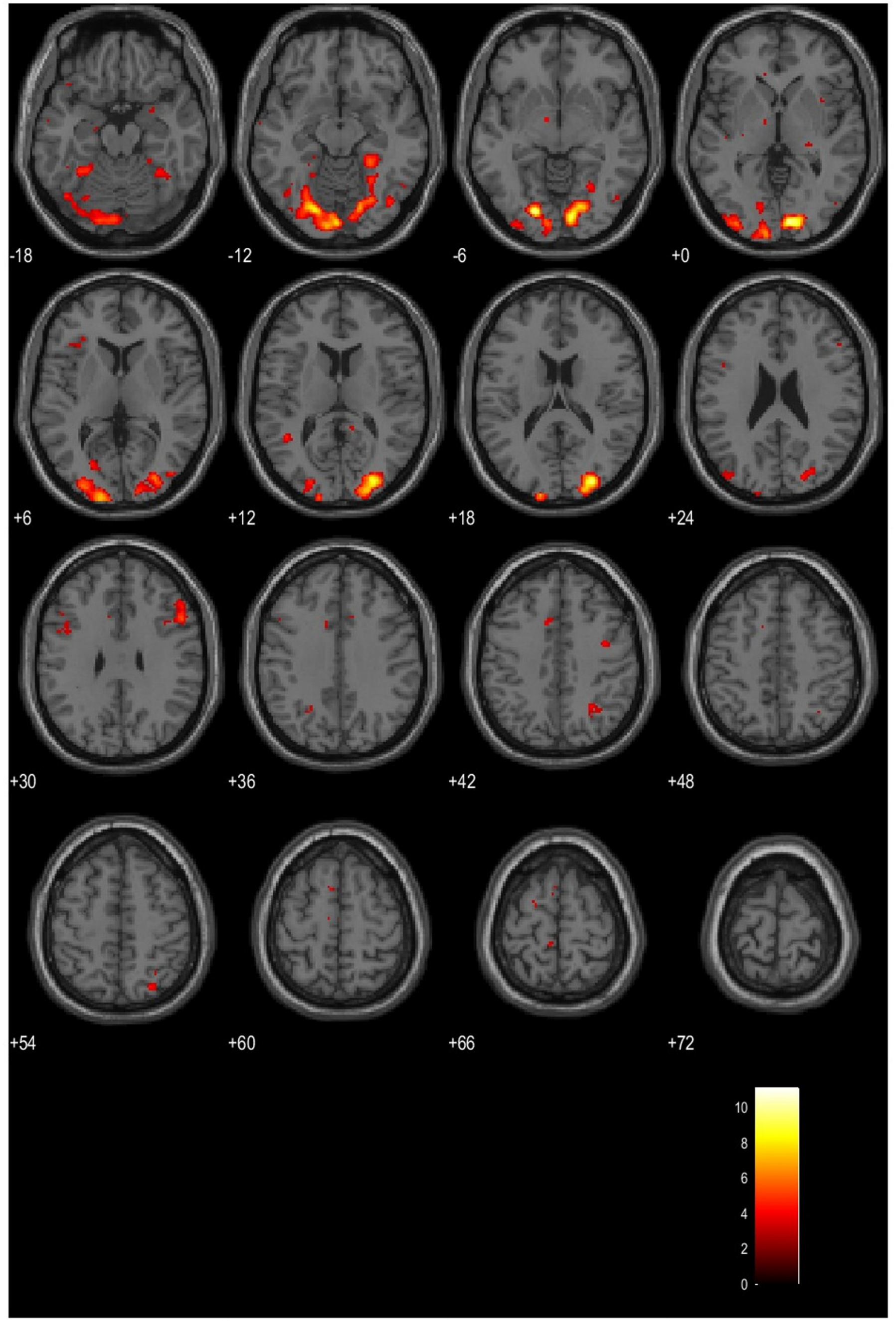

Overt Naming

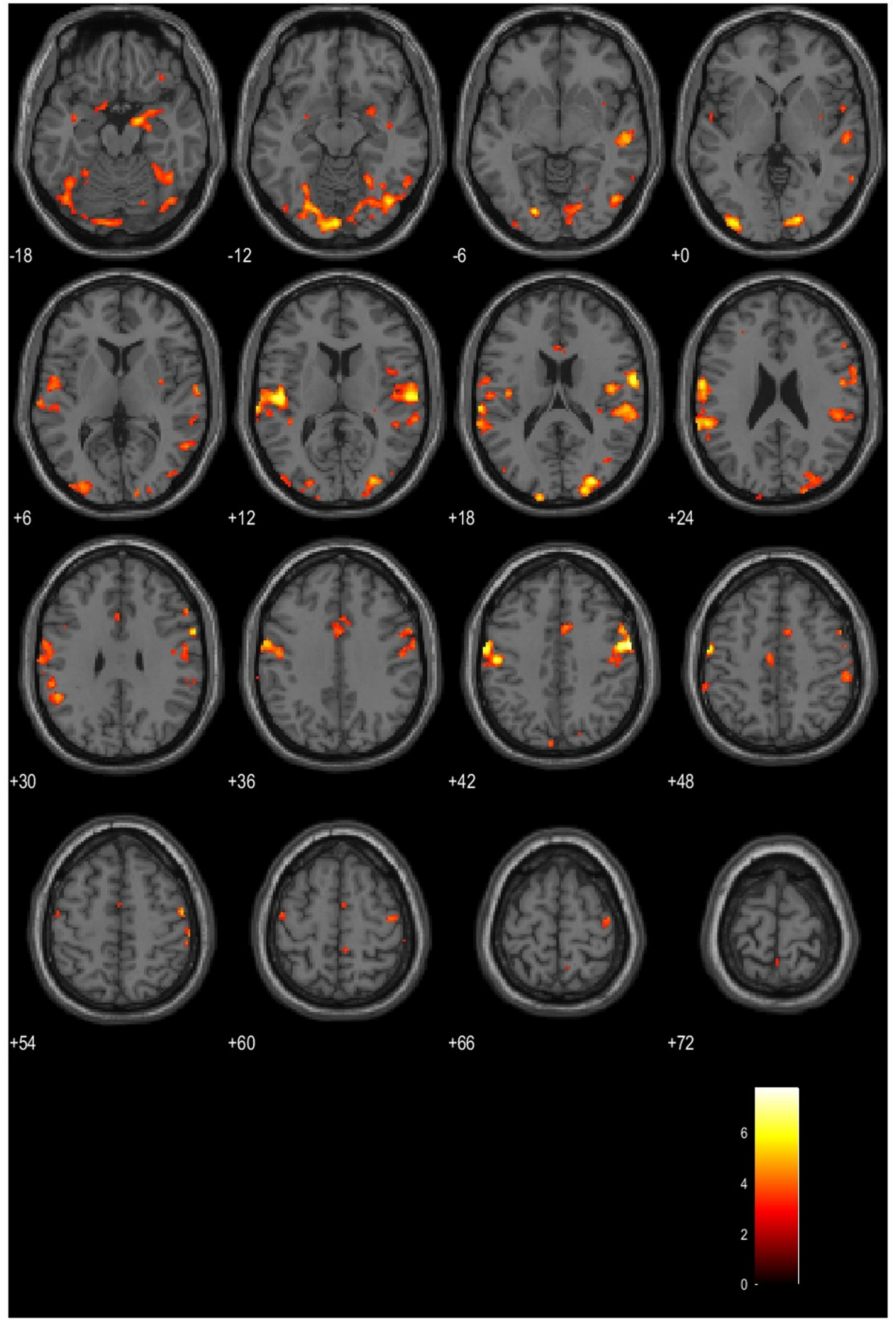

Sentence Completion

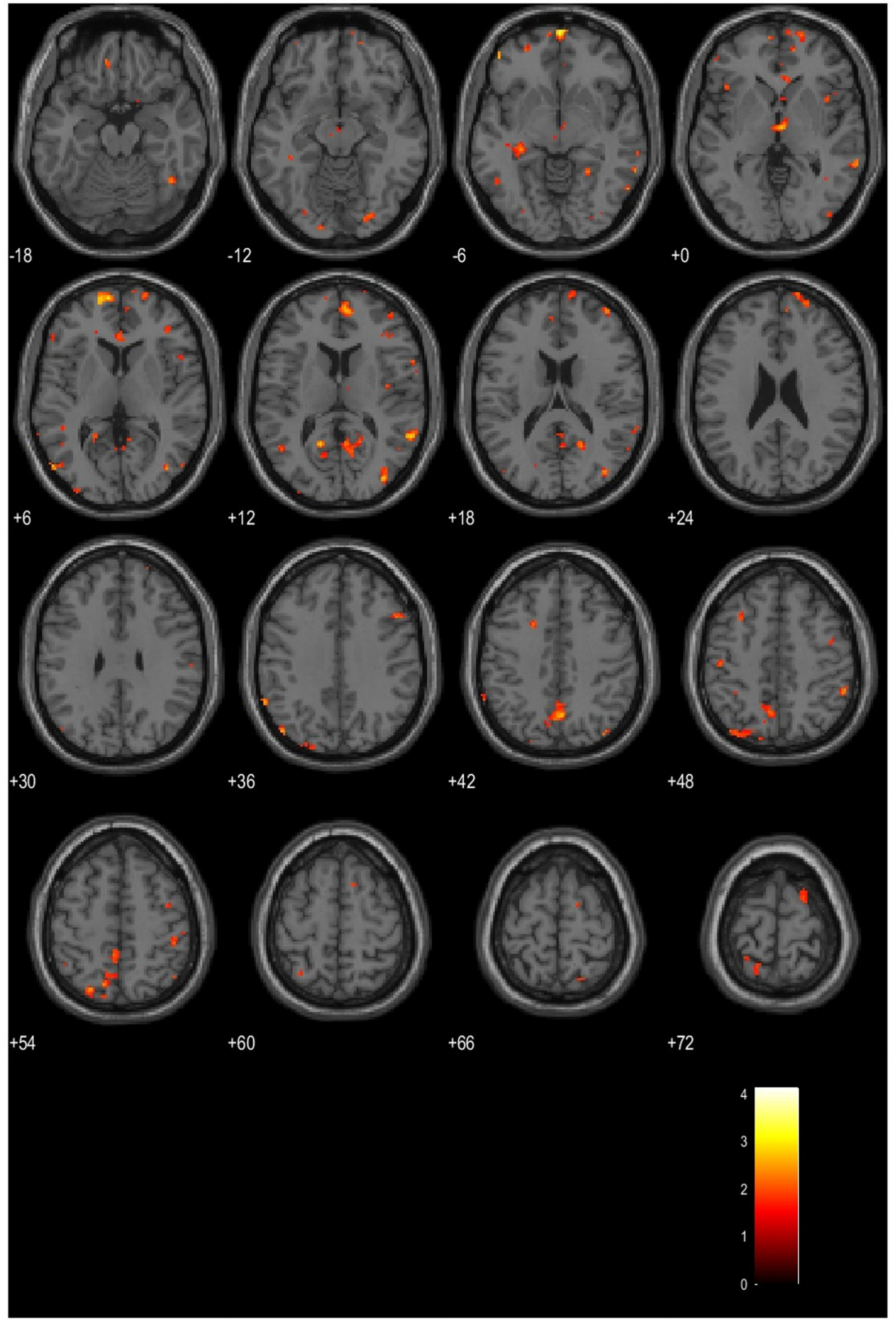

Semantics Association

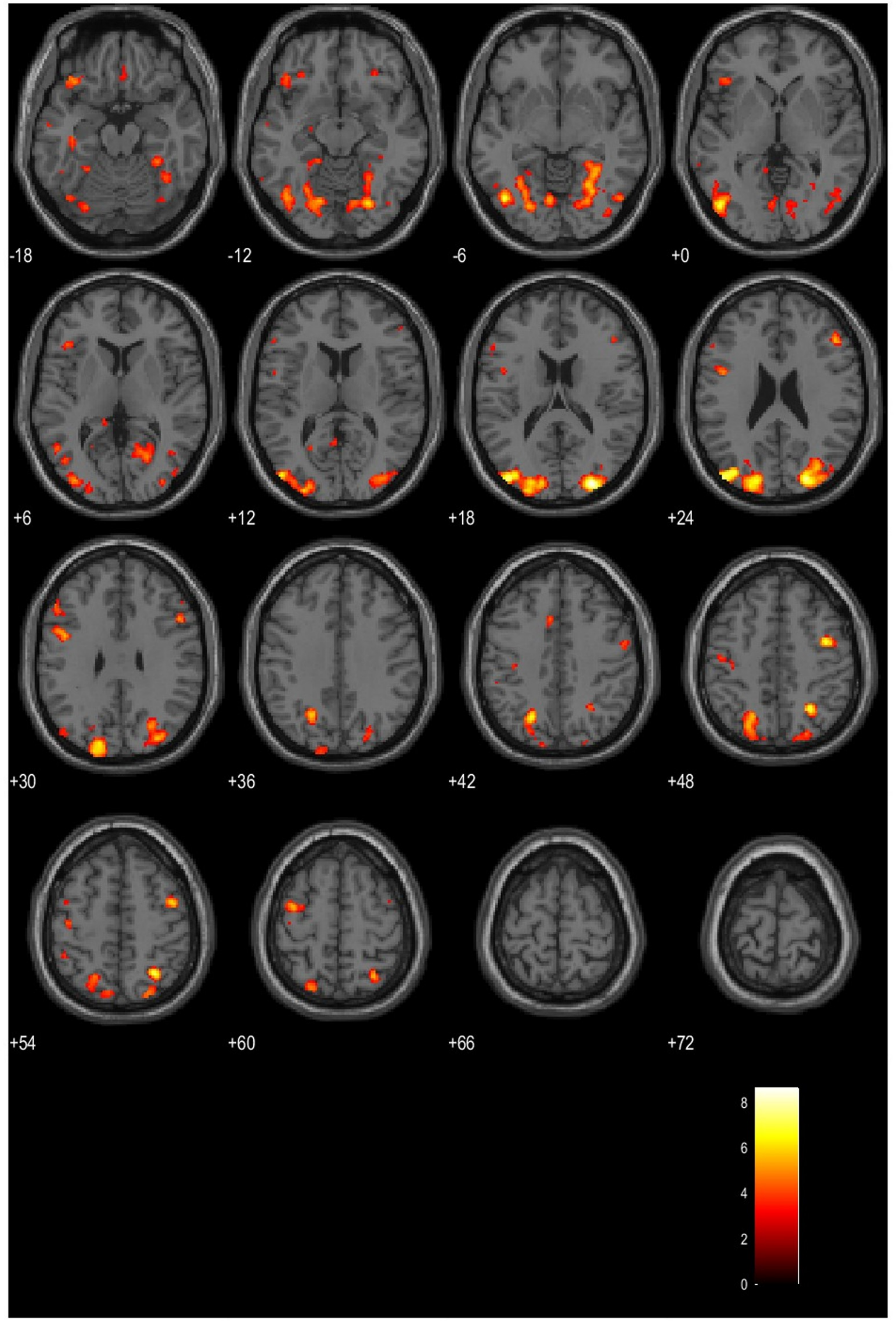

Subject 3

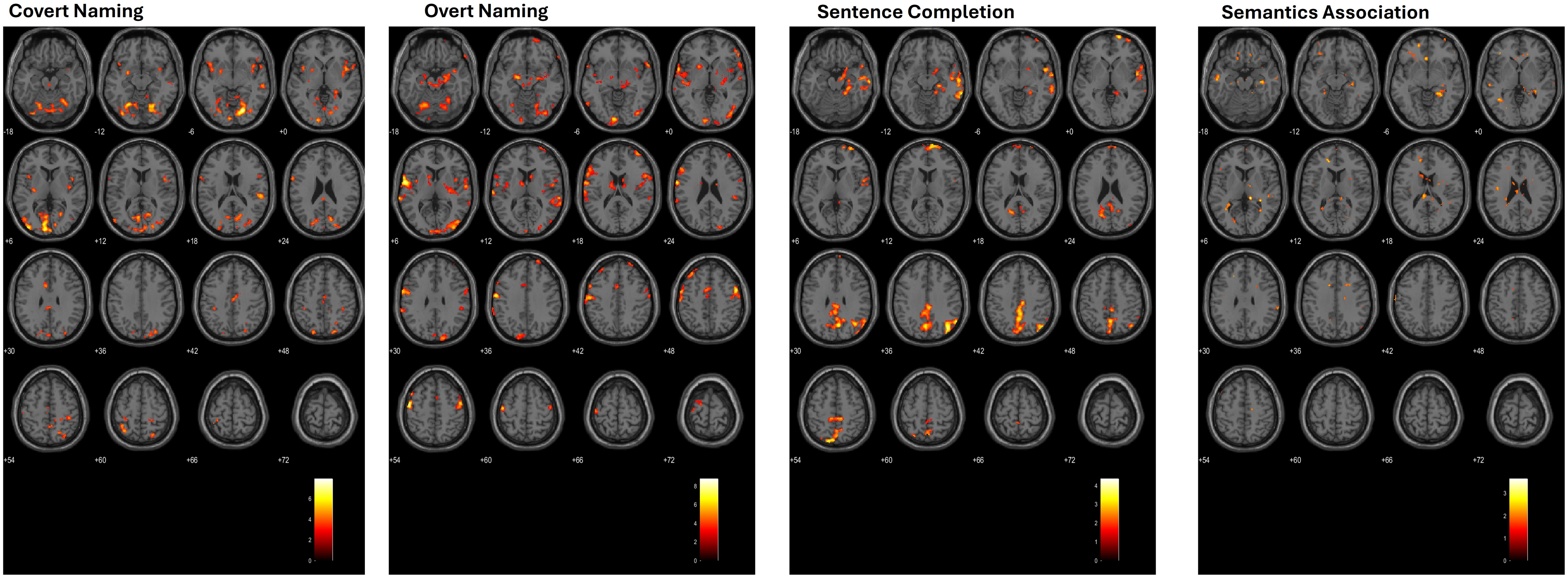
Covert Naming
Overt Naming
Sentence Completion
Semantics Association
-18
-12
-6
+0
+6
+12
+18
+24
+30
+36
+42
+48
+54
+60
+66
+72

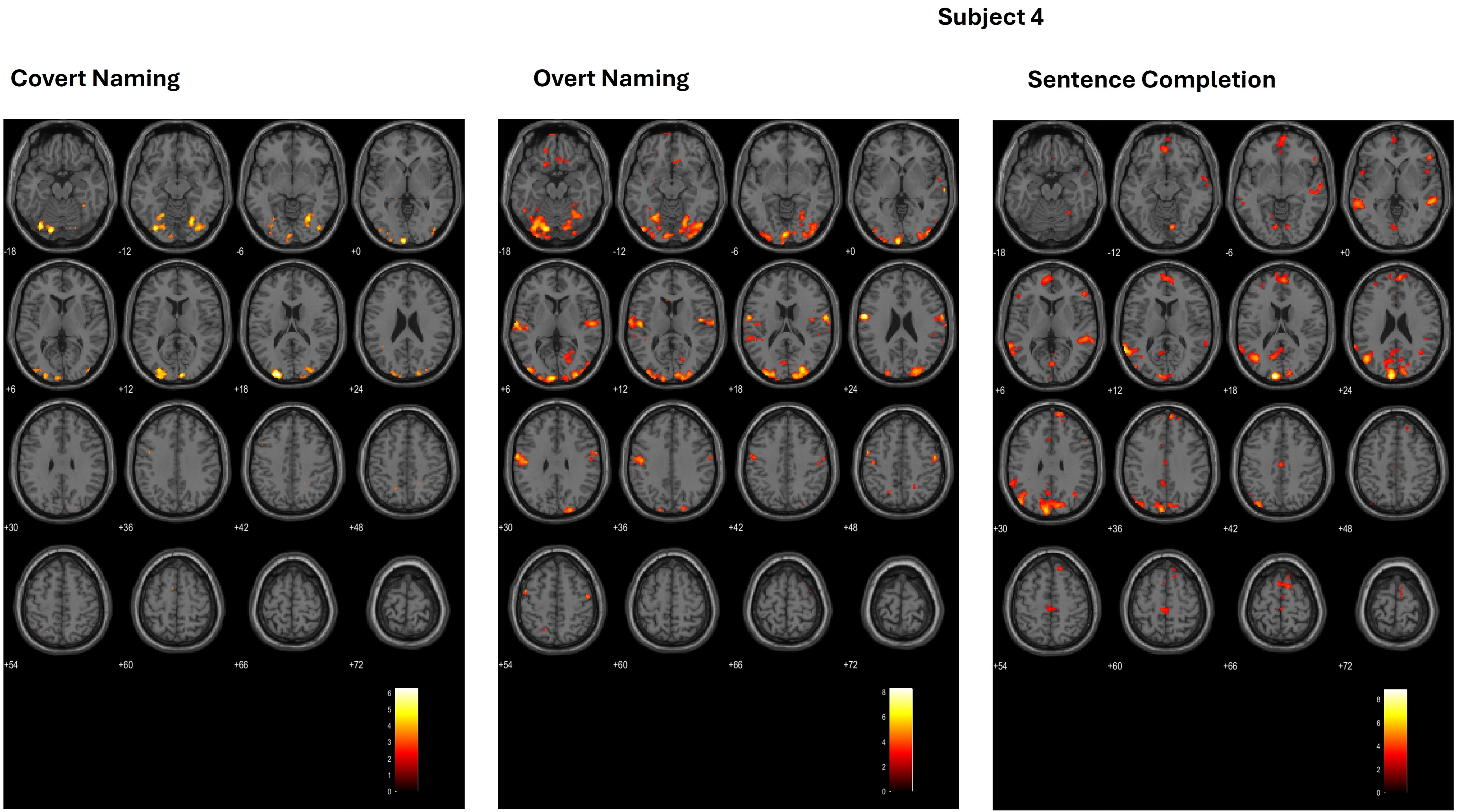
Subject 4
Covert Naming
Overt Naming
Sentence Completion

Subject 5

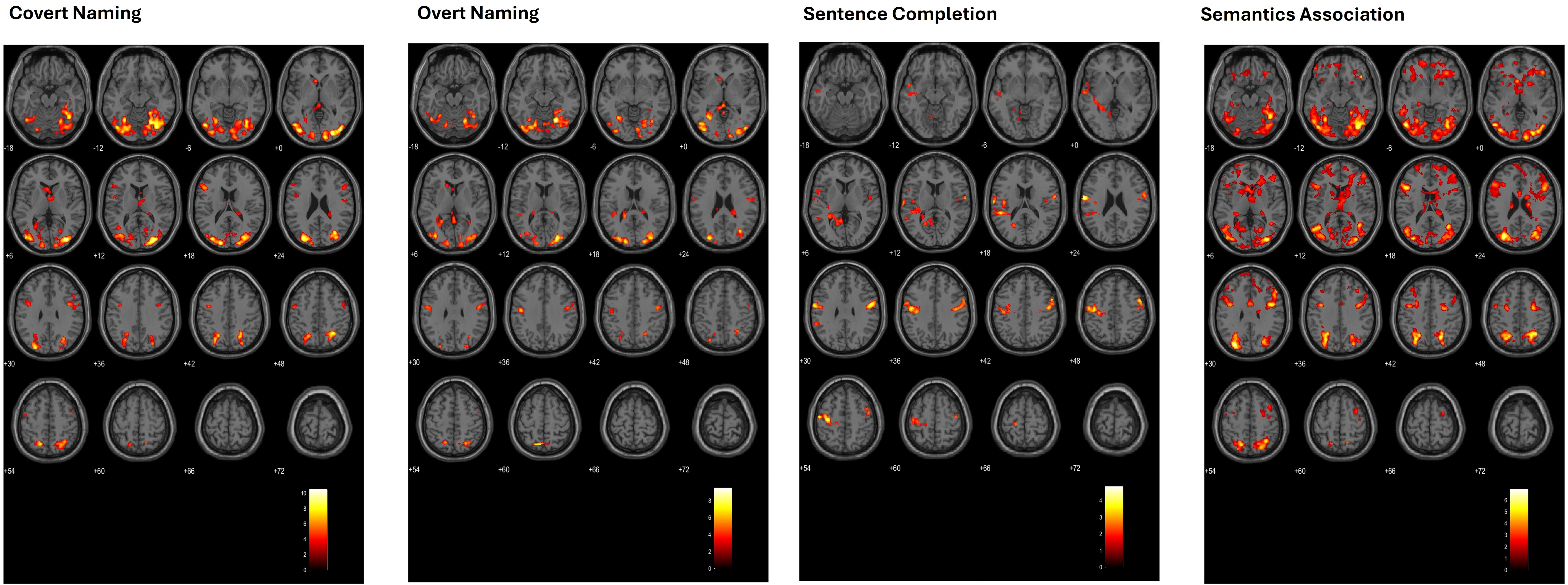
Covert Naming
Overt Naming
Sentence Completion
Semantics Association
-18
-12
-6
+0
+6
+12
+18
+24
+30
+36
+42
+48
+54
+60
+66
+72

Subject 6

Covert Naming

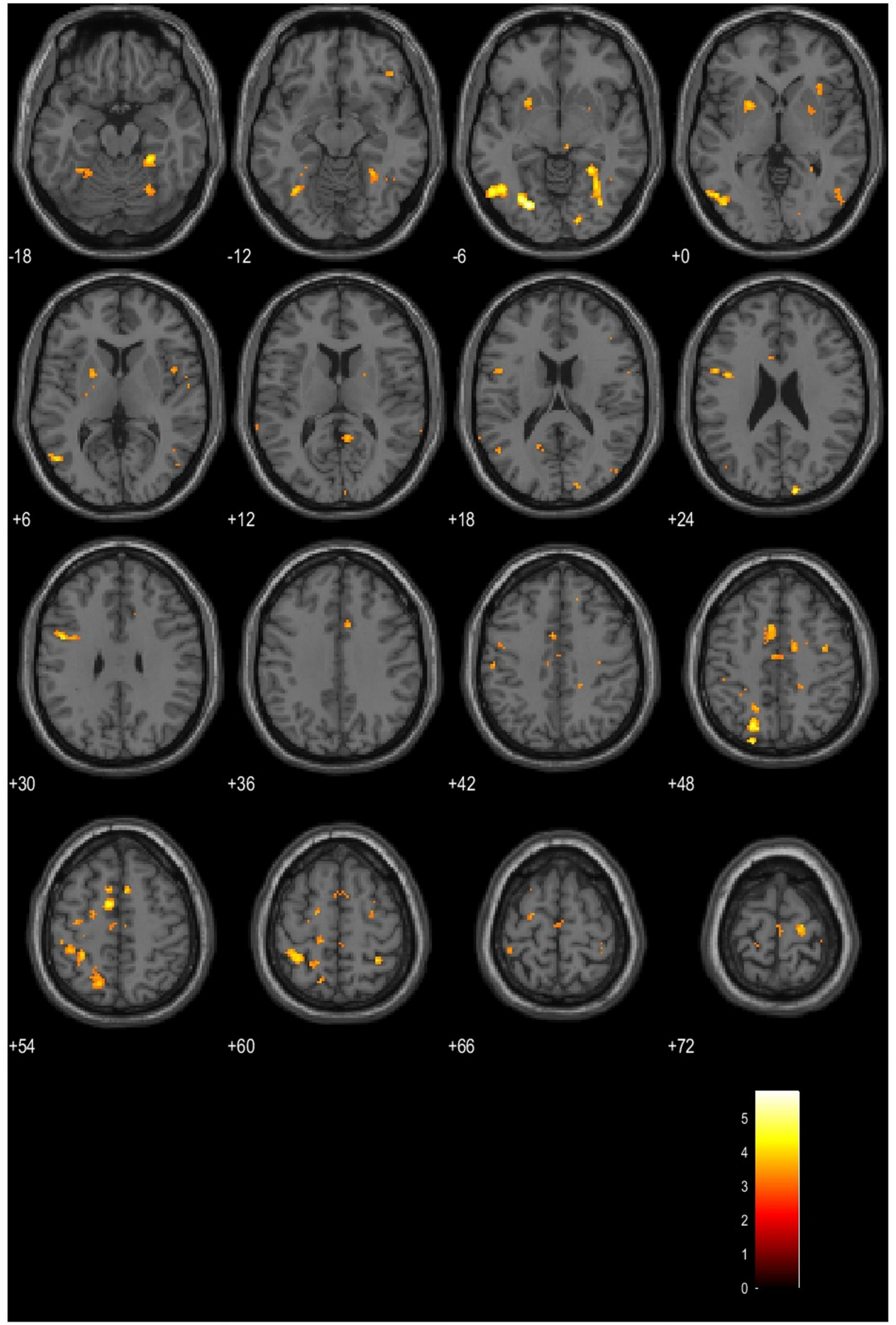

Overt Naming

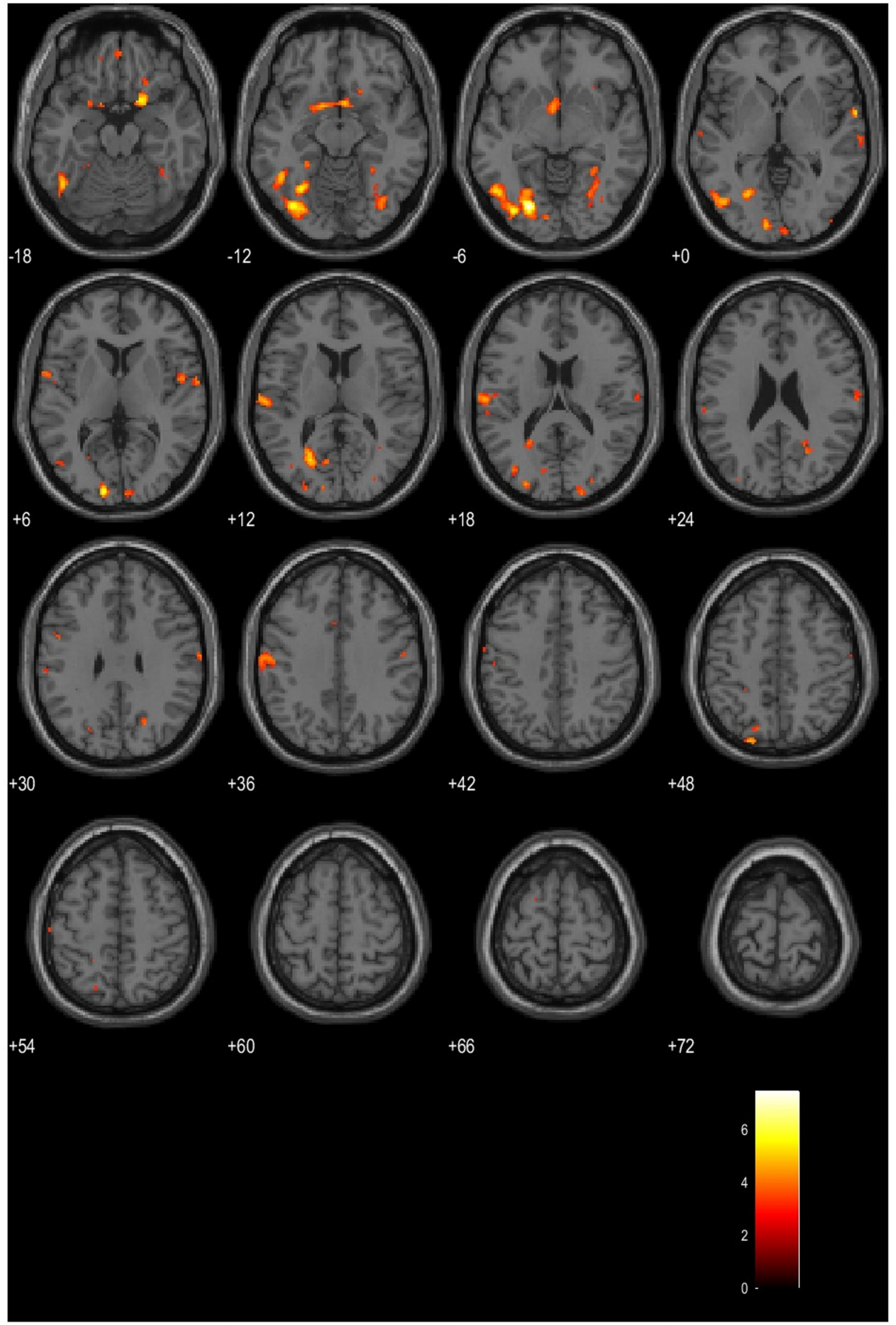

Sentence Completion

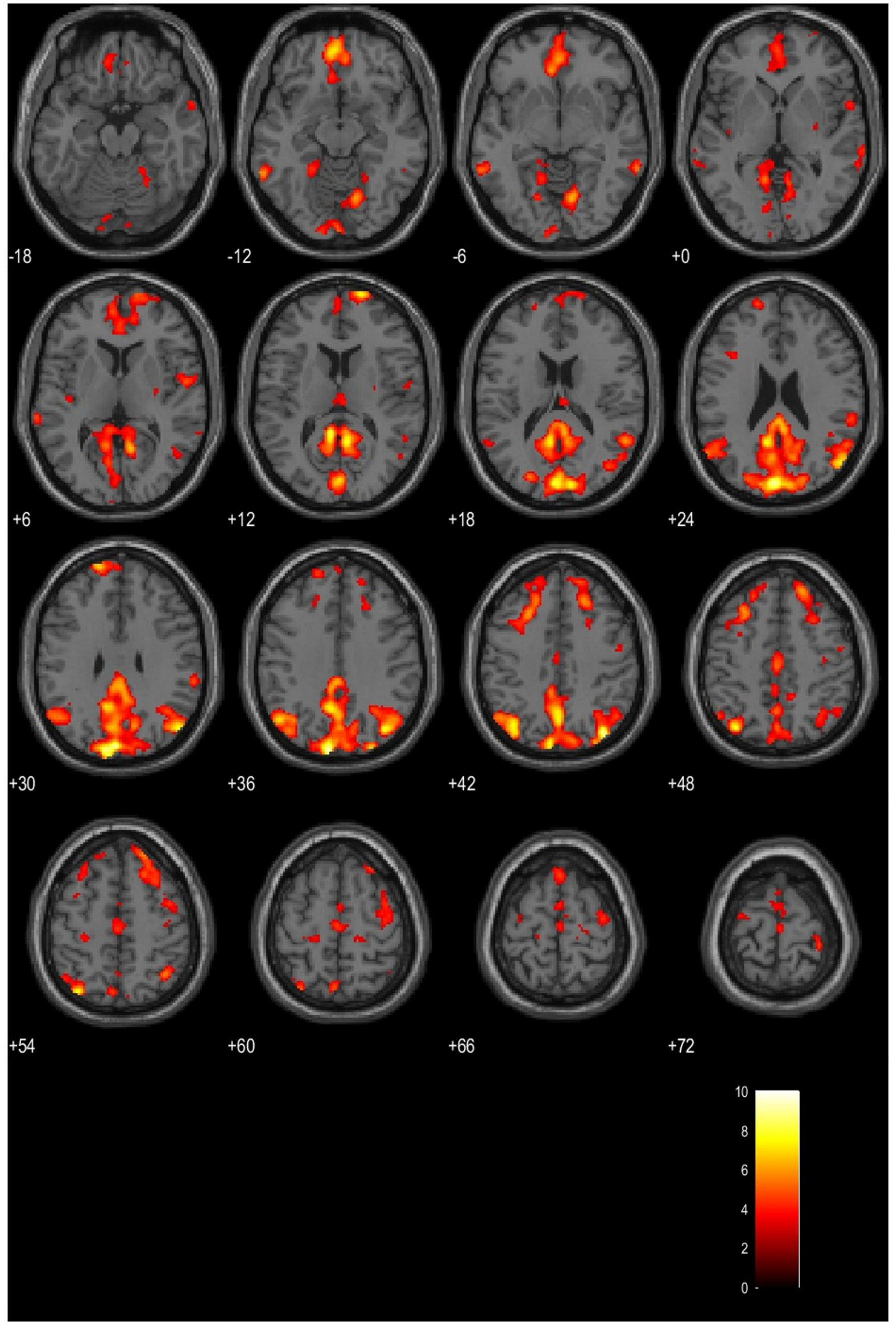

Semantics Association

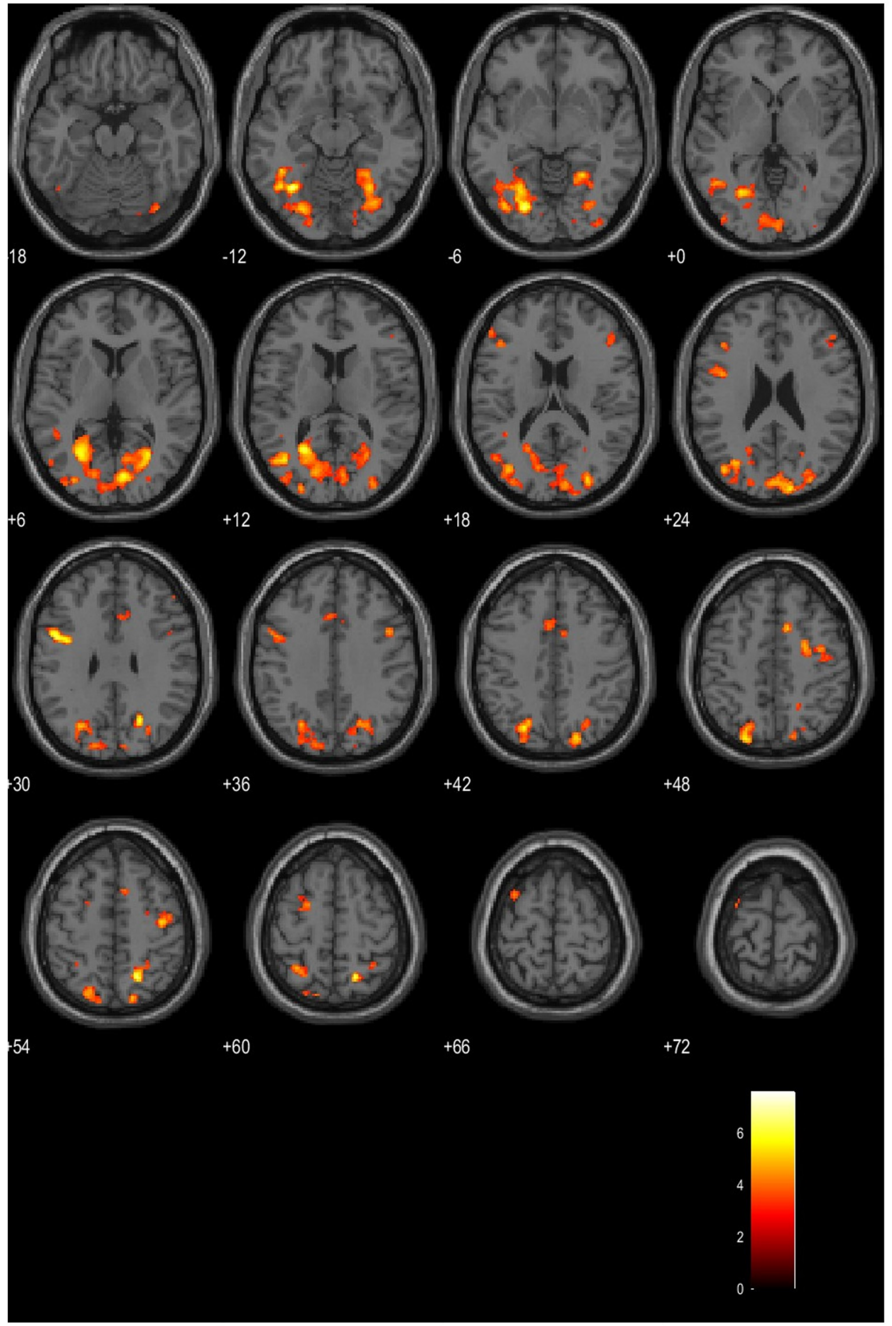

Subject 7

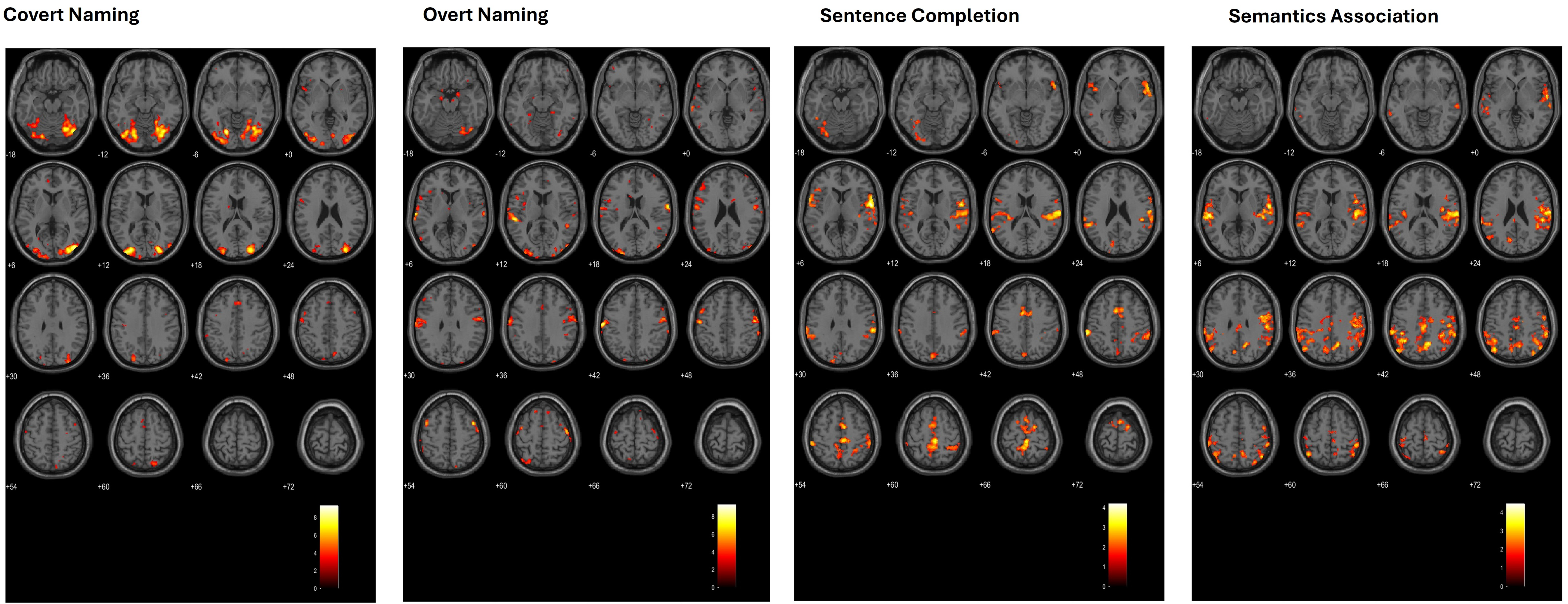

Covert Naming
Overt Naming
Sentence Completion
Semantics Association
-18
-12
-6
+0
+6
+12
+18
+24
+30
+36
+42
+48
+54
+60
+66
+72

Subject 8

Covert Naming

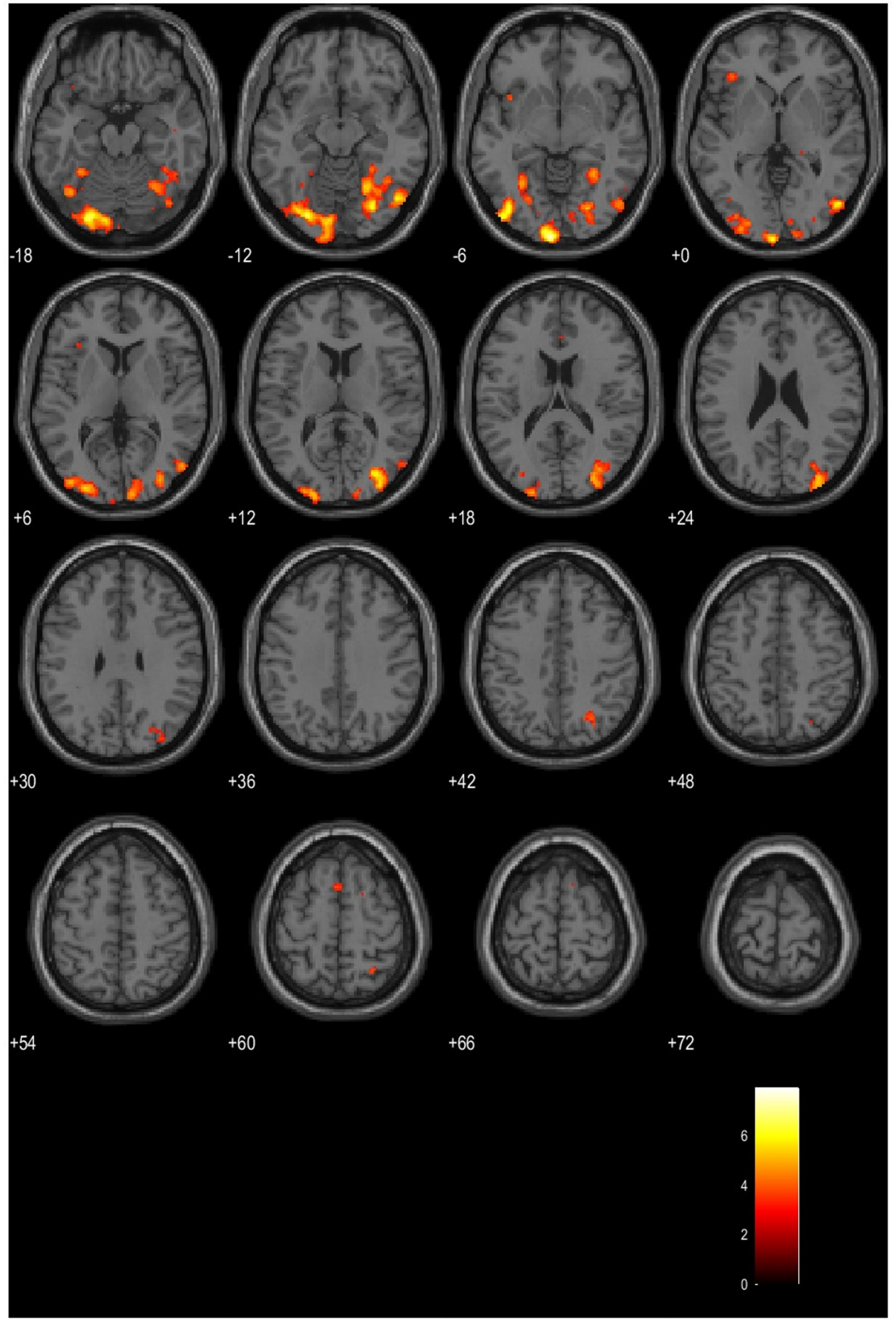

Overt Naming

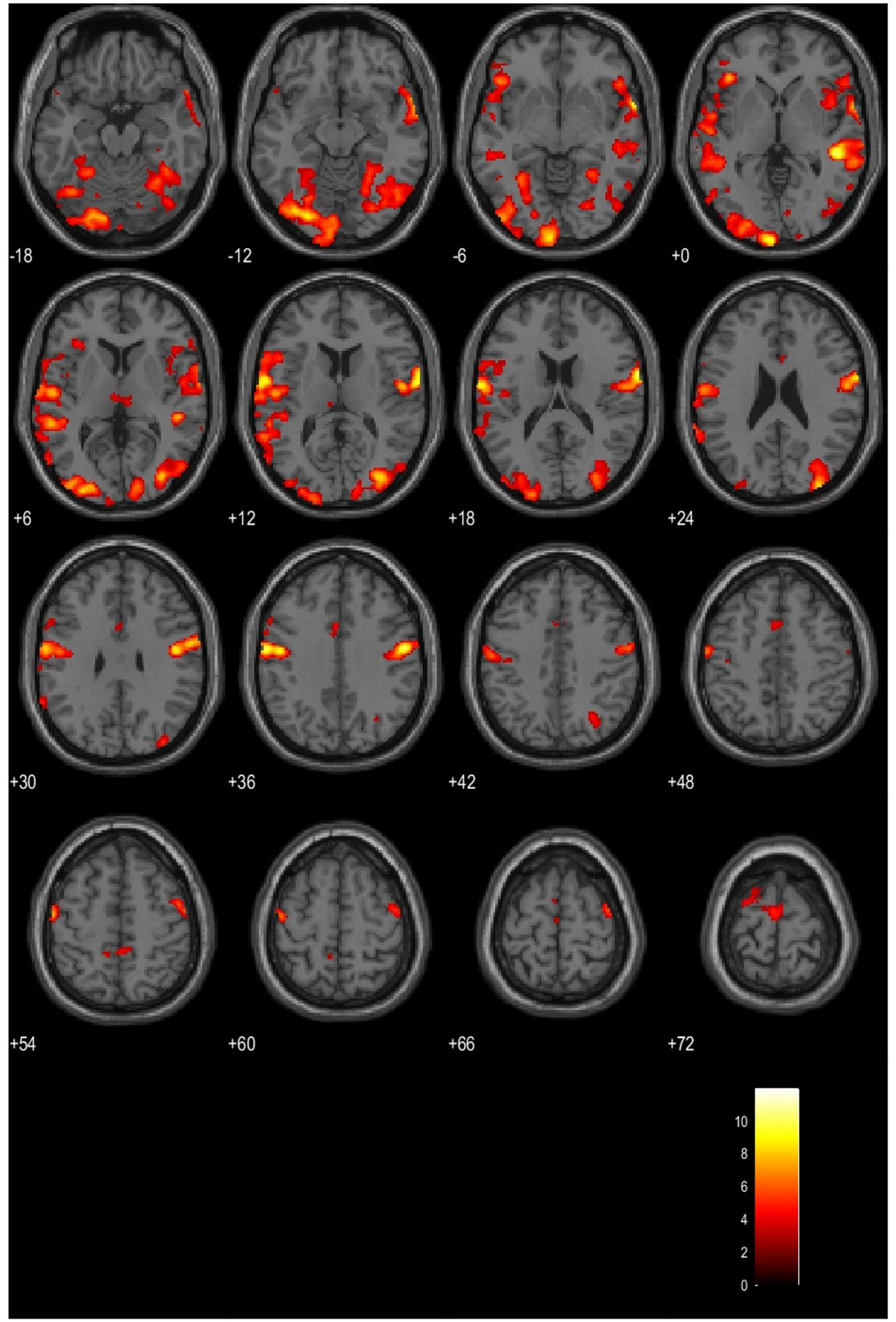

Sentence Completion

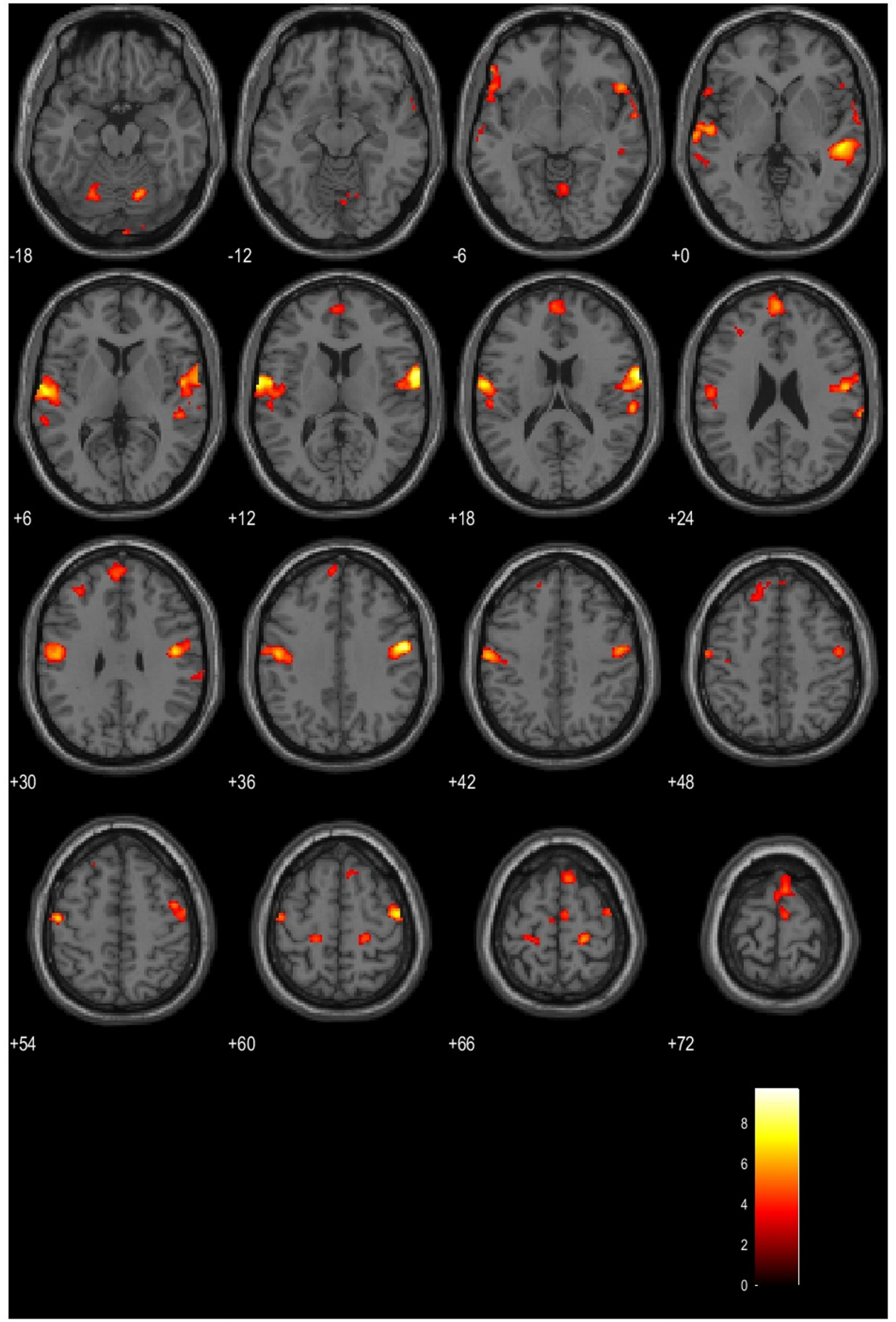

Semantics Association

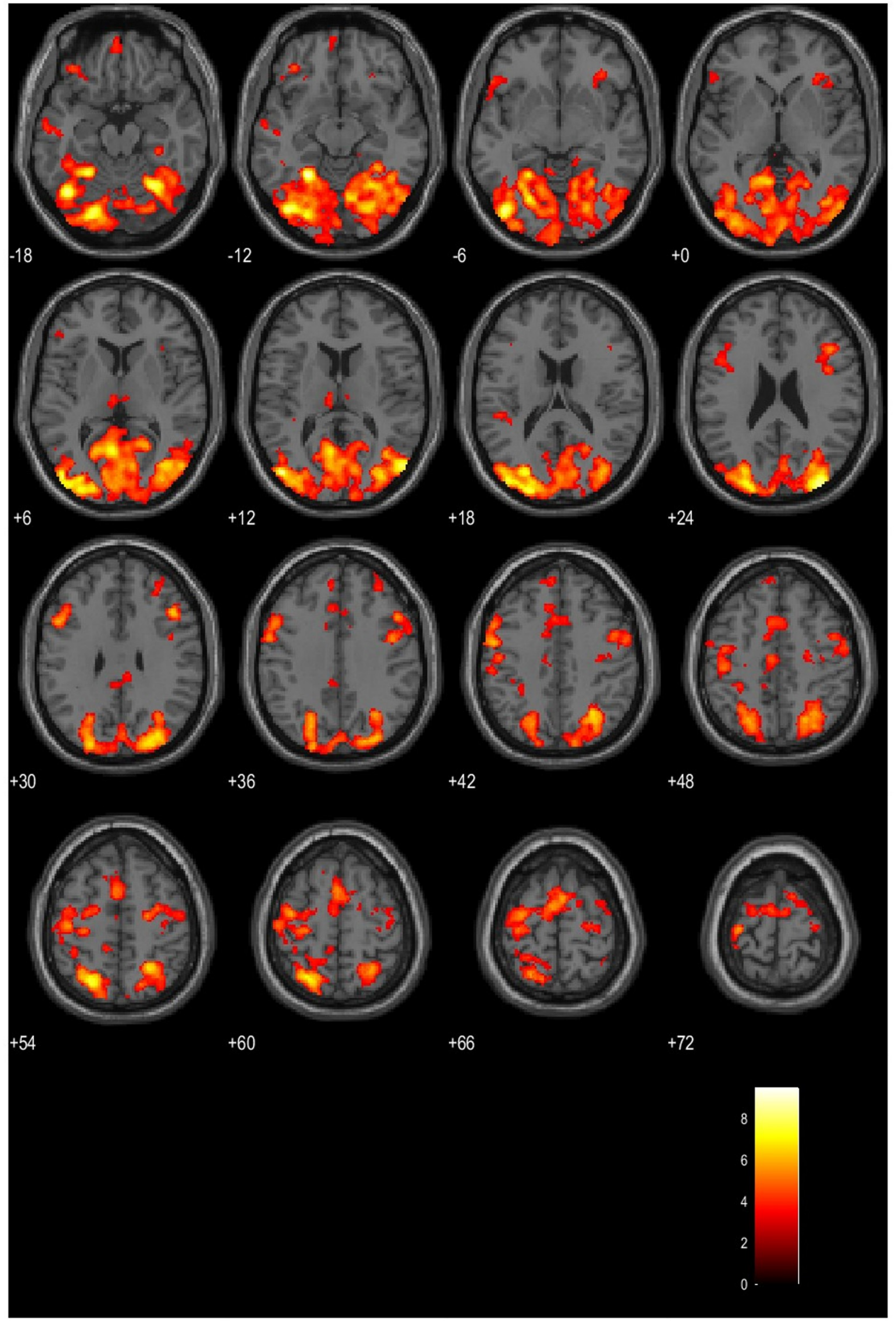

Subject 9

Covert Naming

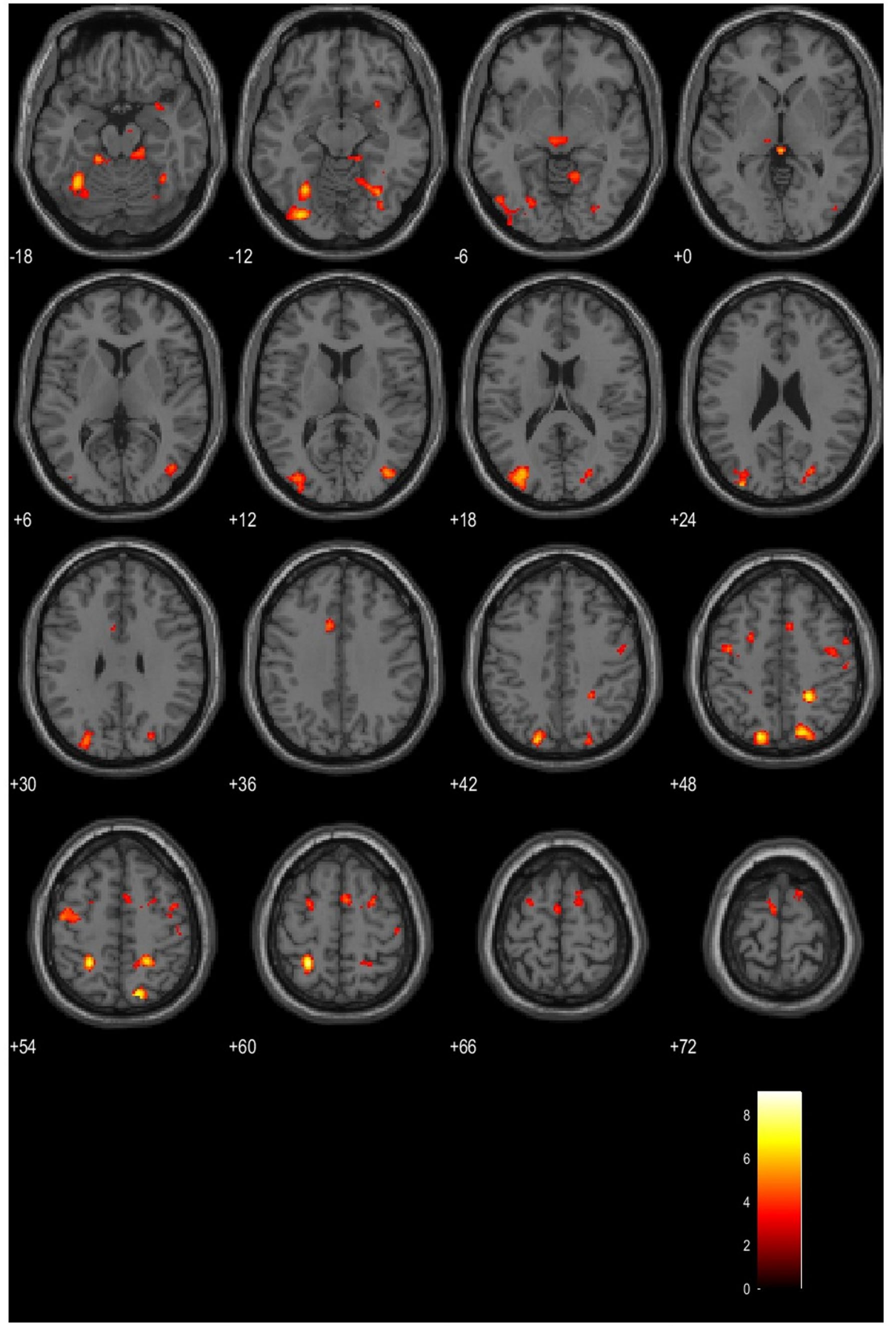

Overt Naming

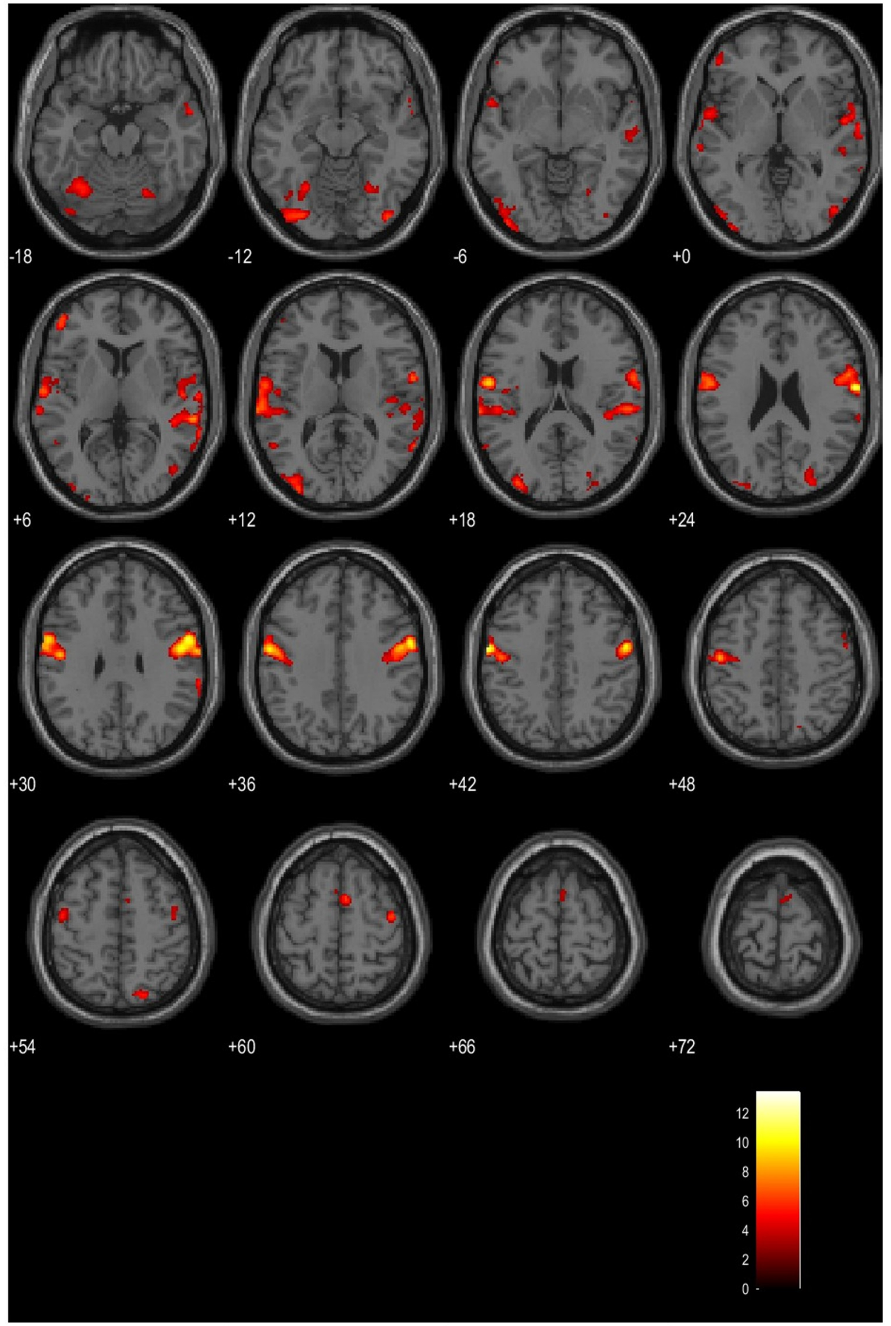

Sentence Completion

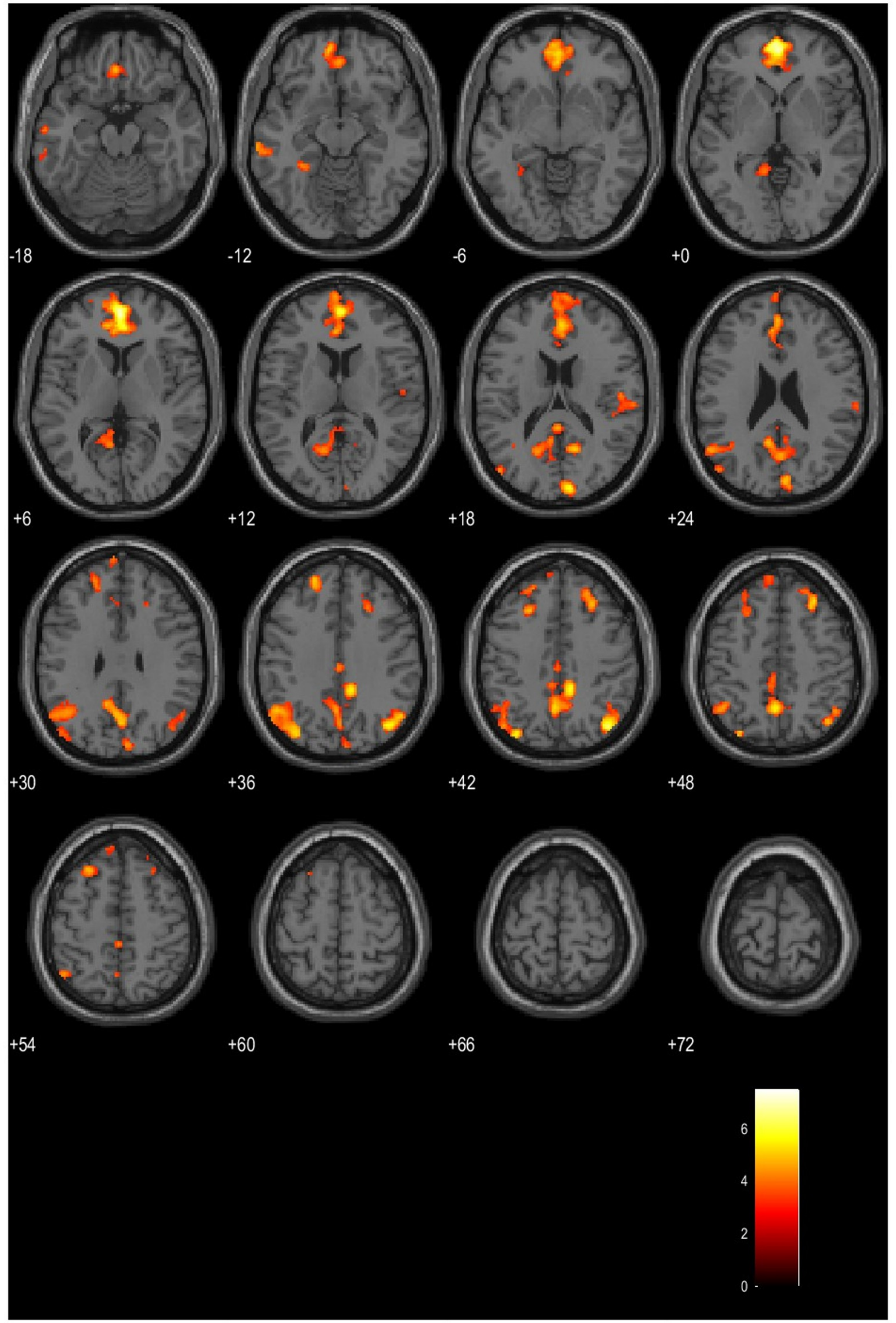

Semantics Association

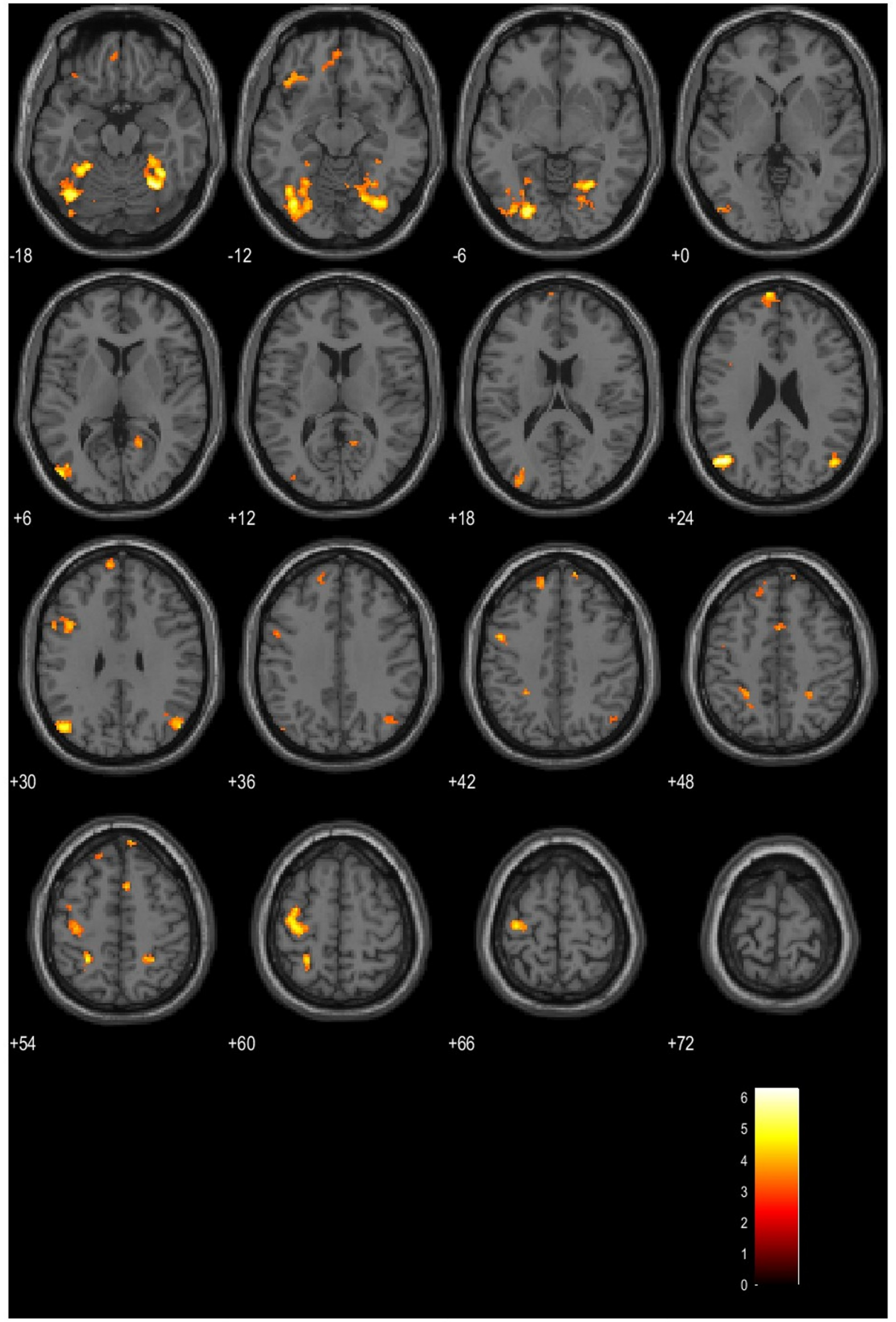

Subject 10

Covert Naming

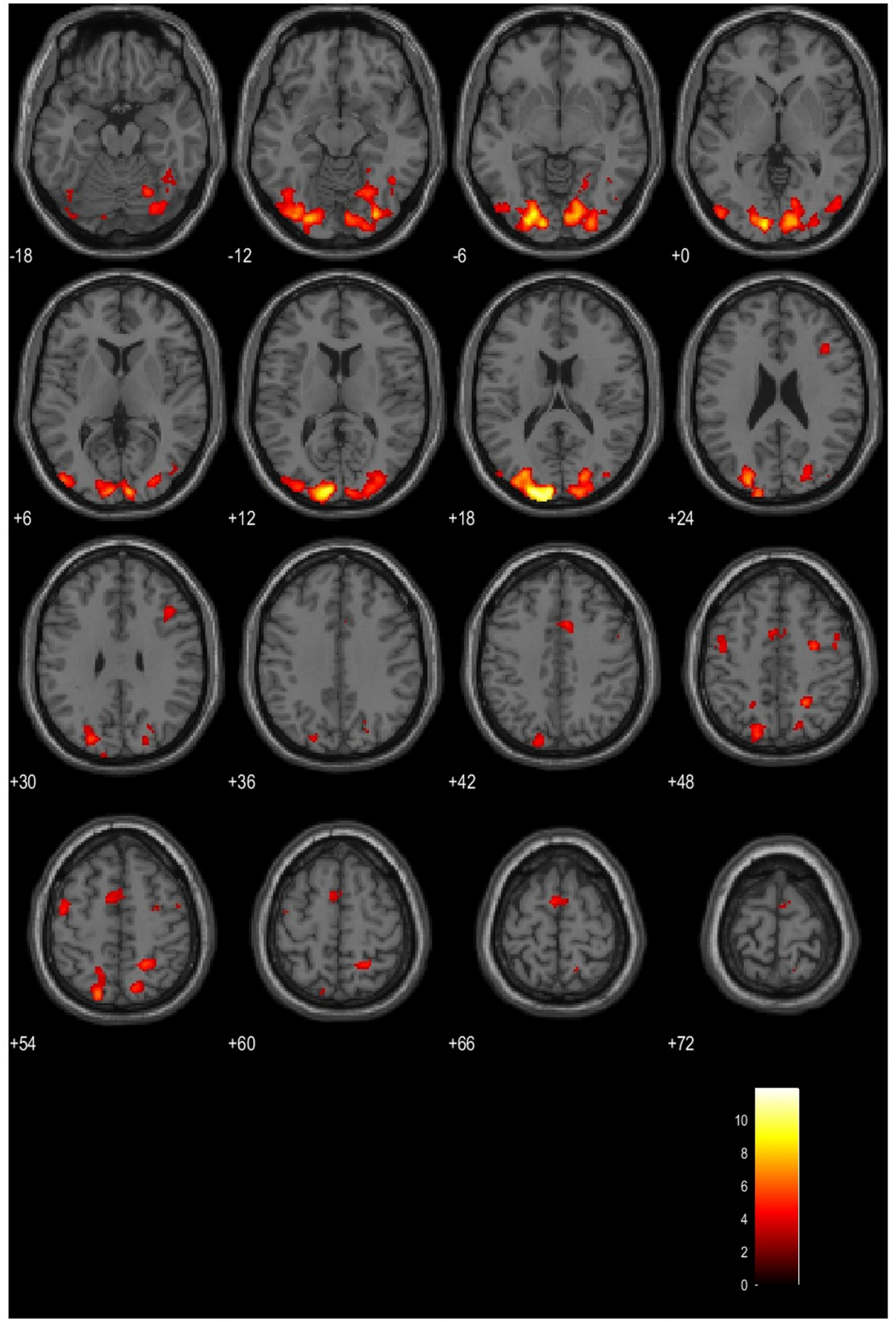

Overt Naming

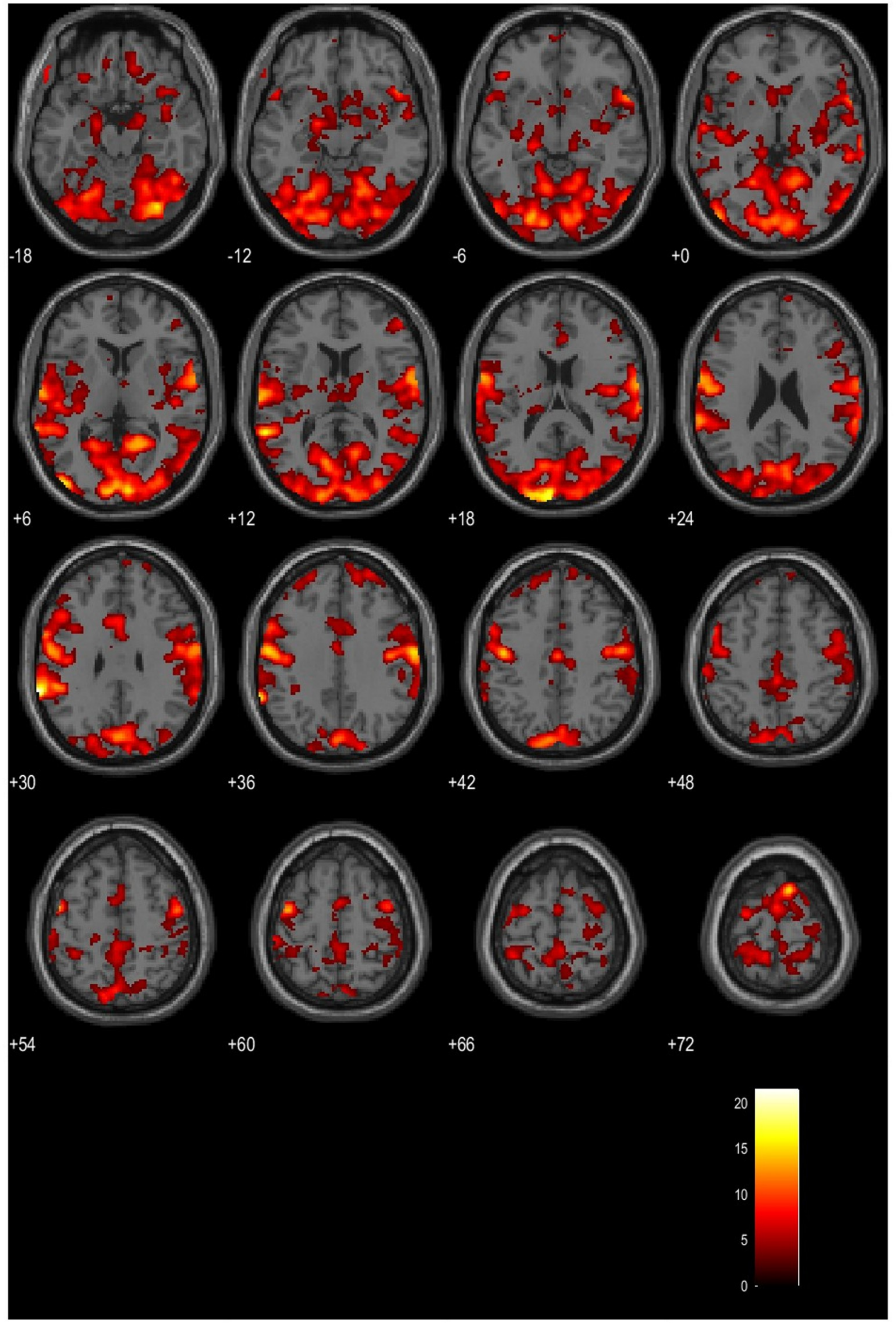

Sentence Completion

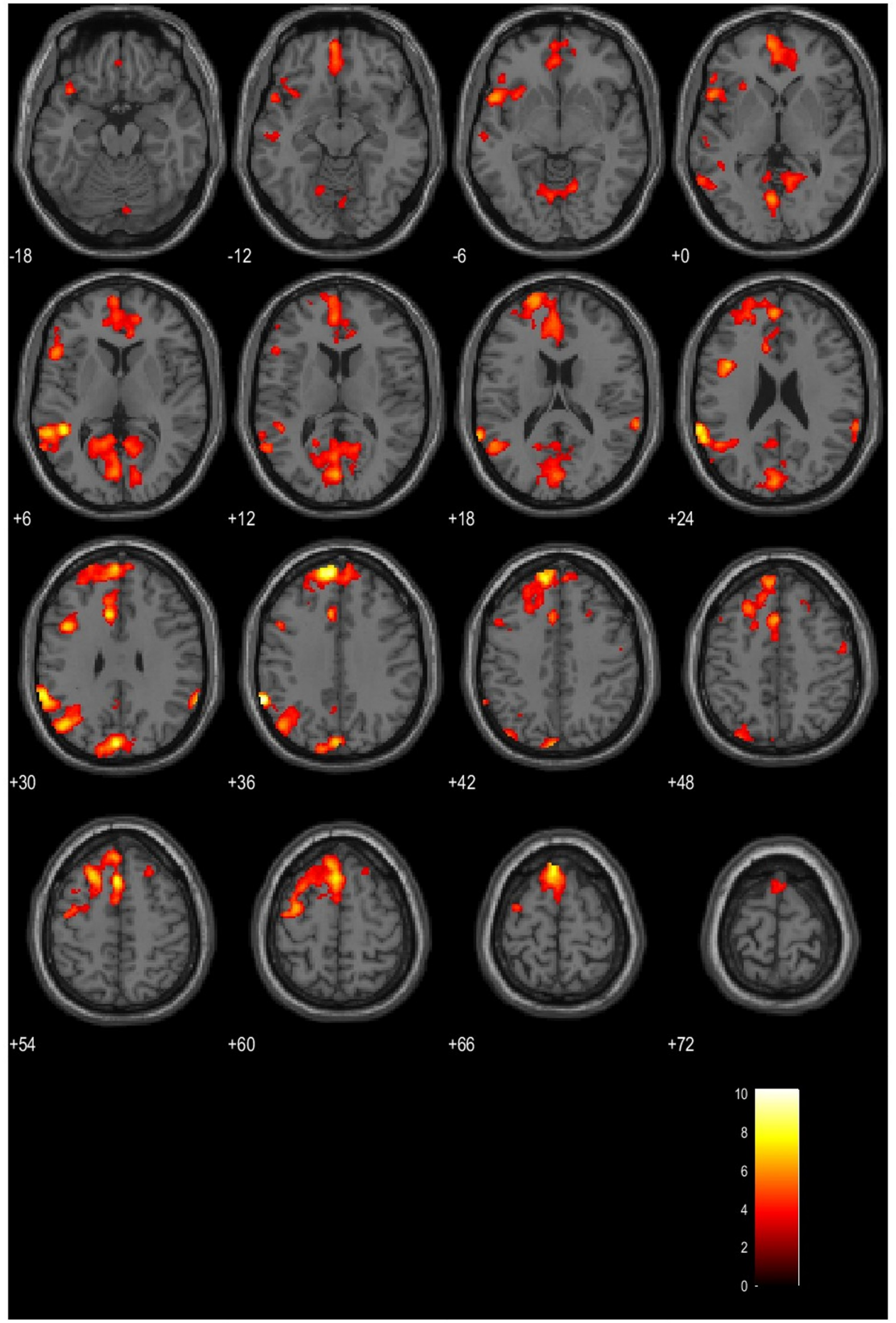

Semantics Association

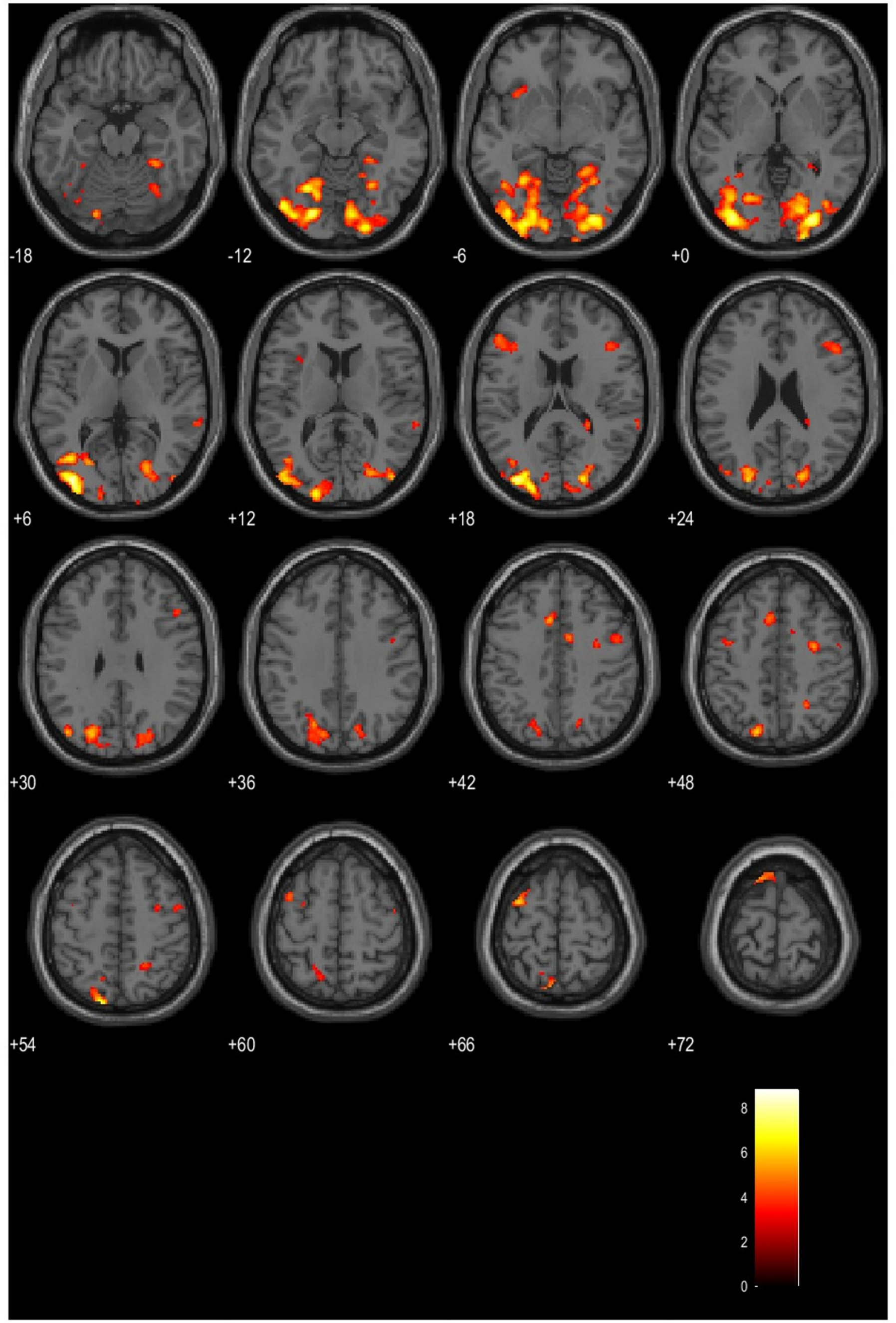

Subject 11

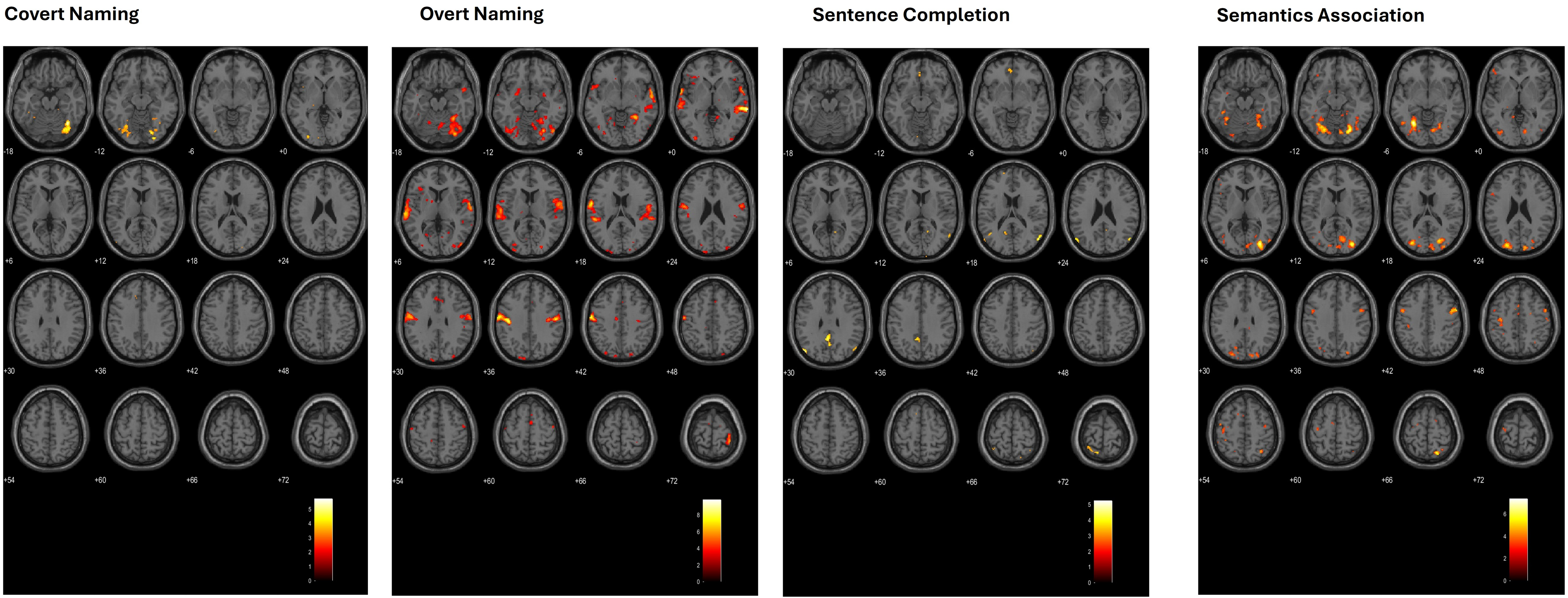
Covert Naming
Overt Naming
Sentence Completion
Semantics Association
-18
-12
-6
+0
+6
+12
+18
+24
+30
+36
+42
+48
+54
+60
+66
+72

Subject 12

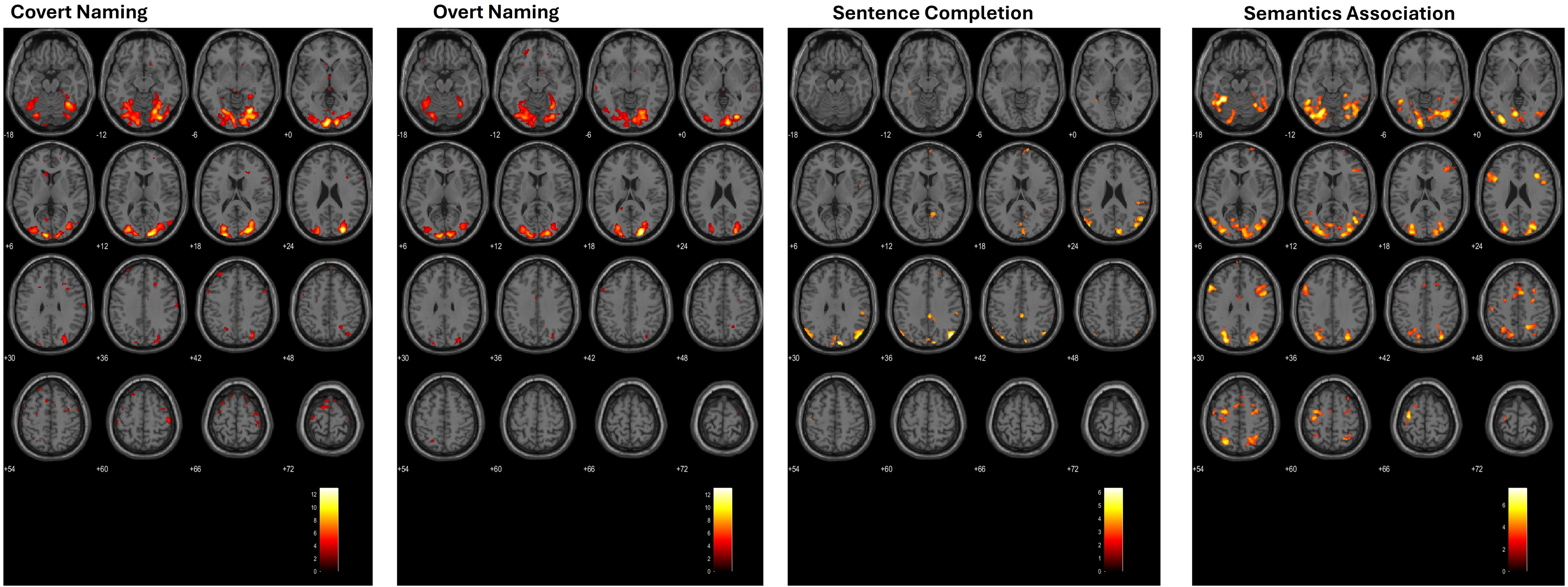

Covert Naming
Overt Naming
Sentence Completion
Semantics Association

Subject 13

Covert Naming

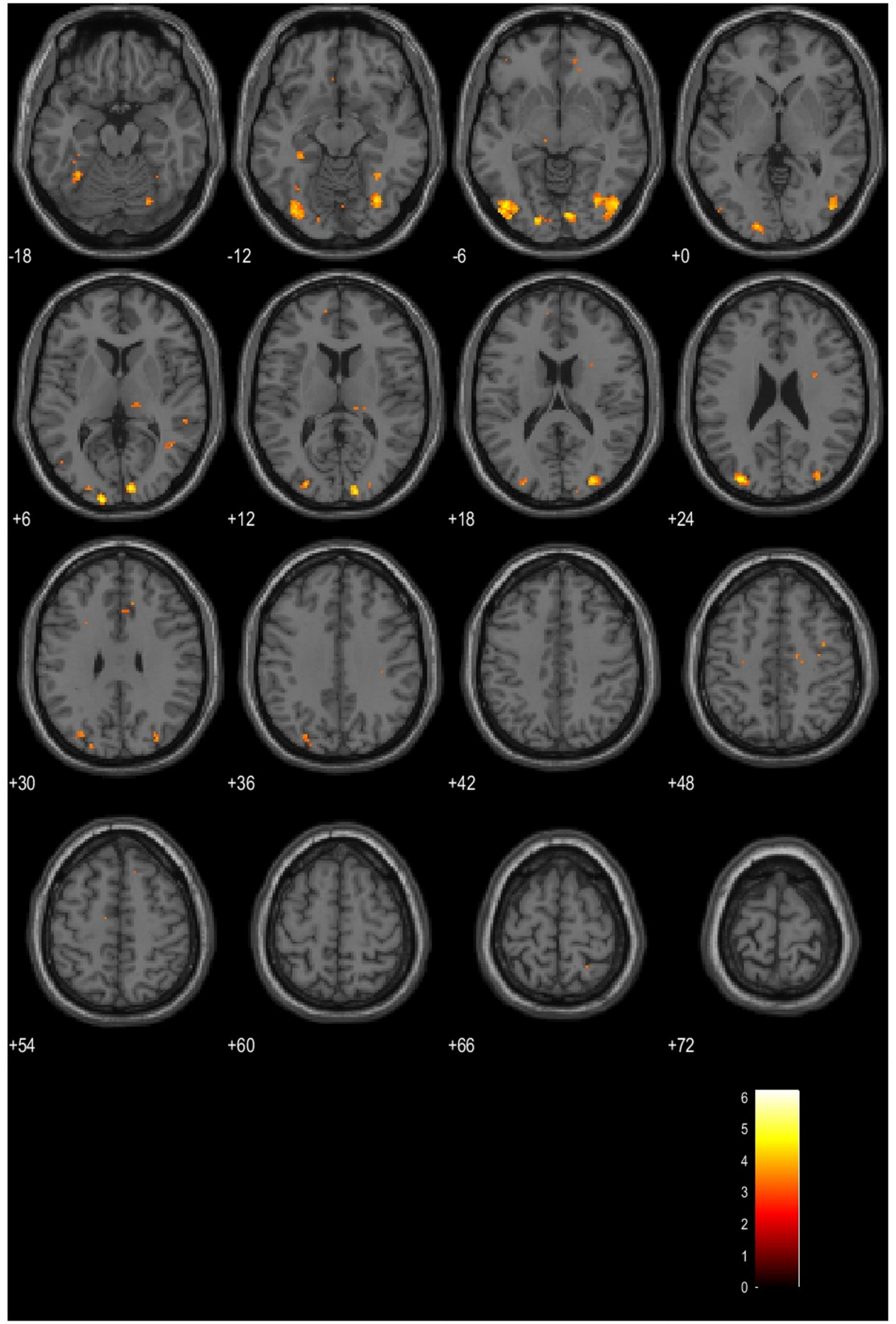

Overt Naming

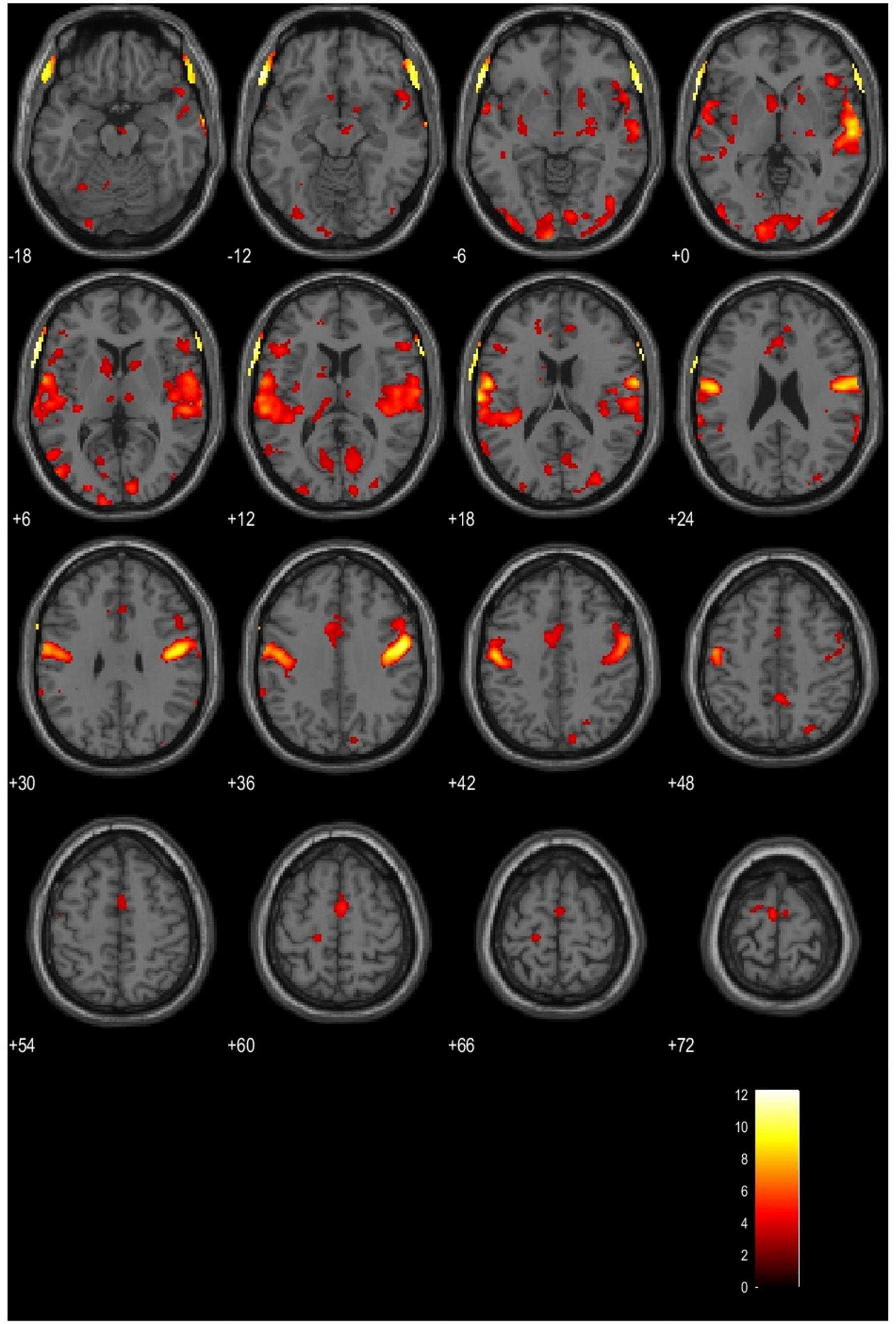

Sentence Completion

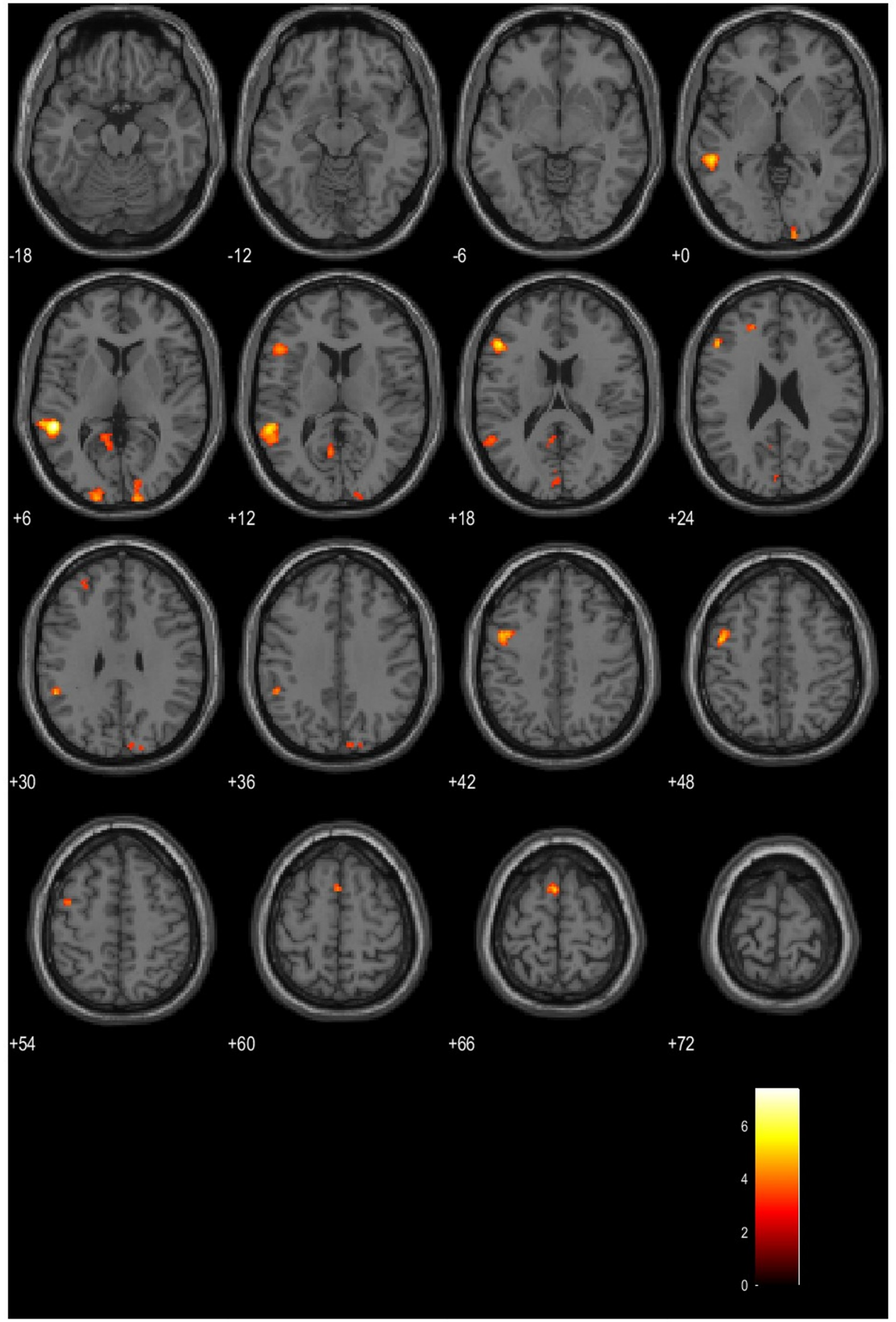

Semantics Association

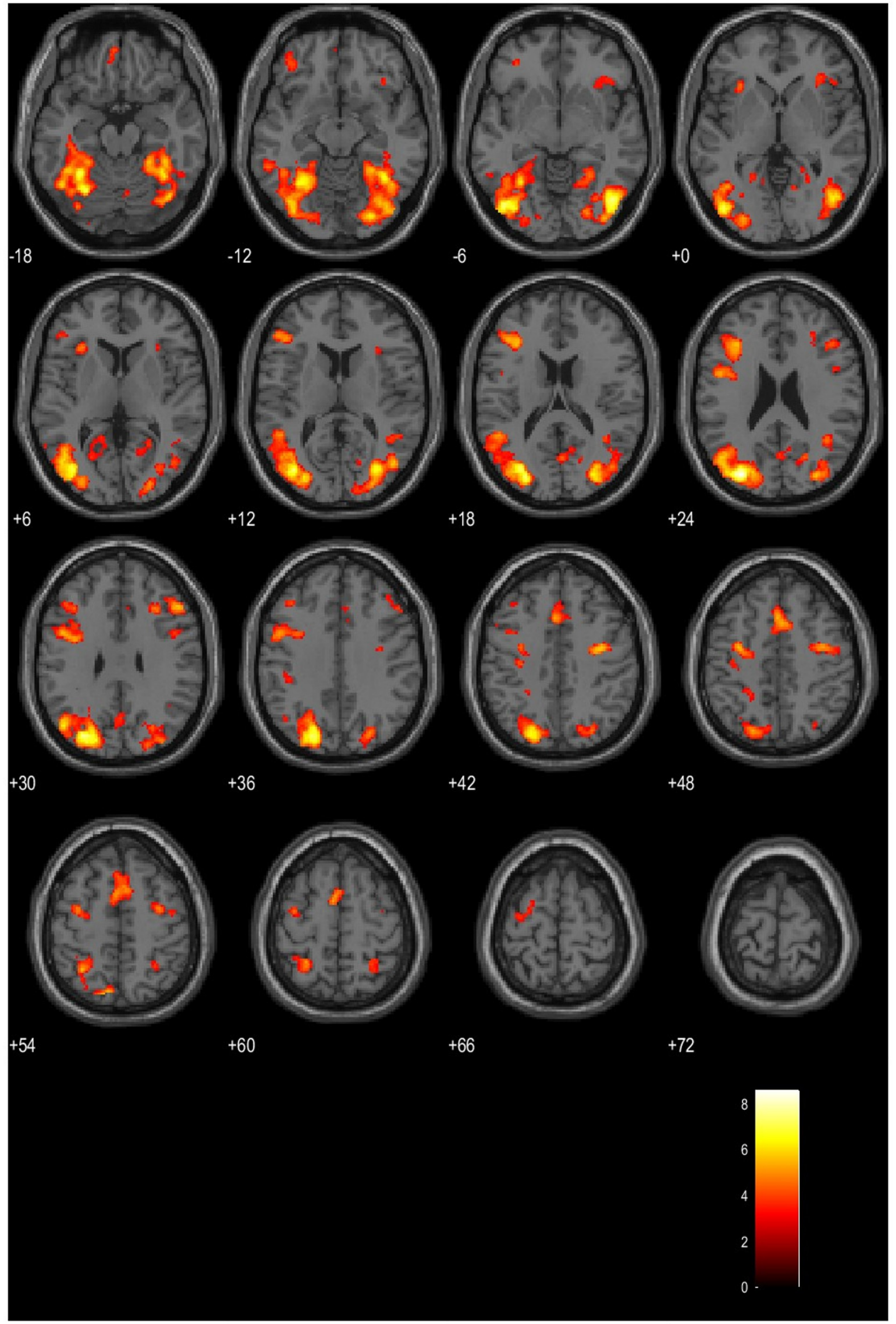

Subject 14

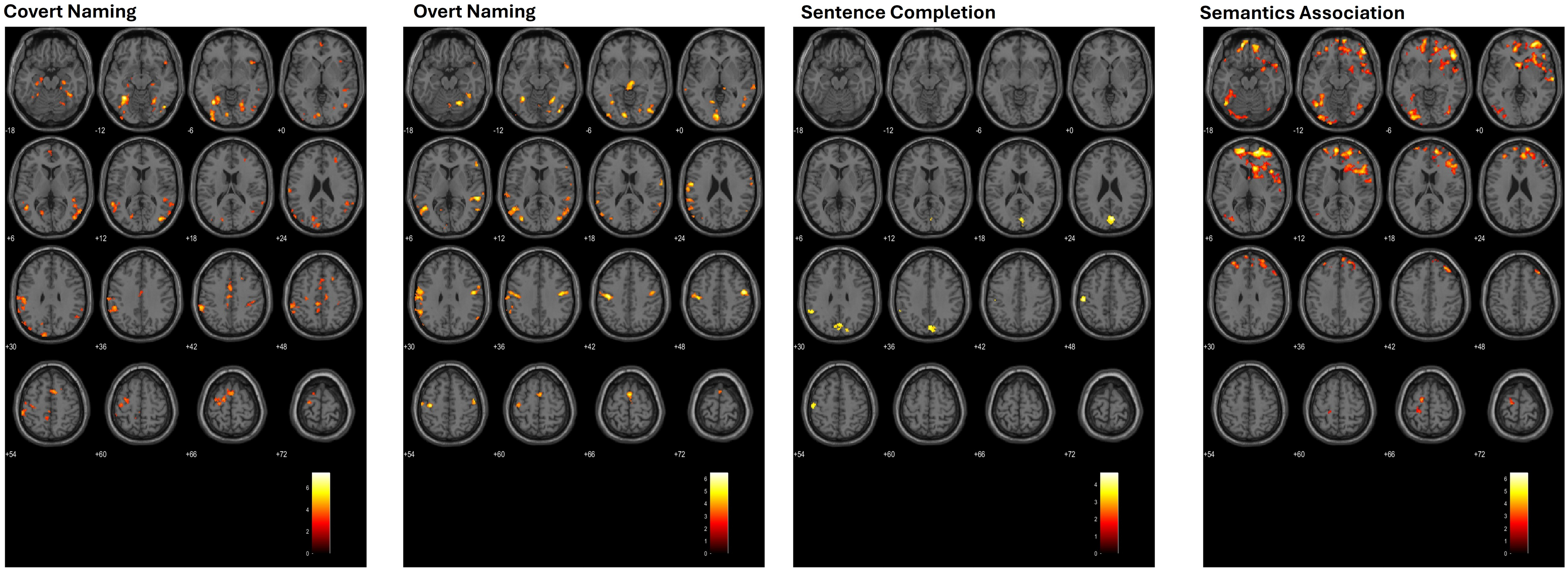
Covert Naming
Overt Naming
Sentence Completion
Semantics Association
-18
-12
-6
+0
+6
+12
+18
+24
+30
+36
+42
+48
+54
+60
+66
+72

Subject 15

Covert Naming | **Overt Naming** | **Sentence Completion** | **Semantics Association**

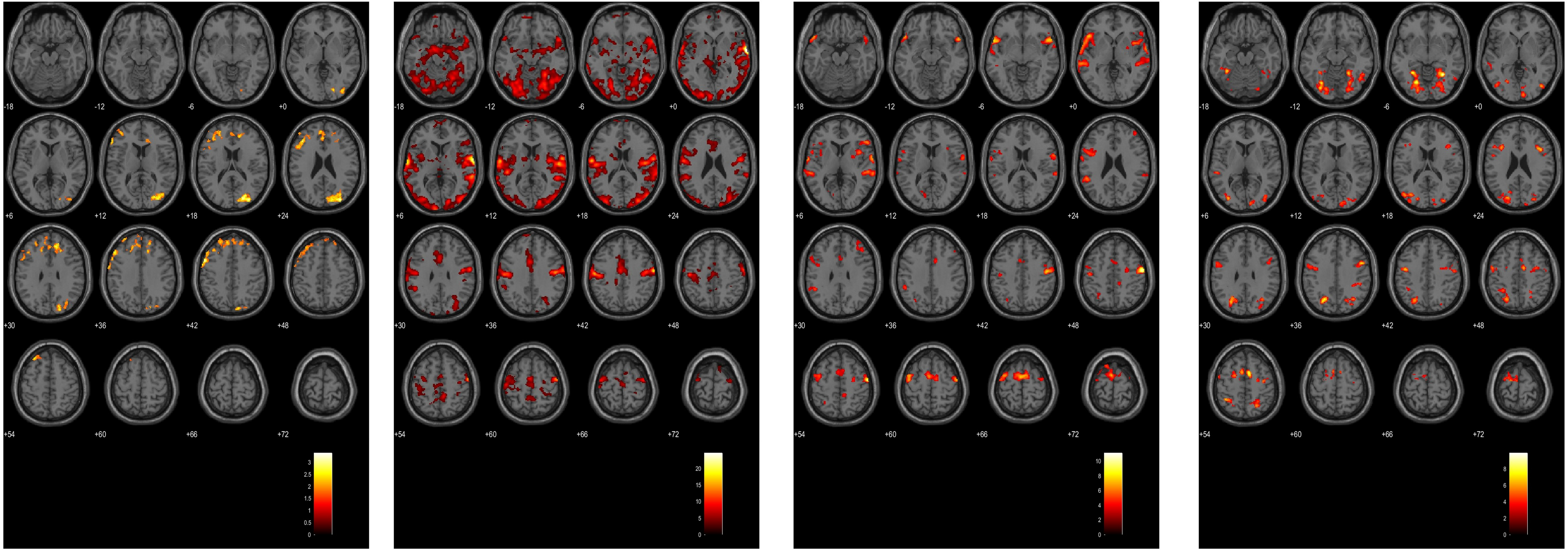

Supplement: Supplementary file 1 — Data S1:Task specific activations for each subject. [file EJN-63-0-s004.pdf]
