# Supplementary material for: A Rapid fMRI Paradigm for Localisation of the Language Network
Source: Eur J Neurosci. 2026 Mar 6;63(5):e70448. doi: 10.1111/ejn.70448 (PMC12964186; doi:10.1111/ejn.70448)
Supplement: Supplementary file 2 — Data S2:Group level activations for each task have been shown on axial slices. Corresponding ROIs have been documented under each task. [file EJN-63-0-s003.pdf]

**SUPPLEMENTARY MATERIAL 2**

**Task 1 Covert Naming**

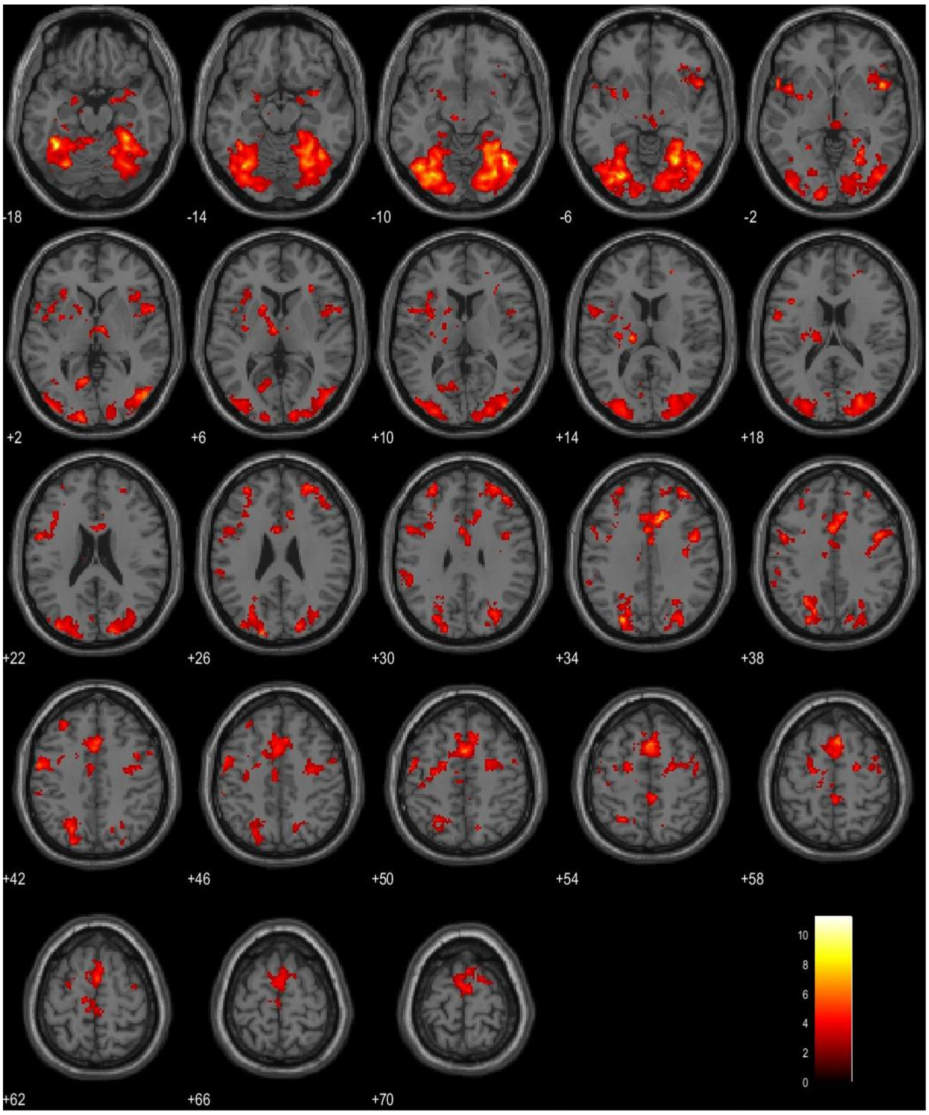


**Significant ROIs**

| MNI | Description |
|---|---|
| 42 -66 -10 | Left thalamus |
| -38 -46 -16 | Right putamen |
| 12 22 36 | Right SMA |
| 48 12 -2 | Right frontal operculum |
| -16 -20 14 | Left thalamus |
| 36 44 36 | Right middle frontal gyrus |
| 46 2 36 | Right precentral gyrus |
| -54 -6 44 | Left precentral gyrus |
| -30 48 32 | Left middle frontal gyrus |
| -6 -10 6 | Left thalamus |
| -20 -4 50 | Left superior frontal gyrus |
| 34 2 -14 | Right insula |
| 52 -40 30 | Left parietal lobule |

**Task 2 Overt Naming**

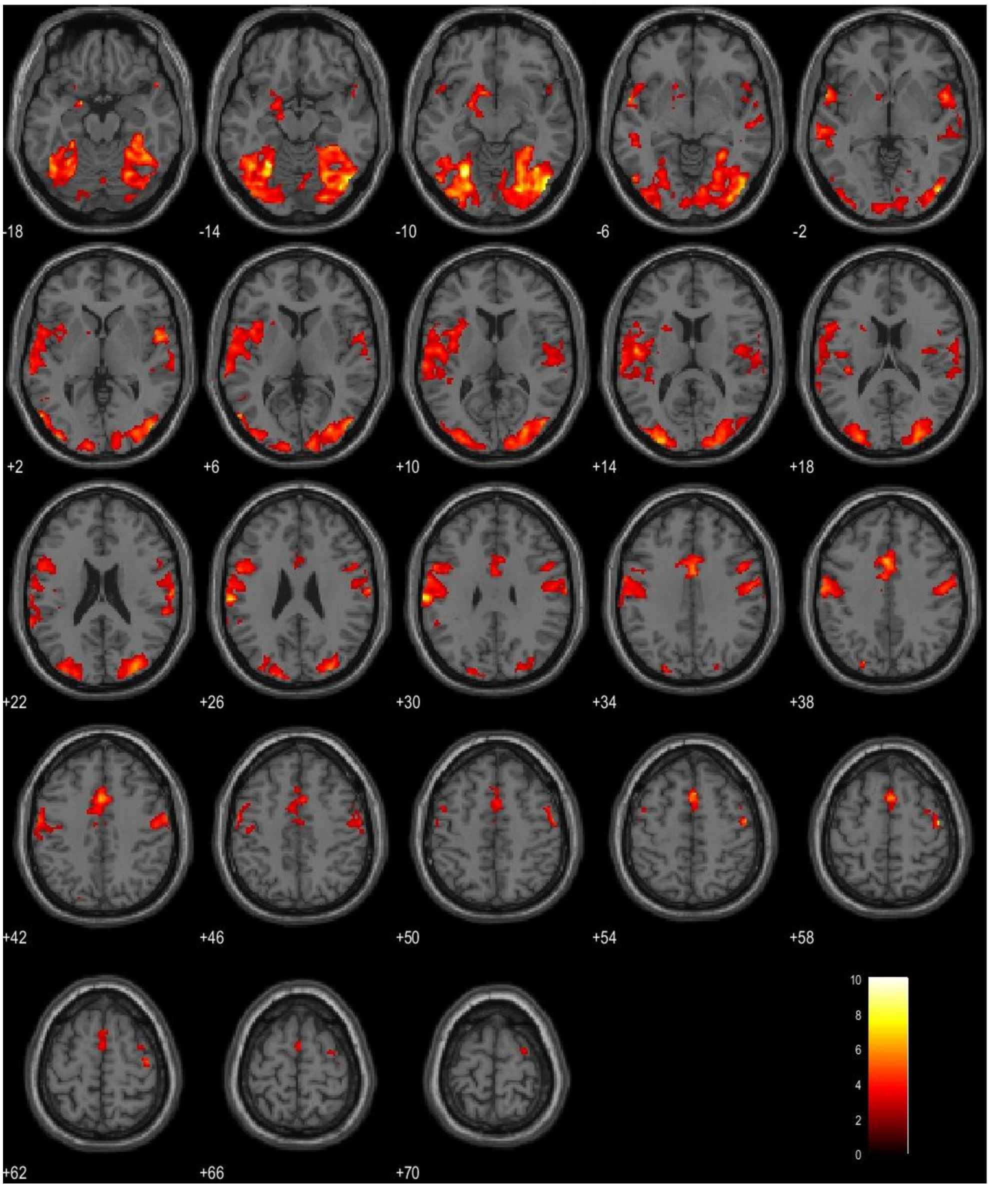


**Significant ROIs**

| MNI | Description |
|---|---|
| 42 -66 -10 | Left fusiform gyrus |
| -38 -46 -16 | Left postcentral gyrus |
| 12 22 36 | Right precentral gyrus |
| 48 12 -2 | Left amygdala |
| -16 -20 14; | Left middle cingulate gyrus |
| 36 44 36 | Right precentral gyrus |

**Task 3 Sentence Completion**

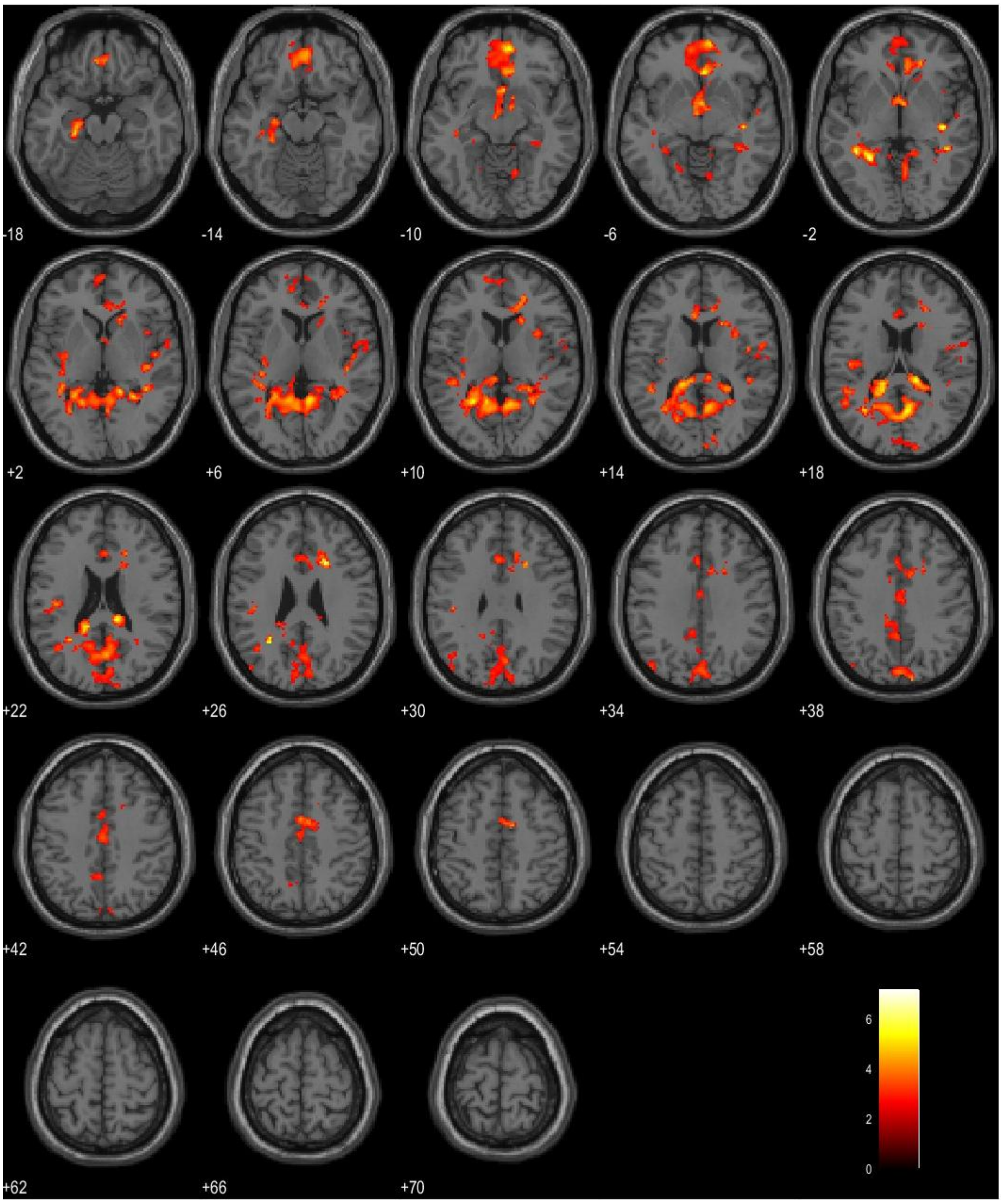


**Significant ROIs**

| MNI | Description |
|---|---|
| -26 -48 -2; | Left lingual gyrus |
| 22 16 26; | Right caudate |
| 42 -20 -4; | Right insula |
| 4 14 -10 | Right acumbens |

**Task 4 Pyramids and Palm Trees Test (PPTT)**

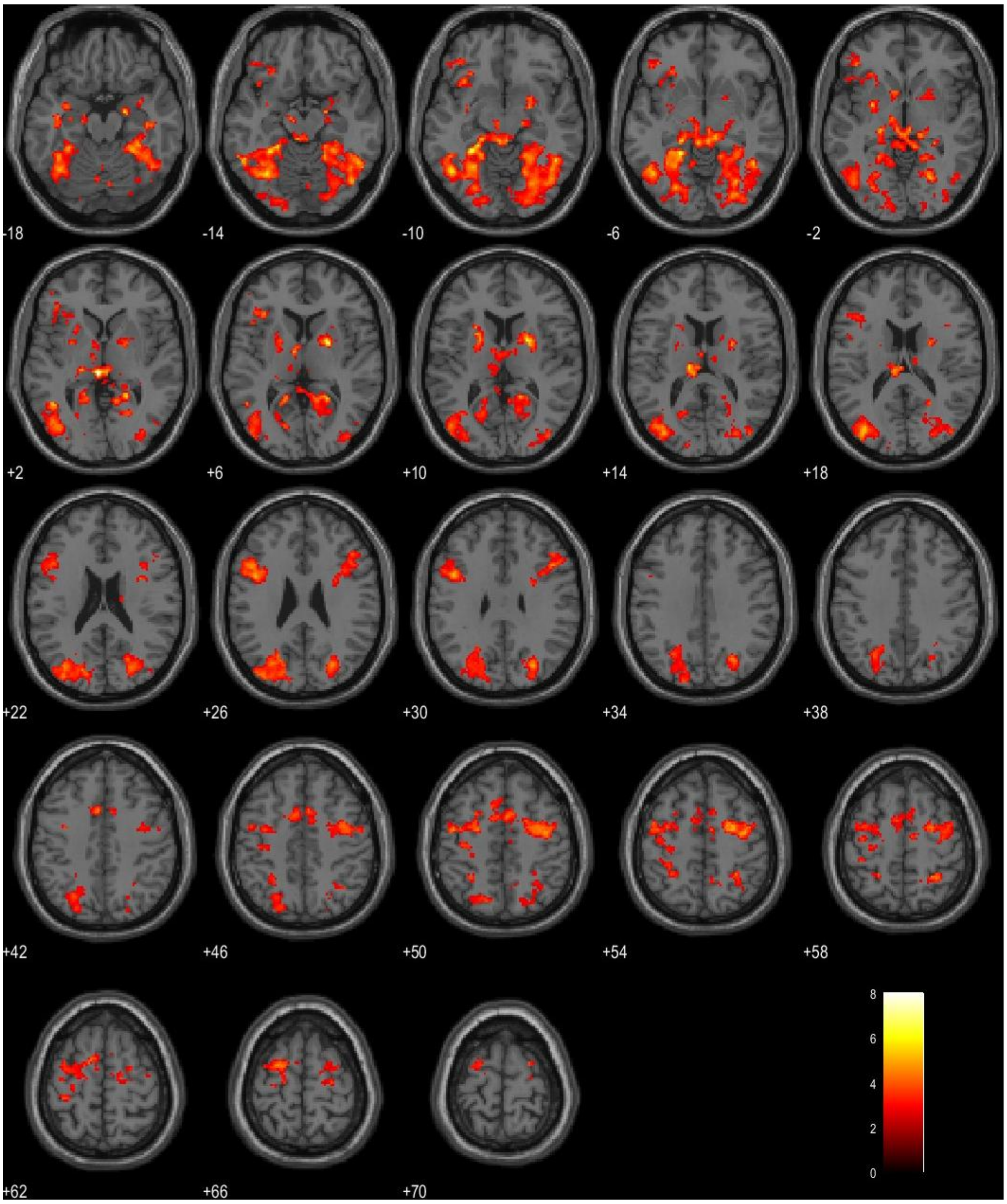


**Significant ROIs**

| MNI | Description |
|---|---|
| -4 -28 2 | Left thalamus |
| 24 0 8 | Right putamen |
| -22 -38 -12 | Left parahippocampal gyrus |
| -40 -14 -20 | Left fusiform gyrus |
| 48 -52 -10 | Right inferior temporal gyrus |
| -12 -22 14 | Right inferior frontal gyrus |
| -42 6 28 | Left caudate |
| -34 -76 20 | Left precentral gyrus |
| 26 -4 56 | Left middle occipital gyrus |
| 28 -74 28 | Right superior frontal gyrus |
