# Supplementary material for: A Rapid fMRI Paradigm for Localisation of the Language Network
Source: Eur J Neurosci. 2026 Mar 6;63(5):e70448. doi: 10.1111/ejn.70448 (PMC12964186; doi:10.1111/ejn.70448)
Supplement: Supplementary file 3 — Data S3:Signal stability for each task for each subject in each task‐level group level ROIs. Signal to noise ratio (SNR) has been documented for each subject for each task. [file EJN-63-0-s001.pdf]

# Supplementary Material 3
# Signal Stability

## Task 1 - Covert Naming

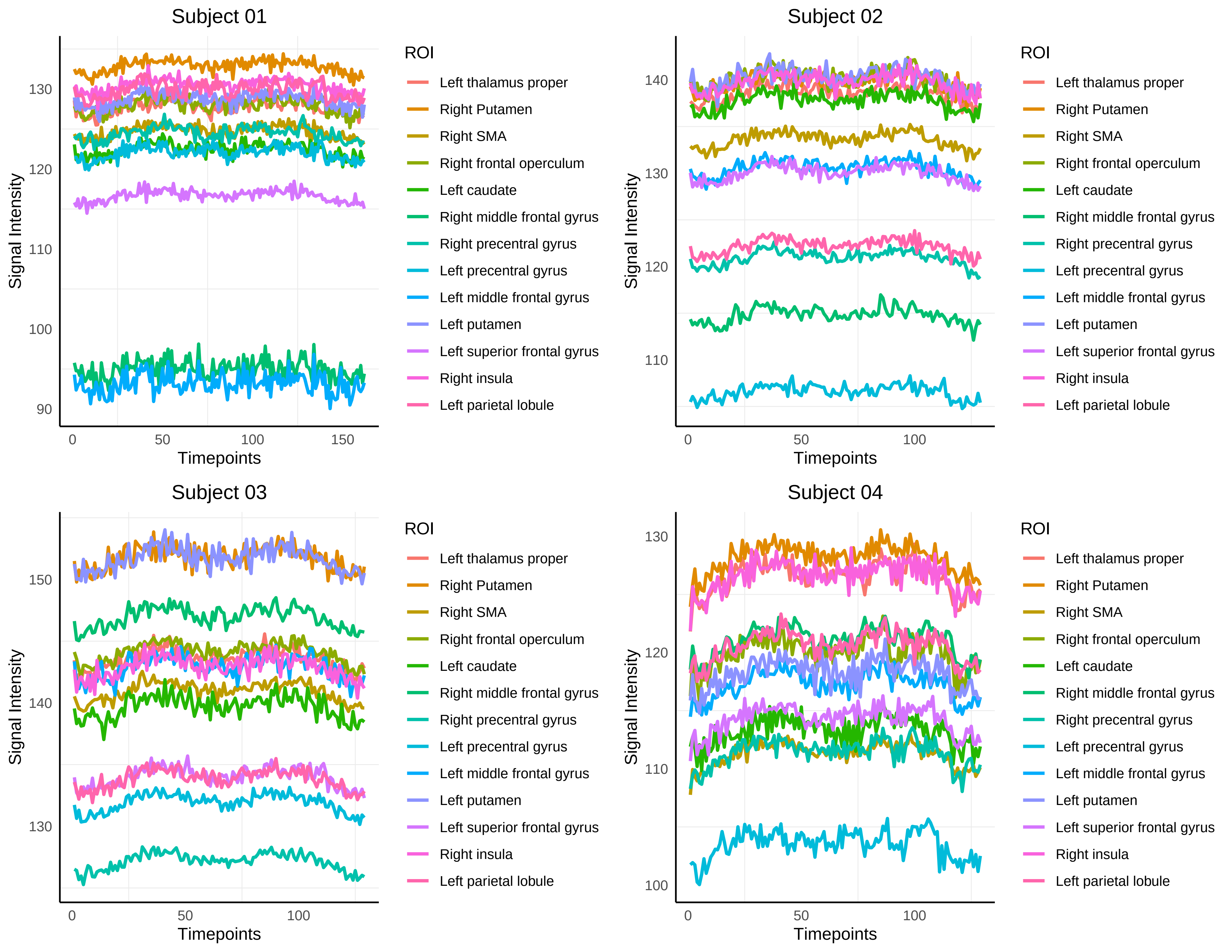
Subject 01
Subject 02
Subject 03
Subject 04
Signal Intensity
Timepoints
ROI
Left thalamus proper
Right Putamen
Right SMA
Right frontal operculum
Left caudate
Right middle frontal gyrus
Right precentral gyrus
Left precentral gyrus
Left middle frontal gyrus
Left putamen
Left superior frontal gyrus
Right insula
Left parietal lobule

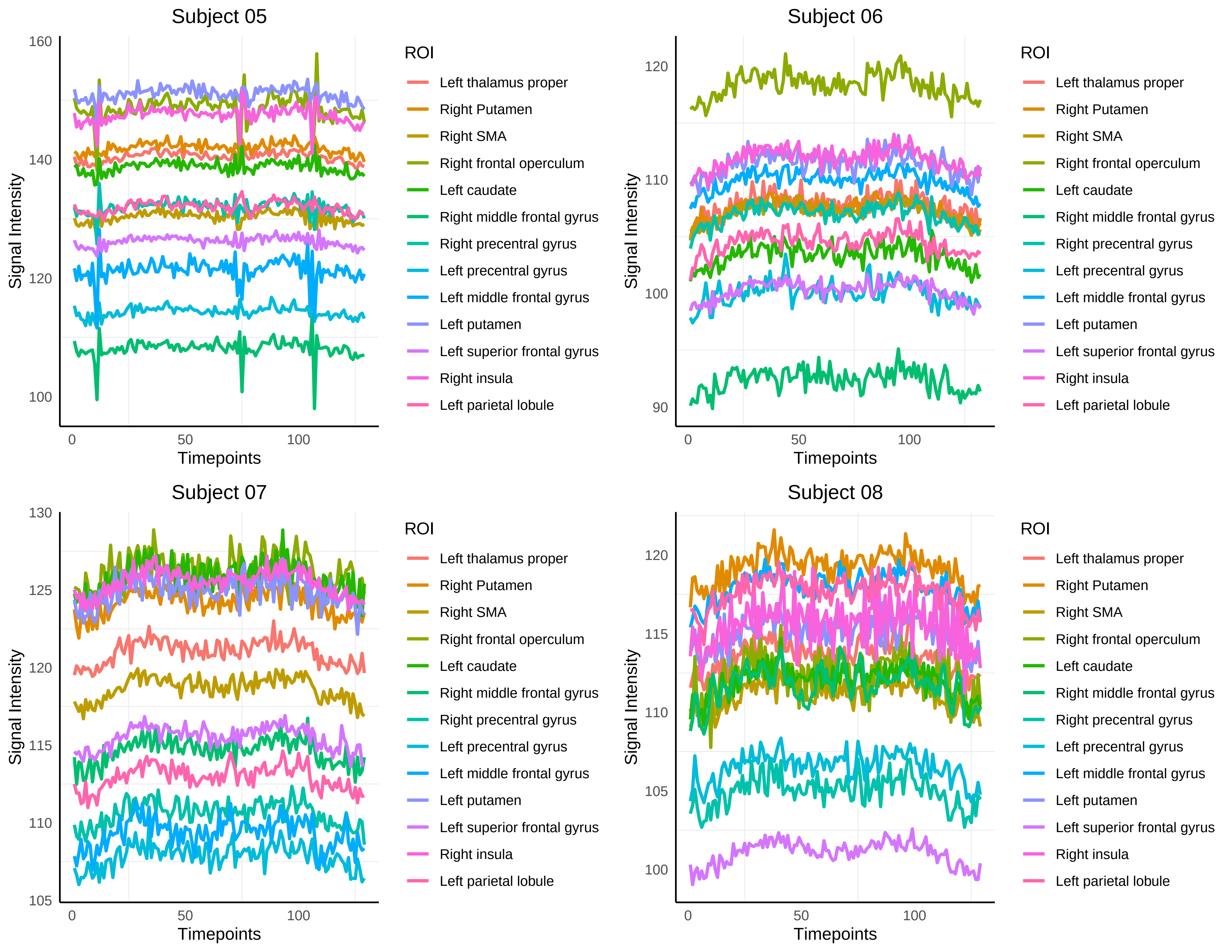
Subject 05
Signal Intensity
Timepoints
160
140
120
100
0
50
100
ROI
Left thalamus proper
Right Putamen
Right SMA
Right frontal operculum
Left caudate
Right middle frontal gyrus
Right precentral gyrus
Left precentral gyrus
Left middle frontal gyrus
Left putamen
Left superior frontal gyrus
Right insula
Left parietal lobule
Subject 06
Signal Intensity
Timepoints
120
110
100
90
0
50
100
ROI
Left thalamus proper
Right Putamen
Right SMA
Right frontal operculum
Left caudate
Right middle frontal gyrus
Right precentral gyrus
Left precentral gyrus
Left middle frontal gyrus
Left putamen
Left superior frontal gyrus
Right insula
Left parietal lobule
Subject 07
Signal Intensity
Timepoints
130
125
120
115
110
105
0
50
100
ROI
Left thalamus proper
Right Putamen
Right SMA
Right frontal operculum
Left caudate
Right middle frontal gyrus
Right precentral gyrus
Left precentral gyrus
Left middle frontal gyrus
Left putamen
Left superior frontal gyrus
Right insula
Left parietal lobule
Subject 08
Signal Intensity
Timepoints
120
115
110
105
100
0
50
100
ROI
Left thalamus proper
Right Putamen
Right SMA
Right frontal operculum
Left caudate
Right middle frontal gyrus
Right precentral gyrus
Left precentral gyrus
Left middle frontal gyrus
Left putamen
Left superior frontal gyrus
Right insula
Left parietal lobule

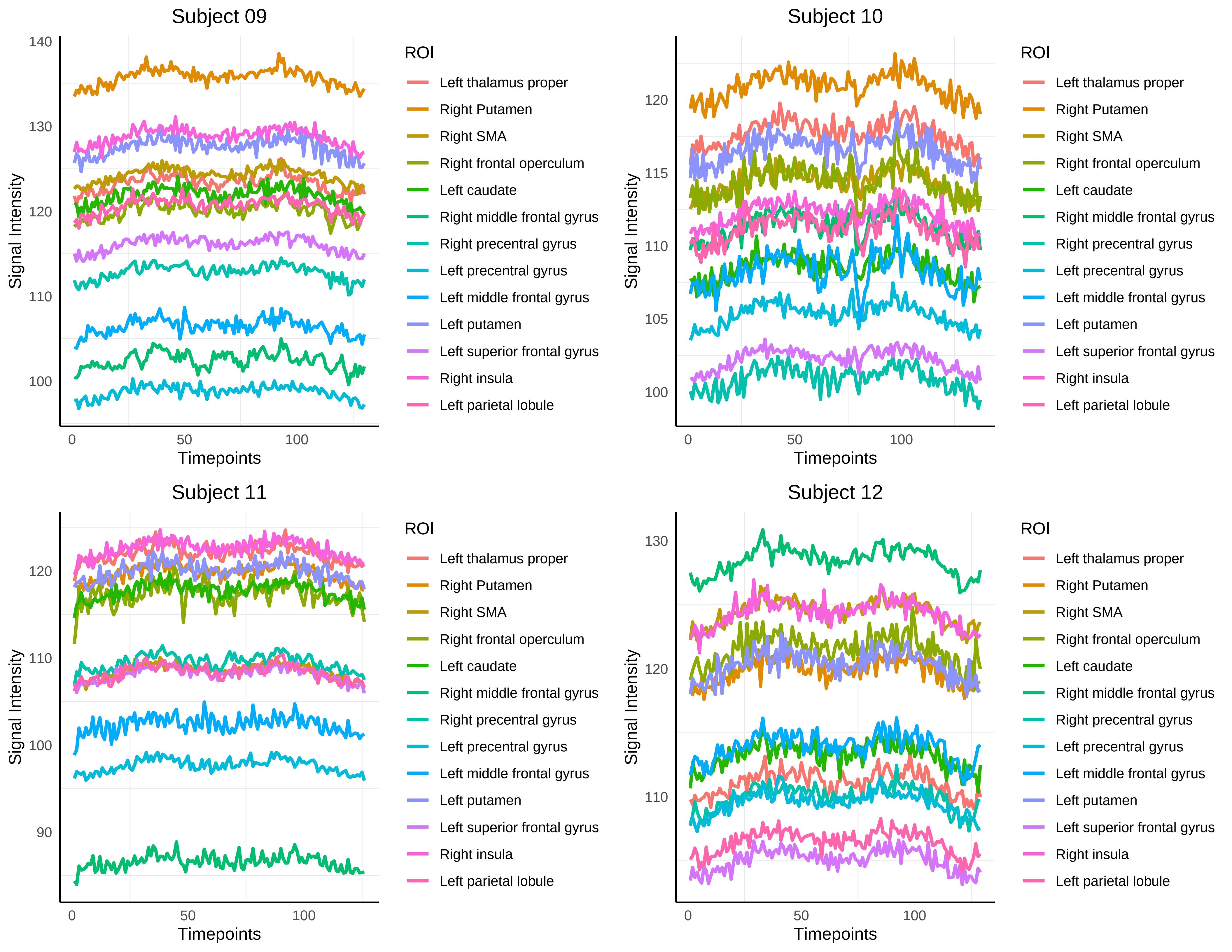
Subject 09
Subject 10
Subject 11
Subject 12
Signal Intensity
Timepoints
140
130
120
110
100
0
50
100
115
105
90
ROI
Left thalamus proper
Right Putamen
Right SMA
Right frontal operculum
Left caudate
Right middle frontal gyrus
Right precentral gyrus
Left precentral gyrus
Left middle frontal gyrus
Left putamen
Left superior frontal gyrus
Right insula
Left parietal lobule

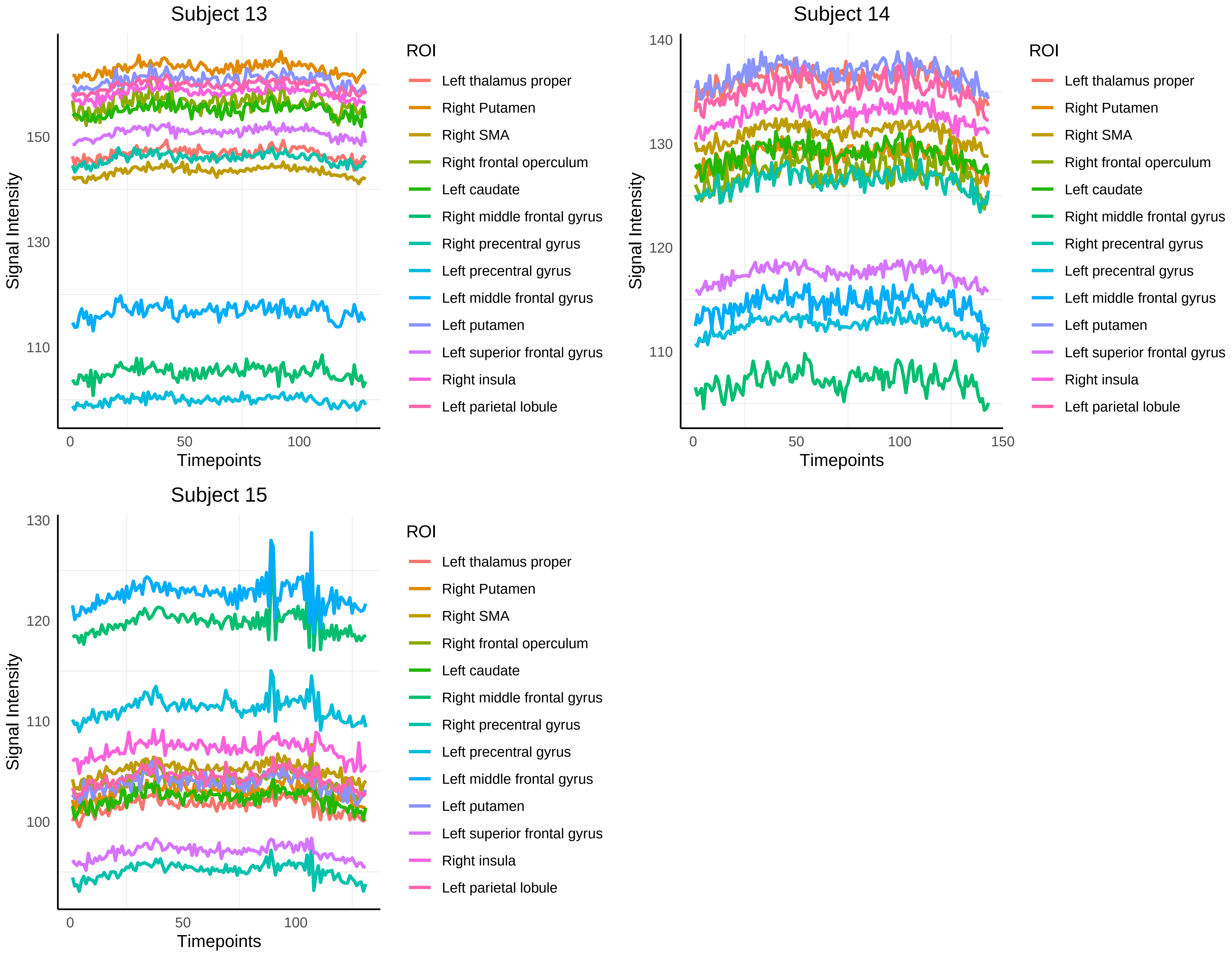
Subject 13
Signal Intensity
150
130
110
0
50
100
Timepoints
ROI
Left thalamus proper
Right Putamen
Right SMA
Right frontal operculum
Left caudate
Right middle frontal gyrus
Right precentral gyrus
Left precentral gyrus
Left middle frontal gyrus
Left putamen
Left superior frontal gyrus
Right insula
Left parietal lobule
Subject 14
Signal Intensity
140
130
120
110
0
50
100
150
Timepoints
ROI
Left thalamus proper
Right Putamen
Right SMA
Right frontal operculum
Left caudate
Right middle frontal gyrus
Right precentral gyrus
Left precentral gyrus
Left middle frontal gyrus
Left putamen
Left superior frontal gyrus
Right insula
Left parietal lobule
Subject 15
Signal Intensity
130
120
110
100
0
50
100
Timepoints
ROI
Left thalamus proper
Right Putamen
Right SMA
Right frontal operculum
Left caudate
Right middle frontal gyrus
Right precentral gyrus
Left precentral gyrus
Left middle frontal gyrus
Left putamen
Left superior frontal gyrus
Right insula
Left parietal lobule

# Temporal Signal to Noise Ratio (tSNR)

| Subject | Left thalamus | Right Putamen | Right SMA | Right frontal operculum | Left thalamus | Right middle frontal gyrus | Right precentral gyrus | Left precentral gyrus | Left middle frontal gyrus | Left thalamus | Left superior frontal gyrus | Right insula | Left parietal lobule |
|---|---|---|---|---|---|---|---|---|---|---|---|---|---|
| sub_01 | 147.13169 | 159.7968 | 154.8742 | 134.96665 | 145.0449 | 79.37012 | 146.29067 | 141.44068 | 73.44673 | 139.65018 | 160.34450 | 153.29072 | 130.6615 |
| sub_02 | 154.63995 | 148.0158 | 157.4133 | 133.81485 | 144.5328 | 137.81833 | 154.57032 | 140.73139 | 144.86326 | 132.83710 | 154.24725 | 144.25872 | 151.3862 |
| sub_03 | 173.25680 | 156.0404 | 184.7667 | 178.06897 | 149.3703 | 174.96204 | 183.92375 | 180.37193 | 154.23235 | 155.23943 | 168.09131 | 160.33910 | 174.5487 |
| sub_04 | 97.11613 | 104.2083 | 112.7752 | 85.63974 | 92.7388 | 99.15473 | 97.69093 | 86.05984 | 100.00510 | 93.26012 | 96.75766 | 94.89942 | 97.6079 |
| sub_05 | 168.48000 | 153.5975 | 122.8523 | 65.26472 | 126.9689 | 62.29897 | 103.37812 | 121.09508 | 61.18780 | 126.43144 | 144.72701 | 101.27266 | 136.3202 |
| sub_06 | 105.54855 | 122.0910 | 127.2060 | 104.76077 | 104.7374 | 92.03634 | 118.56020 | 95.30564 | 121.02085 | 109.32843 | 119.92125 | 113.79296 | 109.4191 |
| sub_07 | 155.12295 | 148.6784 | 154.9729 | 110.18581 | 127.6196 | 137.60991 | 138.63420 | 137.63663 | 114.16254 | 128.83919 | 154.99896 | 154.02266 | 145.9284 |
| sub_08 | 119.04899 | 117.0264 | 124.0188 | 78.05820 | 113.3215 | 93.32551 | 104.90921 | 115.12537 | 121.34541 | 108.01065 | 132.68424 | 73.63217 | 116.7664 |
| sub_09 | 120.64287 | 135.4546 | 142.0242 | 119.13487 | 119.1043 | 102.46356 | 127.05123 | 131.45219 | 109.18947 | 114.04047 | 132.99789 | 129.04712 | 131.5744 |
| sub_10 | 126.39130 | 129.5696 | 130.7194 | 95.73147 | 120.6009 | 113.65674 | 121.01385 | 132.50297 | 90.85104 | 117.22342 | 141.84667 | 132.45019 | 113.1988 |
| sub_11 | 119.96077 | 129.6433 | 135.1137 | 85.17964 | 118.9527 | 100.37864 | 120.48707 | 123.75858 | 98.91926 | 112.85807 | 131.09342 | 119.24853 | 122.7518 |
| sub_12 | 117.72230 | 117.3845 | 126.4518 | 98.39531 | 113.0285 | 123.00369 | 117.37729 | 123.79149 | 105.77654 | 109.36493 | 121.72173 | 117.03028 | 118.3228 |
| sub_13 | 142.64039 | 146.4087 | 159.6165 | 94.40454 | 130.6410 | 84.02935 | 145.92618 | 125.94945 | 87.68924 | 131.90396 | 152.93287 | 152.79563 | 157.7083 |
| sub_14 | 128.81748 | 127.1902 | 147.0796 | 105.85901 | 124.1291 | 100.05642 | 124.39373 | 143.16589 | 110.83813 | 127.23955 | 143.25009 | 139.95769 | 123.9249 |
| sub_15 | 132.00877 | 128.2295 | 123.7329 | 119.87269 | 122.0248 | 89.20490 | 122.17882 | 103.87874 | 87.51534 | 115.36886 | 141.46330 | 122.73789 | 123.2520 |

# Supplementary Material 3
# Signal Stability

## Task 2 - Overt Naming

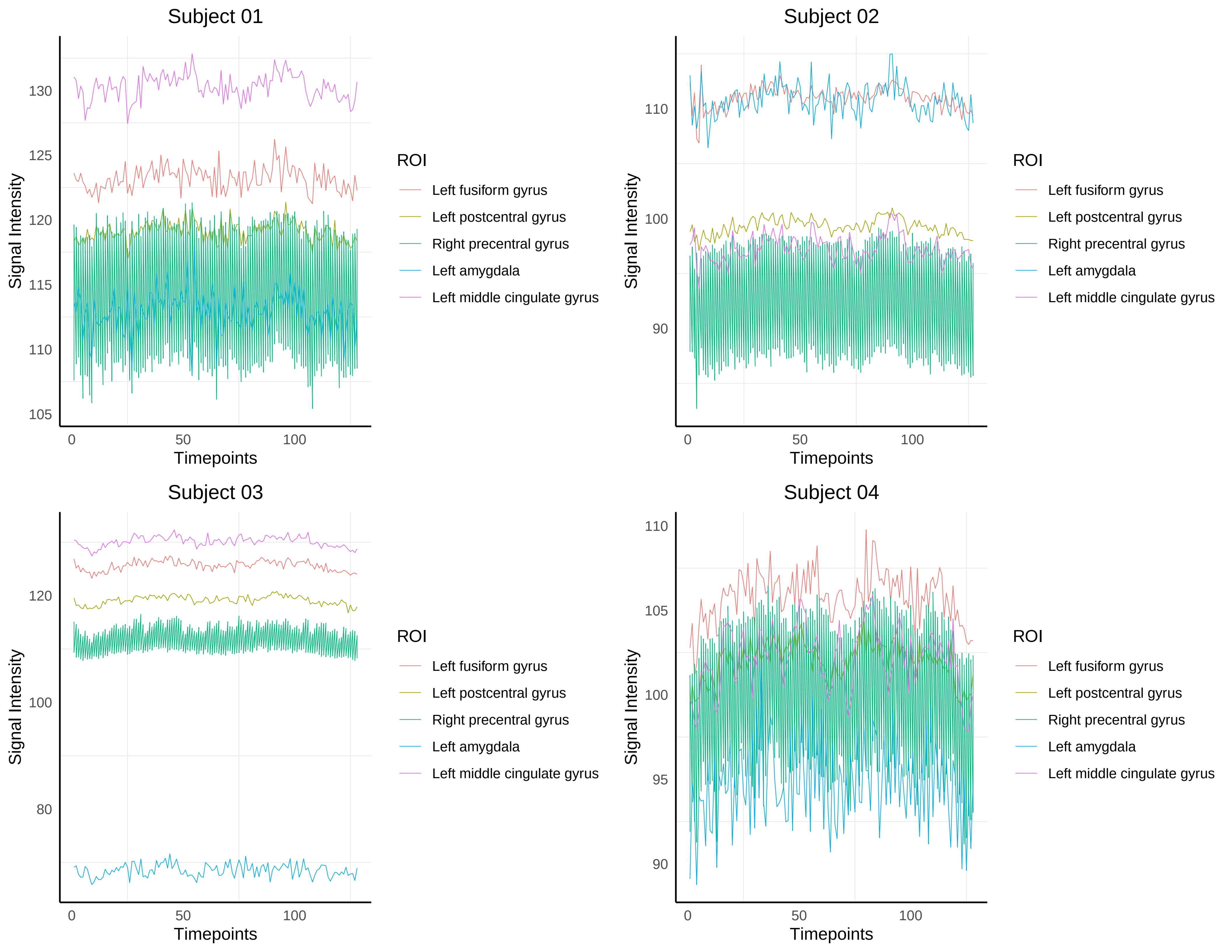

Subject 01
Subject 02
Subject 03
Subject 04
Signal Intensity
Timepoints
130
125
120
115
110
105
100
90
80
95
0
50
100
ROI
Left fusiform gyrus
Left postcentral gyrus
Right precentral gyrus
Left amygdala
Left middle cingulate gyrus

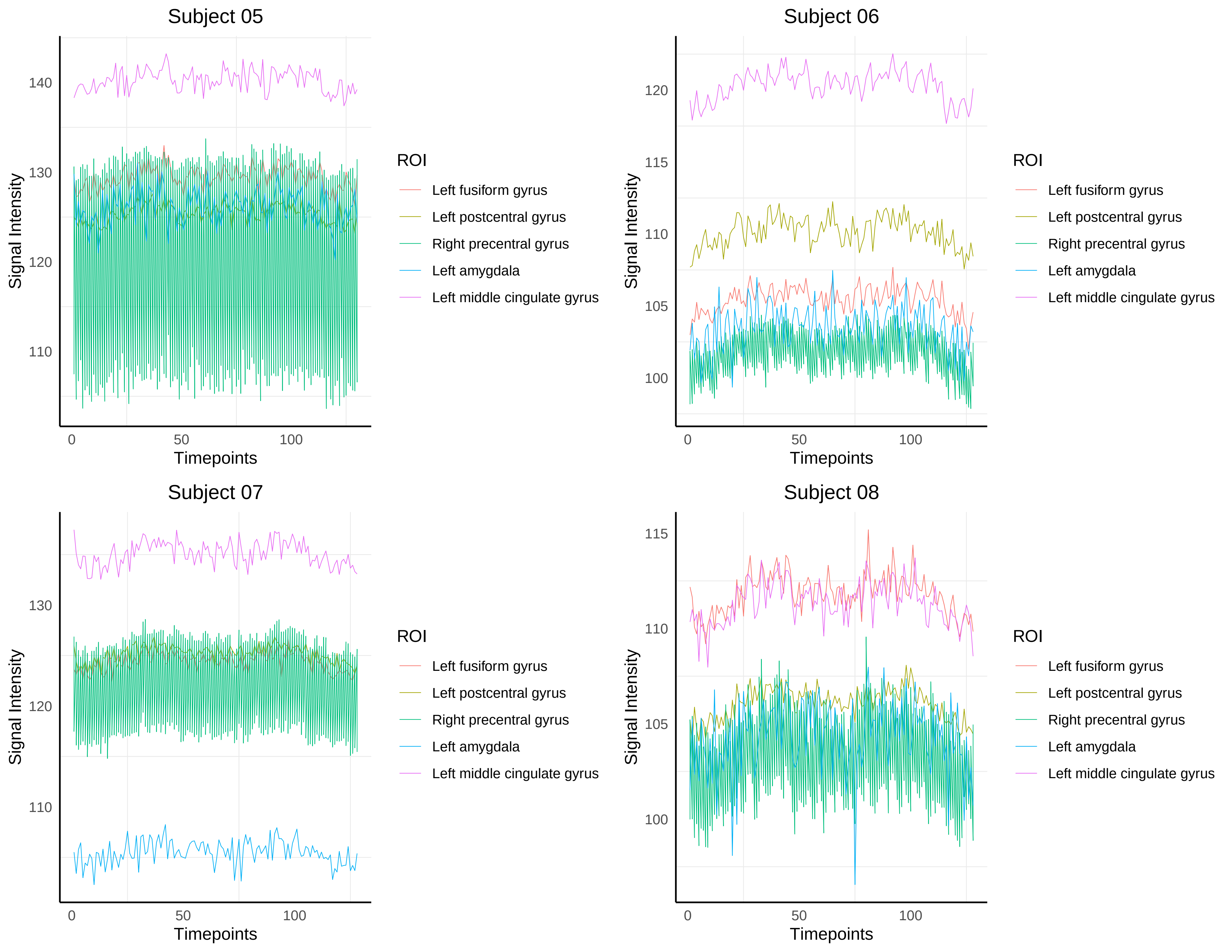

Subject 05
Subject 06
Subject 07
Subject 08
Signal Intensity
Timepoints
0
50
100
140
130
120
110
115
105
ROI
Left fusiform gyrus
Left postcentral gyrus
Right precentral gyrus
Left amygdala
Left middle cingulate gyrus

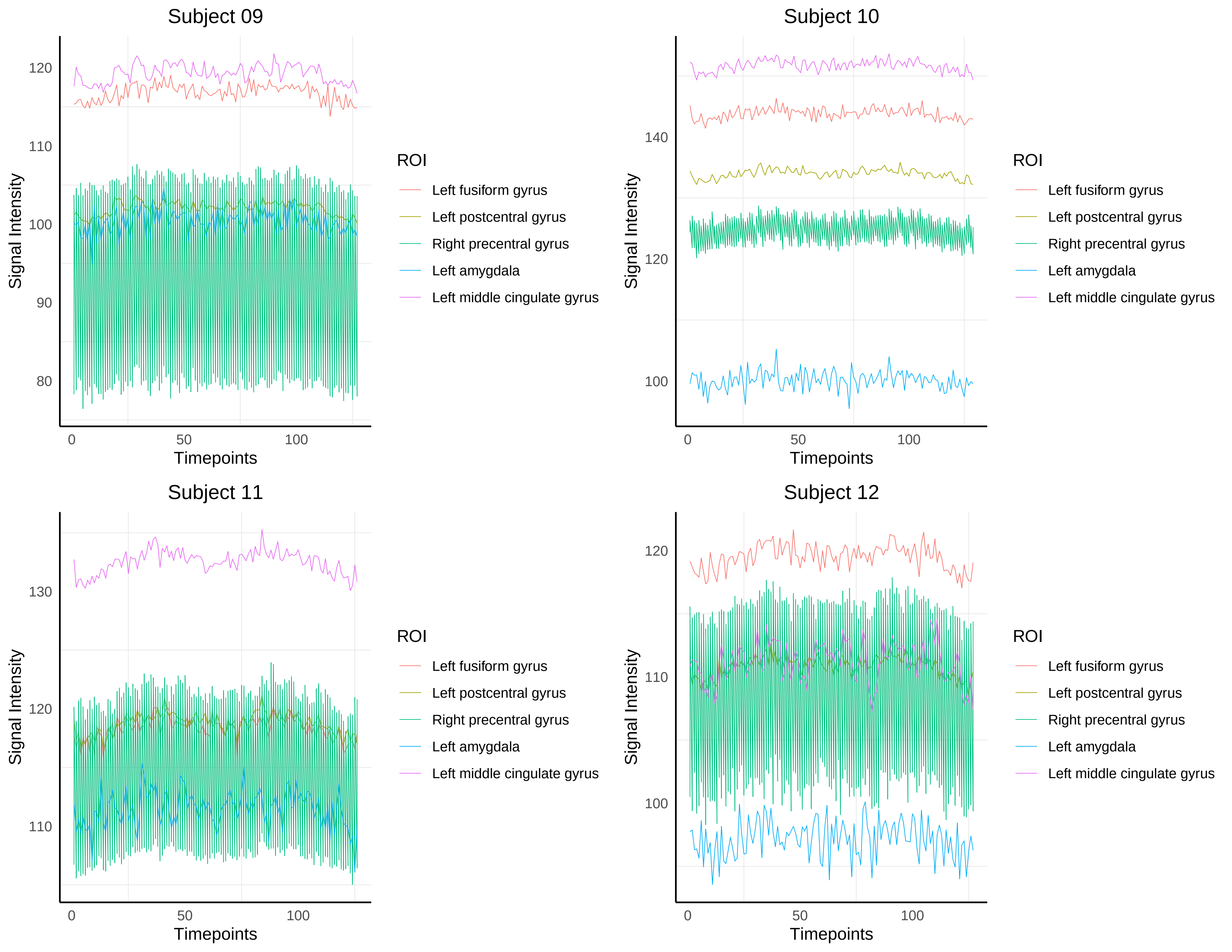
Subject 09
Subject 10
Subject 11
Subject 12
Signal Intensity
Timepoints
ROI
Left fusiform gyrus
Left postcentral gyrus
Right precentral gyrus
Left amygdala
Left middle cingulate gyrus

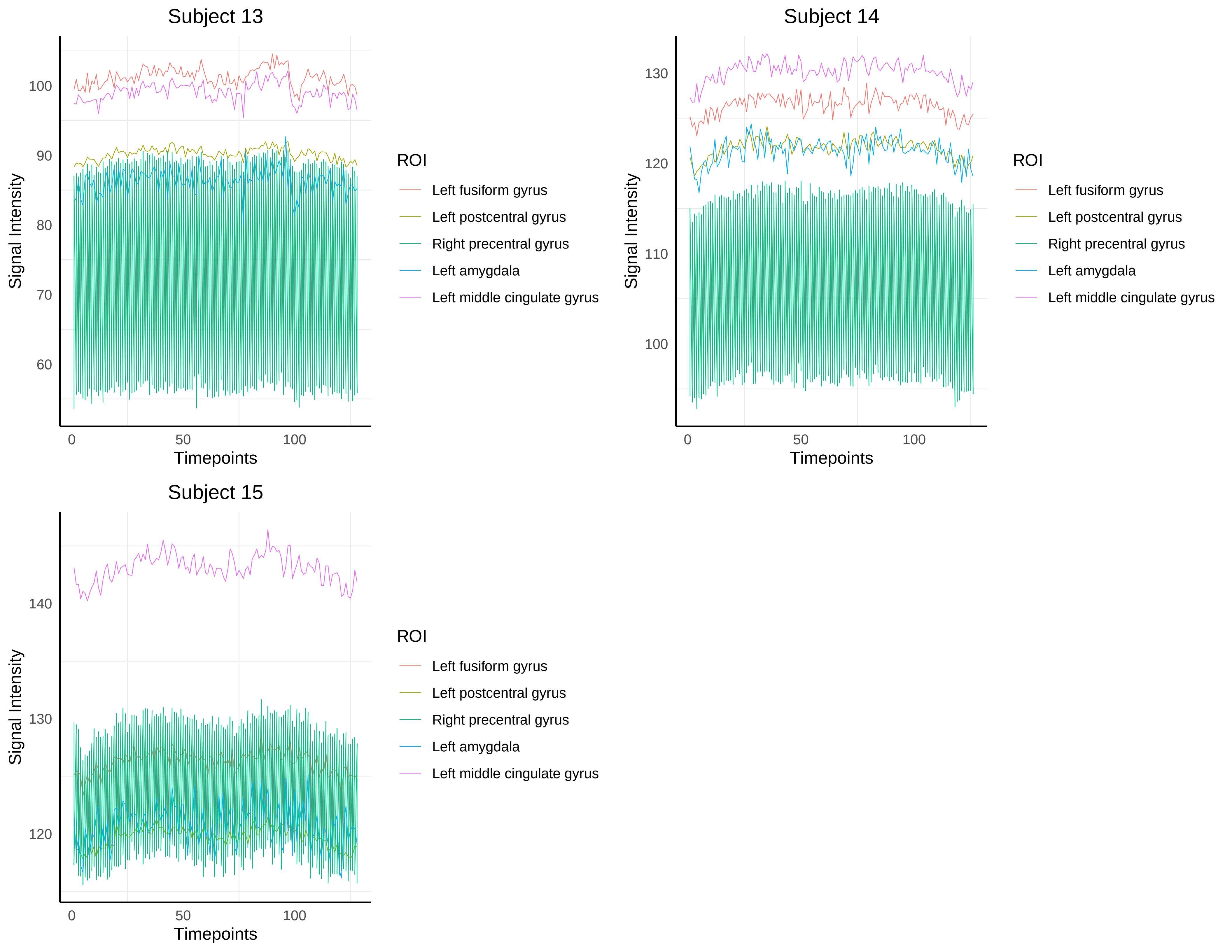

Subject 13
Subject 14
Subject 15
Signal Intensity
Timepoints
ROI
Left fusiform gyrus
Left postcentral gyrus
Right precentral gyrus
Left amygdala
Left middle cingulate gyrus

## Temporal Signal to Noise Ratio (tSNR)

| Subject | Left fusiform gyrus | Left postcentral gyrus | Right precentral gyrus | Left amygdala | Left middle cingulate gyrus | Right precentral gyrus |
|---|---|---|---|---|---|---|
| sub_01 | 129.61669 | 150.51446 | 106.70228 | 69.60798 | 131.12800 | 155.36009 |
| sub_02 | 108.57721 | 132.06413 | 95.09691 | 73.91130 | 79.12268 | 127.66027 |
| sub_03 | 134.59358 | 143.84779 | 109.42766 | 56.42019 | 136.62069 | 168.98035 |
| sub_04 | 71.13906 | 86.10867 | 68.92697 | 36.58643 | 55.82869 | 92.48318 |
| sub_05 | 102.35670 | 120.57214 | 72.41405 | 61.96588 | 114.64619 | 129.30545 |
| sub_06 | 111.93777 | 105.10820 | 108.91632 | 65.36013 | 116.64283 | 126.25410 |
| sub_07 | 134.17140 | 145.65333 | 140.63781 | 87.51876 | 111.13974 | 137.86724 |
| sub_08 | 103.55691 | 123.22312 | 104.55182 | 52.83623 | 101.03106 | 96.12700 |
| sub_09 | 108.51680 | 115.93430 | 79.96456 | 64.95341 | 104.36314 | 107.49054 |
| sub_10 | 155.68765 | 174.31565 | 144.20778 | 65.52847 | 161.84880 | 156.48864 |
| sub_11 | 123.45236 | 134.38937 | 141.17291 | 71.86946 | 134.54866 | 118.29762 |
| sub_12 | 120.59143 | 137.73811 | 81.07124 | 64.67577 | 72.01021 | 126.96071 |
| sub_13 | 77.07198 | 103.25629 | 59.69471 | 45.15298 | 74.71202 | 94.30454 |
| sub_14 | 111.11557 | 117.90783 | 100.15620 | 85.40867 | 110.22575 | 124.24041 |
| sub_15 | 129.23268 | 139.65583 | 125.98949 | 66.16986 | 118.06730 | 119.39011 |

# Supplementary Material 3
# Signal Stability

## Task 3 - Sentence Completion

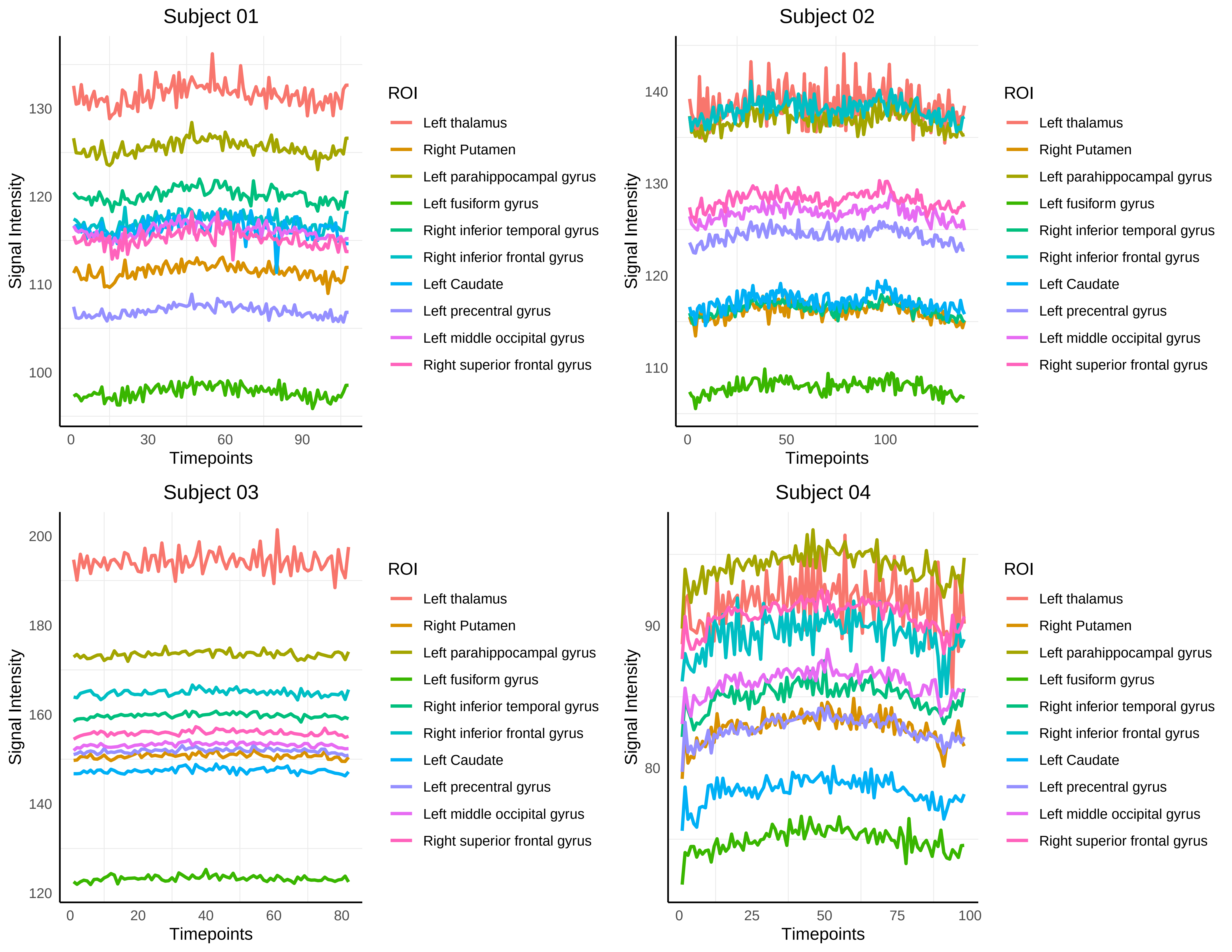

Subject 01
Subject 02
Subject 03
Subject 04
Signal Intensity
Timepoints
ROI
Left thalamus
Right Putamen
Left parahippocampal gyrus
Left fusiform gyrus
Right inferior temporal gyrus
Right inferior frontal gyrus
Left Caudate
Left precentral gyrus
Left middle occipital gyrus
Right superior frontal gyrus

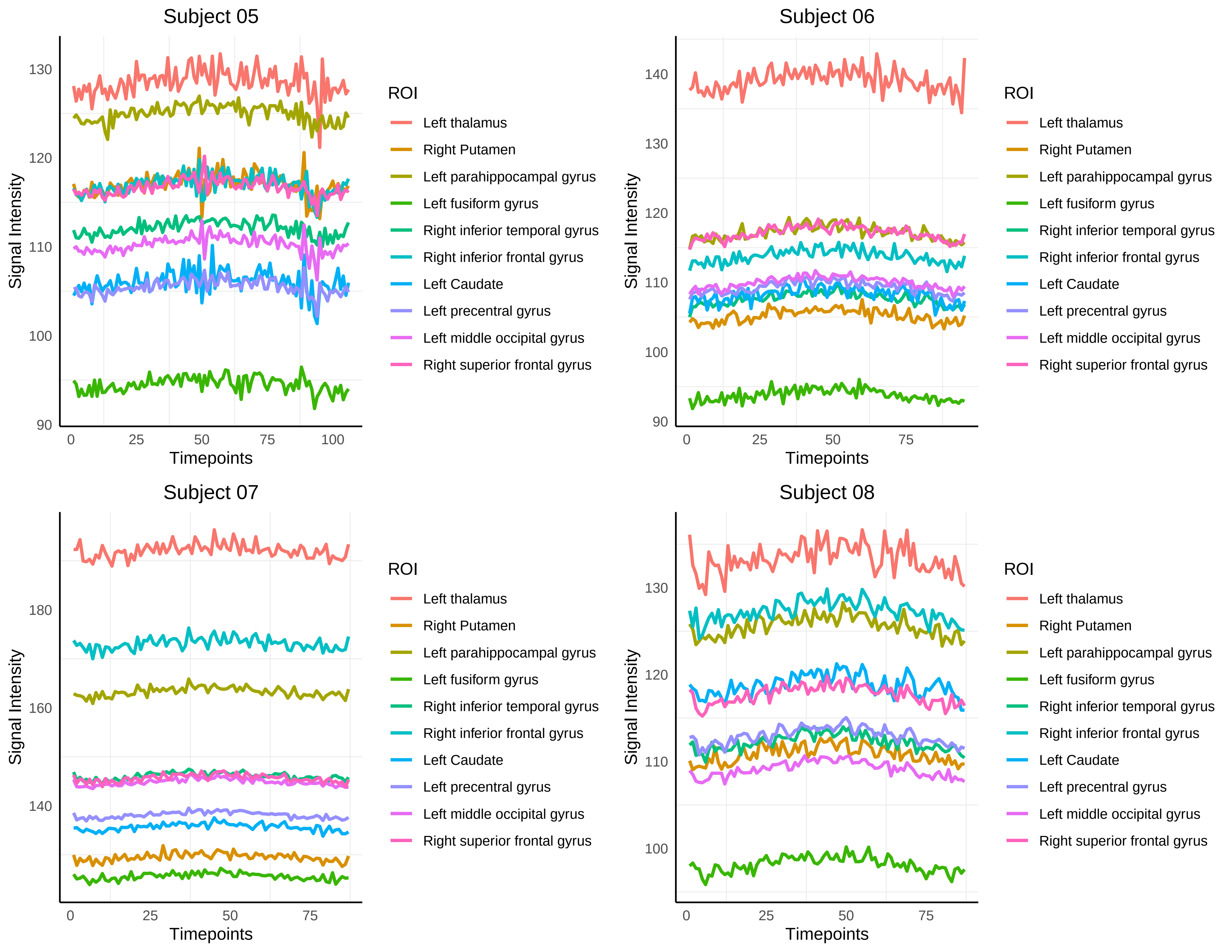

Subject 05
Subject 06
Subject 07
Subject 08
Signal Intensity
Timepoints
ROI
Left thalamus
Right Putamen
Left parahippocampal gyrus
Left fusiform gyrus
Right inferior temporal gyrus
Right inferior frontal gyrus
Left Caudate
Left precentral gyrus
Left middle occipital gyrus
Right superior frontal gyrus

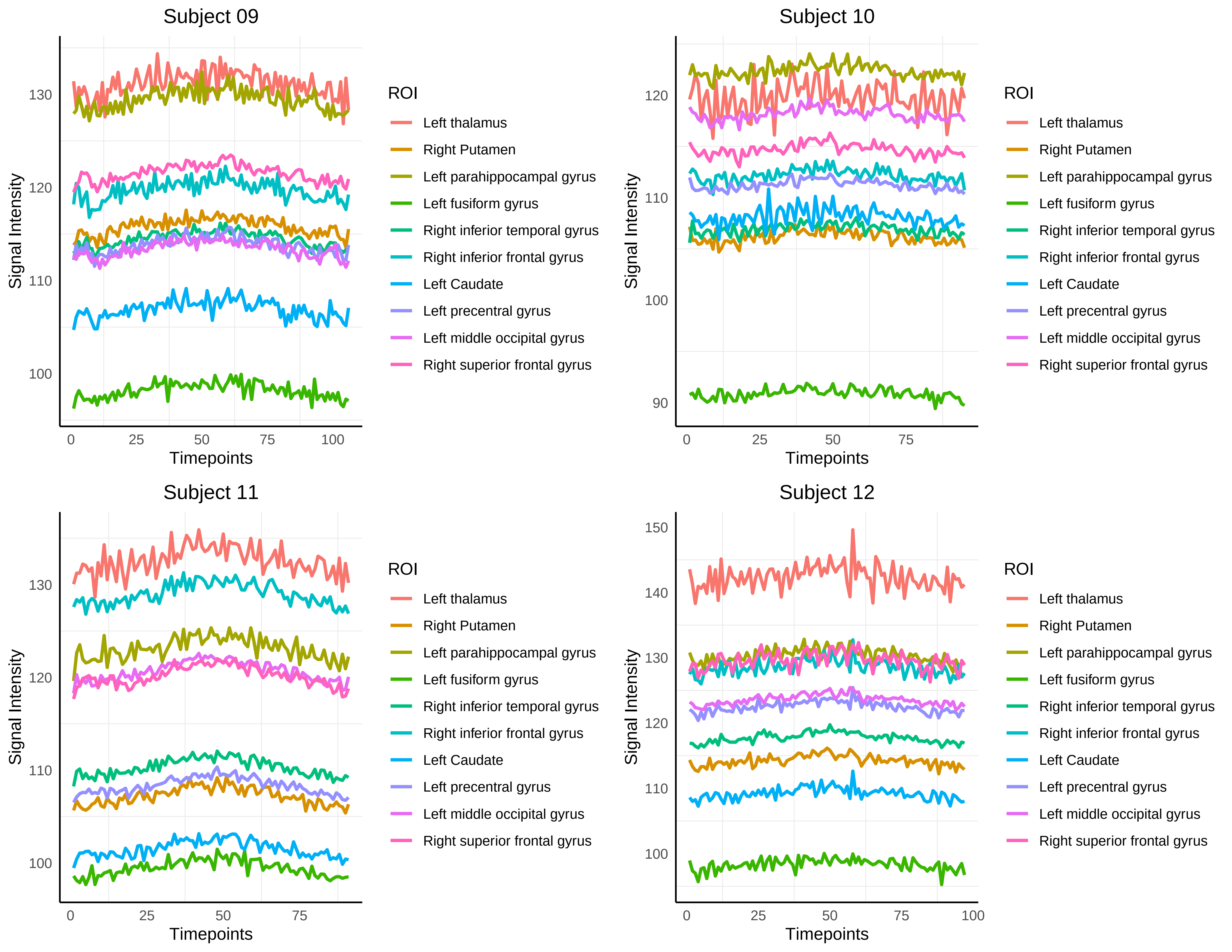
Subject 09
Signal Intensity
130
120
110
100
0
25
50
75
100
Timepoints
ROI
Left thalamus
Right Putamen
Left parahippocampal gyrus
Left fusiform gyrus
Right inferior temporal gyrus
Right inferior frontal gyrus
Left Caudate
Left precentral gyrus
Left middle occipital gyrus
Right superior frontal gyrus
Subject 10
Signal Intensity
120
110
100
90
0
25
50
75
Timepoints
ROI
Left thalamus
Right Putamen
Left parahippocampal gyrus
Left fusiform gyrus
Right inferior temporal gyrus
Right inferior frontal gyrus
Left Caudate
Left precentral gyrus
Left middle occipital gyrus
Right superior frontal gyrus
Subject 11
Signal Intensity
130
120
110
100
0
25
50
75
Timepoints
ROI
Left thalamus
Right Putamen
Left parahippocampal gyrus
Left fusiform gyrus
Right inferior temporal gyrus
Right inferior frontal gyrus
Left Caudate
Left precentral gyrus
Left middle occipital gyrus
Right superior frontal gyrus
Subject 12
Signal Intensity
150
140
130
120
110
100
0
25
50
75
100
Timepoints
ROI
Left thalamus
Right Putamen
Left parahippocampal gyrus
Left fusiform gyrus
Right inferior temporal gyrus
Right inferior frontal gyrus
Left Caudate
Left precentral gyrus
Left middle occipital gyrus
Right superior frontal gyrus

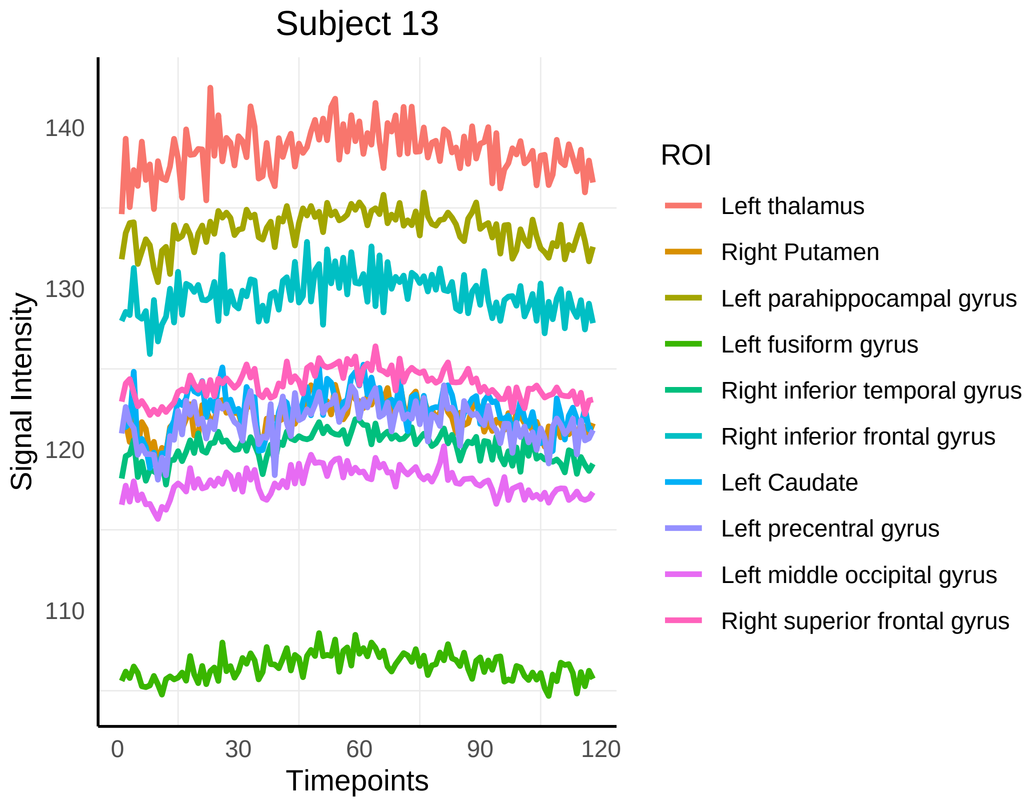
Subject 13
Signal Intensity
140
130
120
110
0
30
60
90
120
Timepoints
ROI
Left thalamus
Right Putamen
Left parahippocampal gyrus
Left fusiform gyrus
Right inferior temporal gyrus
Right inferior frontal gyrus
Left Caudate
Left precentral gyrus
Left middle occipital gyrus
Right superior frontal gyrus

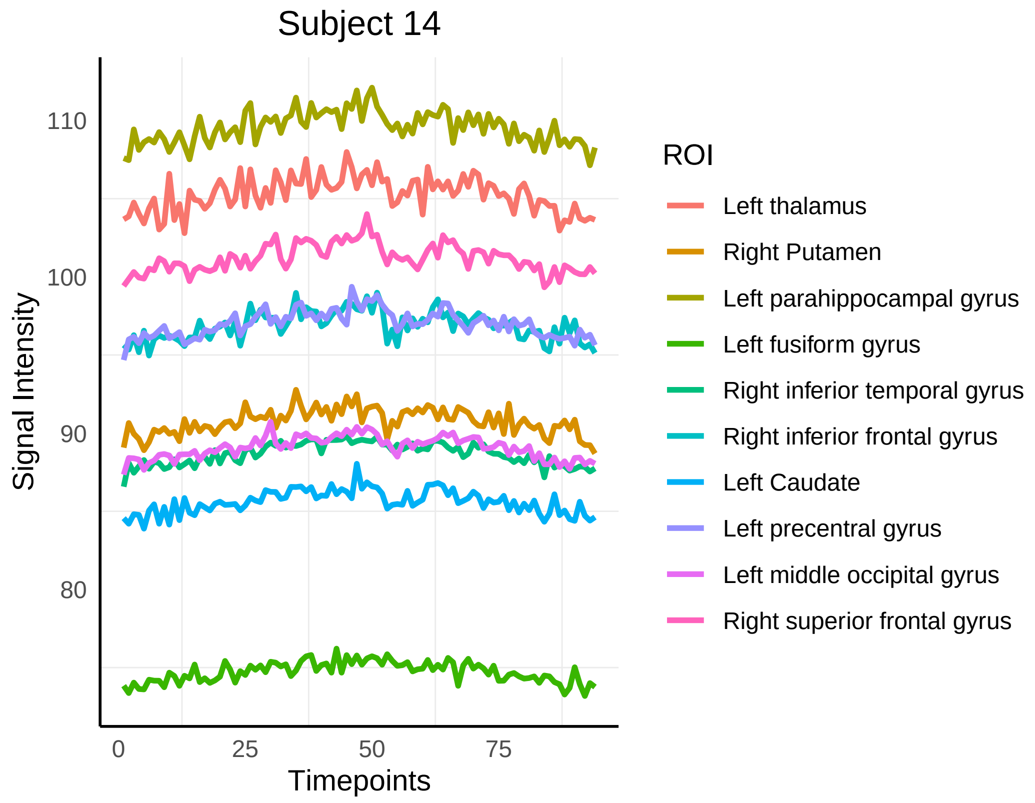
Subject 14
Signal Intensity
110
100
90
80
0
25
50
75
Timepoints
ROI
Left thalamus
Right Putamen
Left parahippocampal gyrus
Left fusiform gyrus
Right inferior temporal gyrus
Right inferior frontal gyrus
Left Caudate
Left precentral gyrus
Left middle occipital gyrus
Right superior frontal gyrus

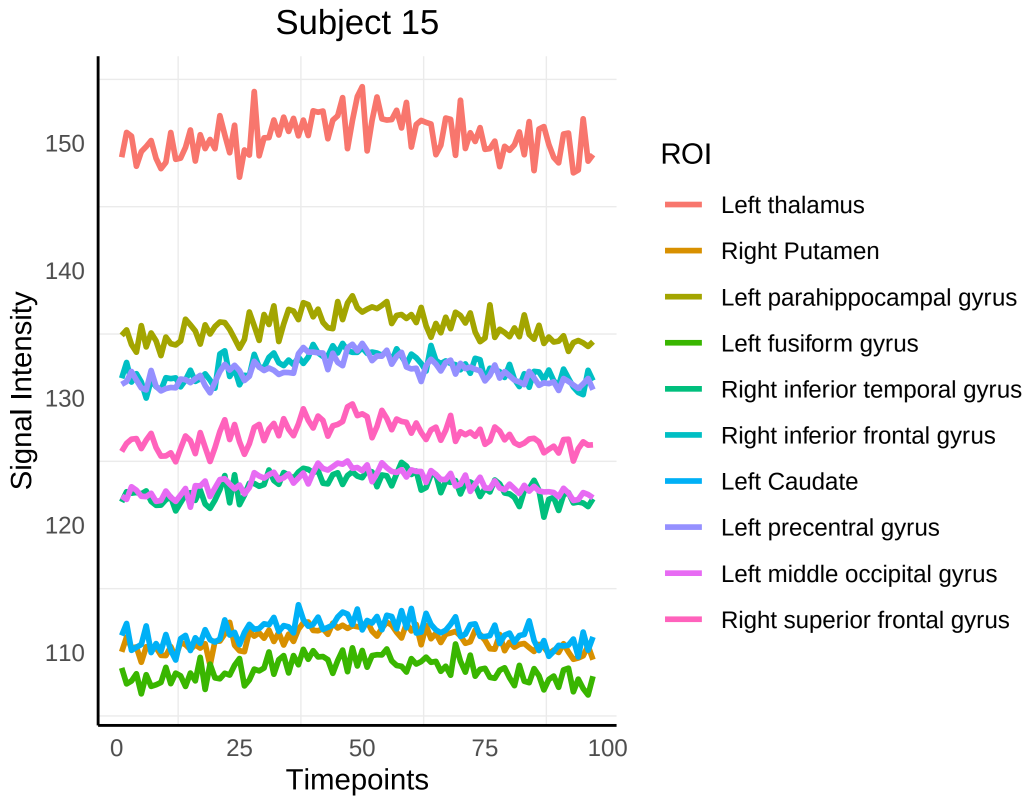
Subject 15
Signal Intensity
150
140
130
120
110
0
25
50
75
100
Timepoints
ROI
Left thalamus
Right Putamen
Left parahippocampal gyrus
Left fusiform gyrus
Right inferior temporal gyrus
Right inferior frontal gyrus
Left Caudate
Left precentral gyrus
Left middle occipital gyrus
Right superior frontal gyrus

# Temporal Signal to Noise Ratio (tSNR)

| Subject | Left thalamus | Right Putamen | Left parahippocampal gyrus | Left fusiform gyrus | Right inferior temporal gyrus | Right inferior frontal gyrus | Left Caudate | Left precentral gyrus | Left middle occipital gyrus | Right superior frontal gyrus |
|---|---|---|---|---|---|---|---|---|---|---|
| sub_01 | 102.59623 | 138.53905 | 138.05688 | 127.31474 | 147.72163 | 138.47064 | 103.18900 | 167.92316 | 155.12302 | 121.46961 |
| sub_02 | 72.73519 | 137.13750 | 131.53827 | 138.13245 | 160.45180 | 127.19595 | 124.84345 | 156.66109 | 153.10100 | 150.21353 |
| sub_03 | 83.31463 | 263.97286 | 250.10201 | 201.35966 | 318.38901 | 241.67590 | 253.16168 | 312.69877 | 342.25791 | 299.90347 |
| sub_04 | 47.85195 | 83.08846 | 87.63013 | 92.35985 | 88.30792 | 63.85542 | 87.13978 | 98.60295 | 92.23485 | 91.51664 |
| sub_05 | 83.00861 | 90.44769 | 126.70465 | 108.66225 | 134.59434 | 101.16514 | 78.67241 | 124.31670 | 115.20588 | 128.82302 |
| sub_06 | 84.92222 | 114.26884 | 115.12259 | 105.67274 | 124.54903 | 115.85513 | 97.55448 | 124.06362 | 134.73740 | 125.16694 |
| sub_07 | 131.76131 | 156.51374 | 175.01865 | 178.00331 | 200.69613 | 142.84393 | 187.12196 | 218.30336 | 216.61929 | 193.24605 |
| sub_08 | 78.29819 | 109.26972 | 109.86446 | 110.30226 | 120.81191 | 105.41732 | 96.43650 | 119.85806 | 125.57893 | 113.60227 |
| sub_09 | 92.62073 | 128.42710 | 113.94940 | 114.42909 | 137.92228 | 109.72774 | 108.06793 | 126.59829 | 133.86778 | 135.35305 |
| sub_10 | 78.73282 | 171.84163 | 181.26566 | 180.42336 | 203.08999 | 178.15565 | 128.49865 | 230.82702 | 198.87726 | 195.13534 |
| sub_11 | 88.01347 | 113.48794 | 100.58752 | 111.86087 | 119.89068 | 111.83667 | 116.58810 | 123.58886 | 117.13557 | 116.27187 |
| sub_12 | 75.63311 | 136.72268 | 121.82546 | 108.06916 | 155.48444 | 108.66976 | 119.82985 | 139.78861 | 158.17301 | 101.85181 |
| sub_13 | 92.82654 | 122.23455 | 124.30407 | 131.88760 | 134.04855 | 98.71259 | 88.26963 | 100.95160 | 138.18412 | 136.11684 |
| sub_14 | 92.45523 | 109.84934 | 105.78638 | 112.80750 | 127.71073 | 104.55332 | 113.18021 | 116.43173 | 125.91730 | 112.59844 |
| sub_15 | 95.91690 | 131.71218 | 122.58418 | 118.19747 | 127.91555 | 129.55588 | 118.95670 | 136.24081 | 143.30542 | 126.16647 |

# Supplementary Material 3
# Signal Stability

## Task 4 - Pyramids and Palm Trees Test

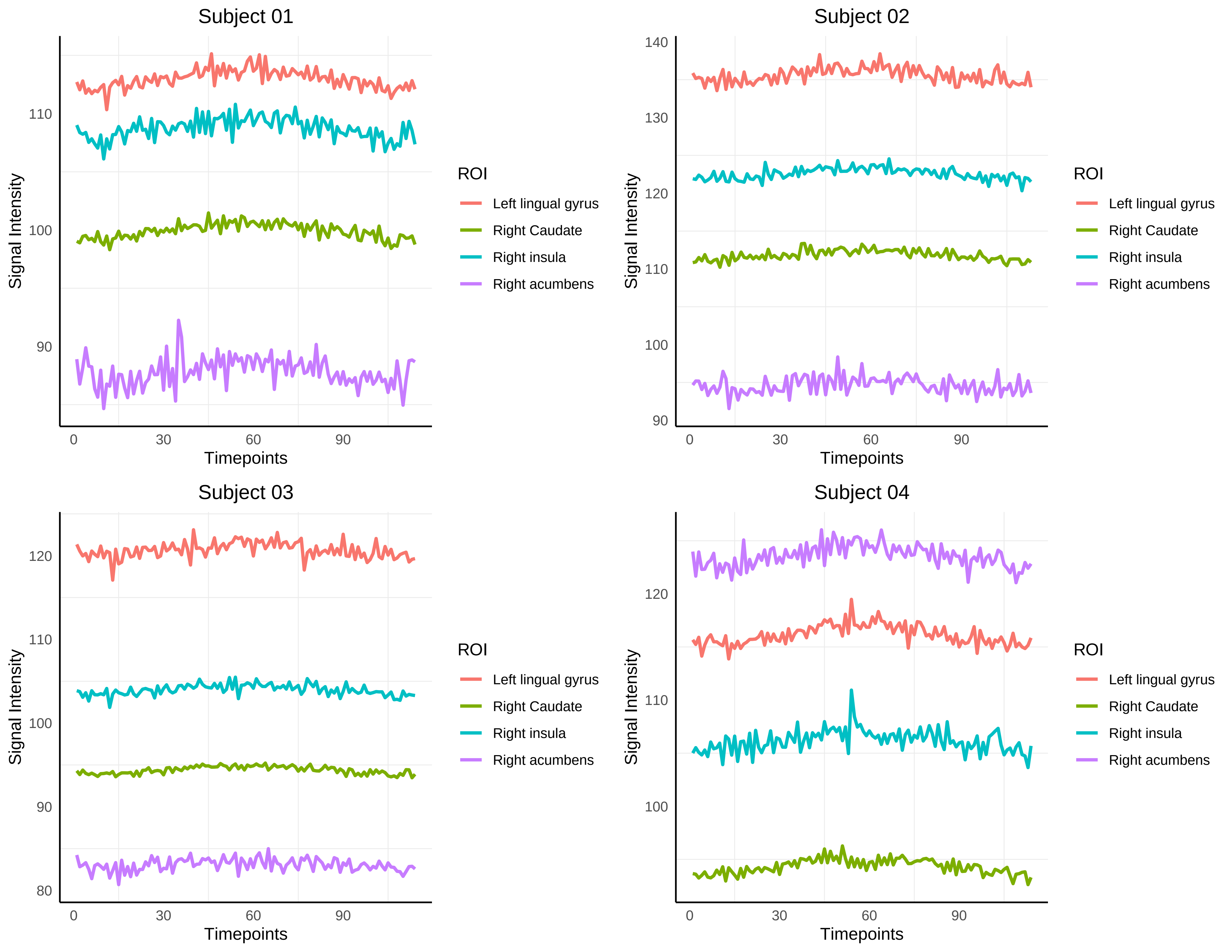

Subject 01
Subject 02
Subject 03
Subject 04
Signal Intensity
Timepoints
0
30
60
90
ROI
Left lingual gyrus
Right Caudate
Right insula
Right acumbens

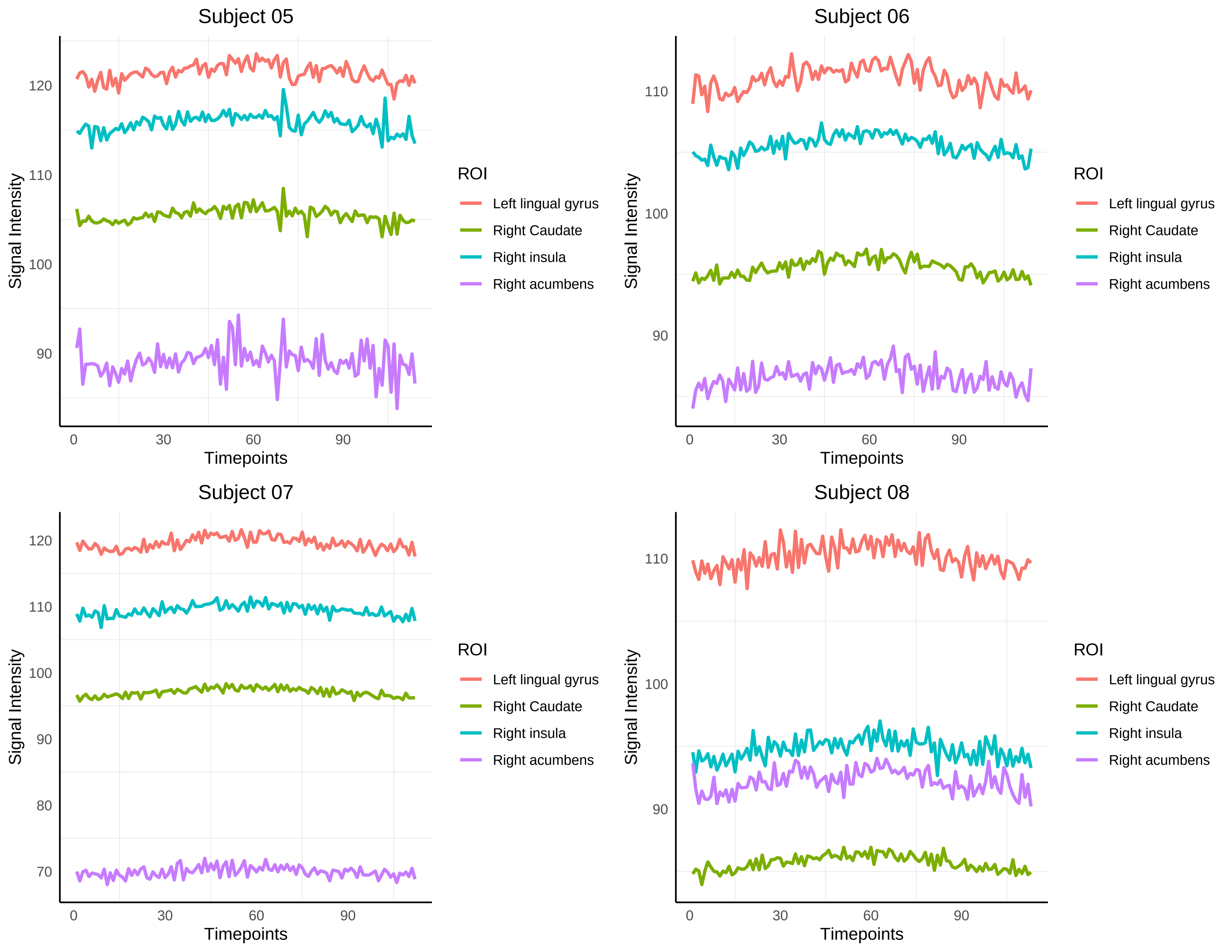
Subject 05
Subject 06
Subject 07
Subject 08
Signal Intensity
Timepoints
ROI
Left lingual gyrus
Right Caudate
Right insula
Right acumbens
0
30
60
90
70
80
90
100
110
120

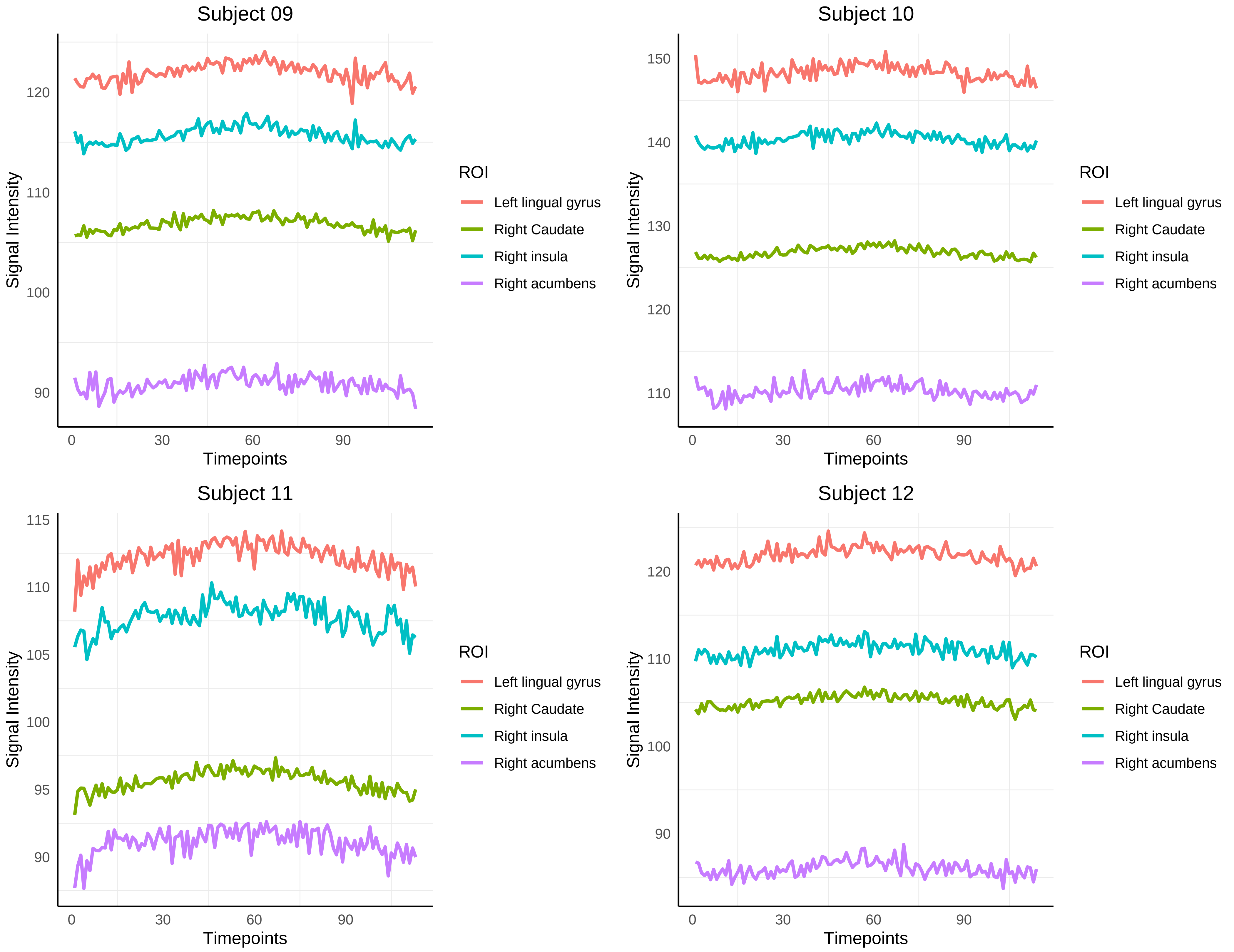
Subject 09
Signal Intensity
120
110
100
90
0
30
60
90
Timepoints
ROI
Left lingual gyrus
Right Caudate
Right insula
Right acumbens
Subject 10
Signal Intensity
150
140
130
120
110
0
30
60
90
Timepoints
ROI
Left lingual gyrus
Right Caudate
Right insula
Right acumbens
Subject 11
Signal Intensity
115
110
105
100
95
90
0
30
60
90
Timepoints
ROI
Left lingual gyrus
Right Caudate
Right insula
Right acumbens
Subject 12
Signal Intensity
120
110
100
90
0
30
60
90
Timepoints
ROI
Left lingual gyrus
Right Caudate
Right insula
Right acumbens

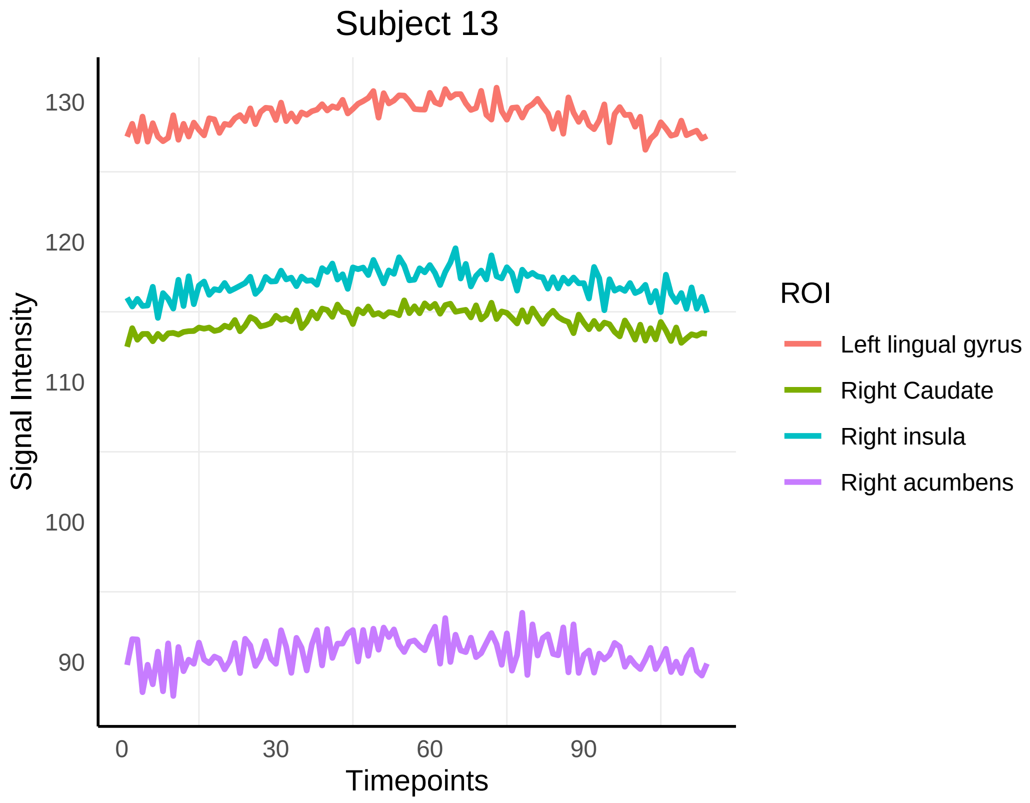

Subject 13
Signal Intensity
130
120
110
100
90
0
30
60
90
Timepoints
ROI
Left lingual gyrus
Right Caudate
Right insula
Right acumbens


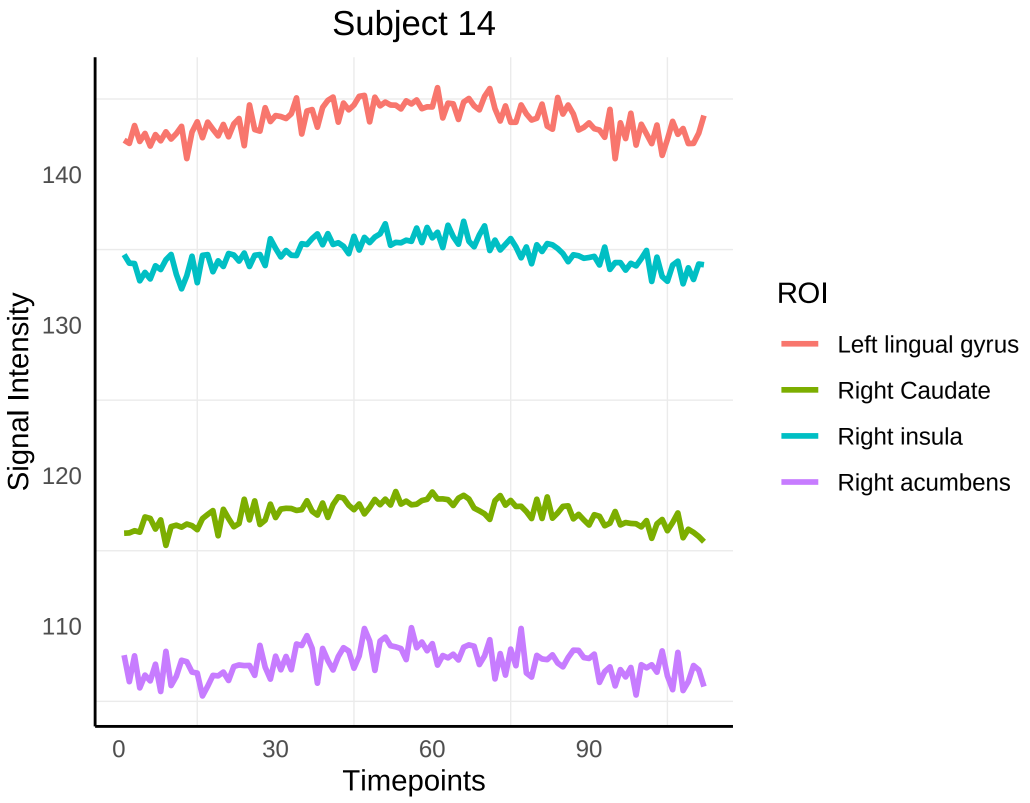

Subject 14
Signal Intensity
140
130
120
110
0
30
60
90
Timepoints
ROI
Left lingual gyrus
Right Caudate
Right insula
Right acumbens


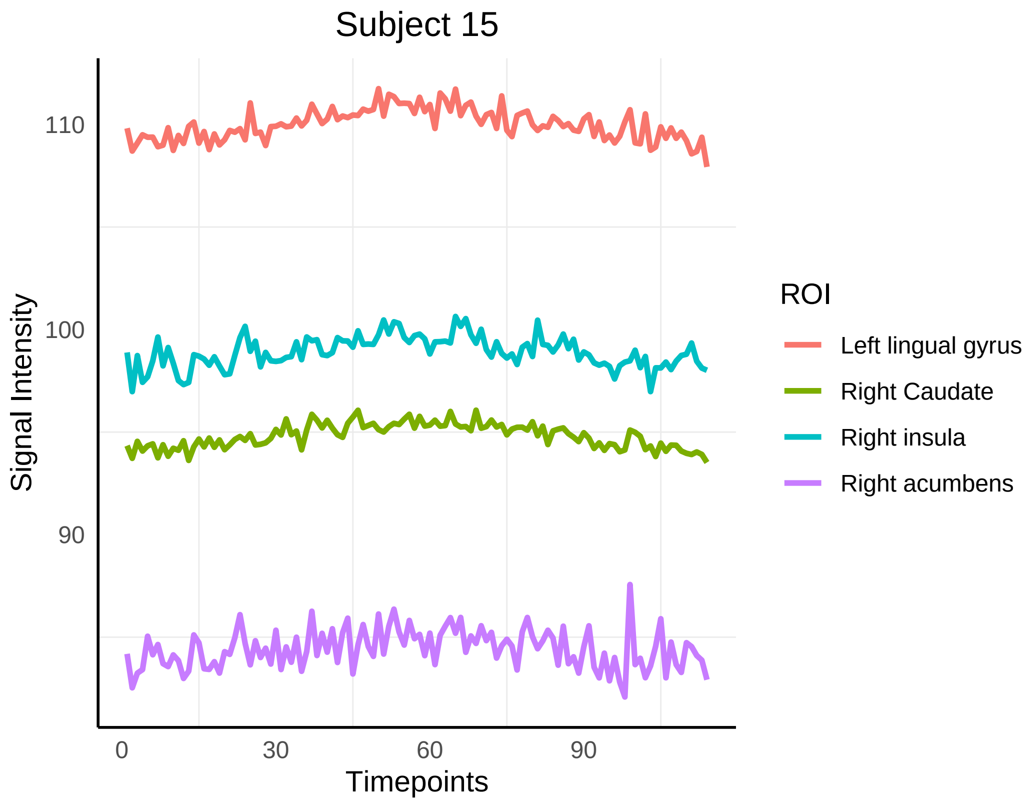

Subject 15
Signal Intensity
110
100
90
0
30
60
90
Timepoints
ROI
Left lingual gyrus
Right Caudate
Right insula
Right acumbens

# Temporal Signal to Noise Ratio (tSNR)

| Subject | Left lingual gyrus | Right Caudate | Right insula | Right acumbens |
|---|---|---|---|---|
| sub_01 | 138.4473 | 148.8916 | 116.0924 | 69.67941 |
| sub_02 | 132.4987 | 165.2276 | 161.1453 | 83.39467 |
| sub_03 | 126.0215 | 208.8818 | 161.6932 | 111.04631 |
| sub_04 | 124.1652 | 134.8735 | 99.3305 | 115.88901 |
| sub_05 | 123.3892 | 125.8717 | 109.5025 | 51.45133 |
| sub_06 | 109.0990 | 131.2537 | 124.2919 | 90.34738 |
| sub_07 | 122.5826 | 149.6442 | 124.4318 | 86.15489 |
| sub_08 | 104.7458 | 139.5141 | 104.3010 | 98.94625 |
| sub_09 | 127.2676 | 151.5589 | 137.2413 | 102.63498 |
| sub_10 | 149.3122 | 204.2282 | 174.6899 | 120.89418 |
| sub_11 | 105.7206 | 124.9234 | 101.1386 | 91.68095 |
| sub_12 | 127.6426 | 147.1444 | 121.0252 | 95.93246 |
| sub_13 | 128.1135 | 149.3856 | 121.6726 | 78.52470 |
| sub_14 | 136.5677 | 144.9962 | 140.0053 | 107.36059 |
| sub_15 | 143.1676 | 159.6409 | 129.7677 | 87.54868 |
