# Supplementary material for: A Rapid fMRI Paradigm for Localisation of the Language Network
Source: Eur J Neurosci. 2026 Mar 6;63(5):e70448. doi: 10.1111/ejn.70448 (PMC12964186; doi:10.1111/ejn.70448)

Combined Language Network for Subject 1

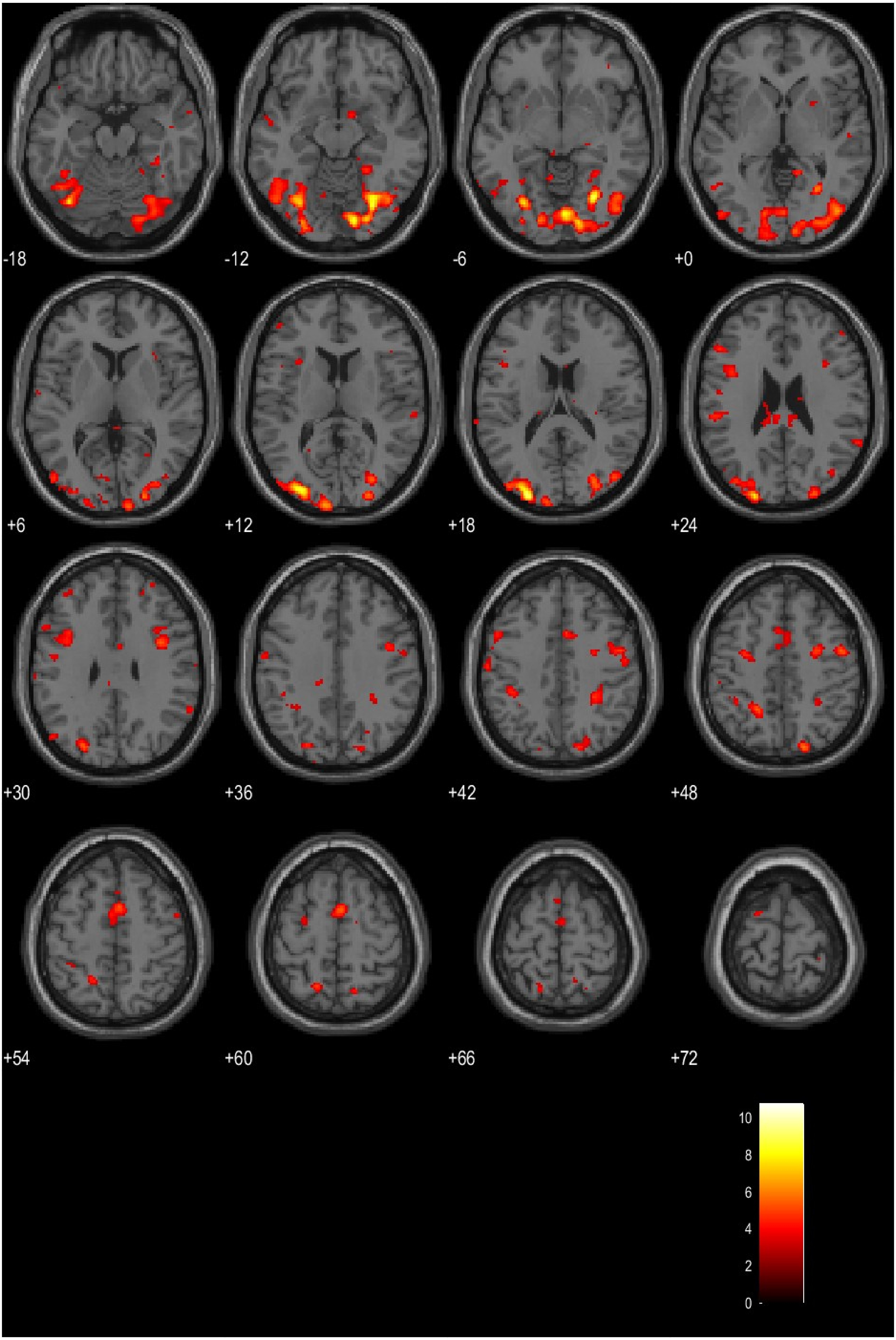

Combined Language Network for Subject 2

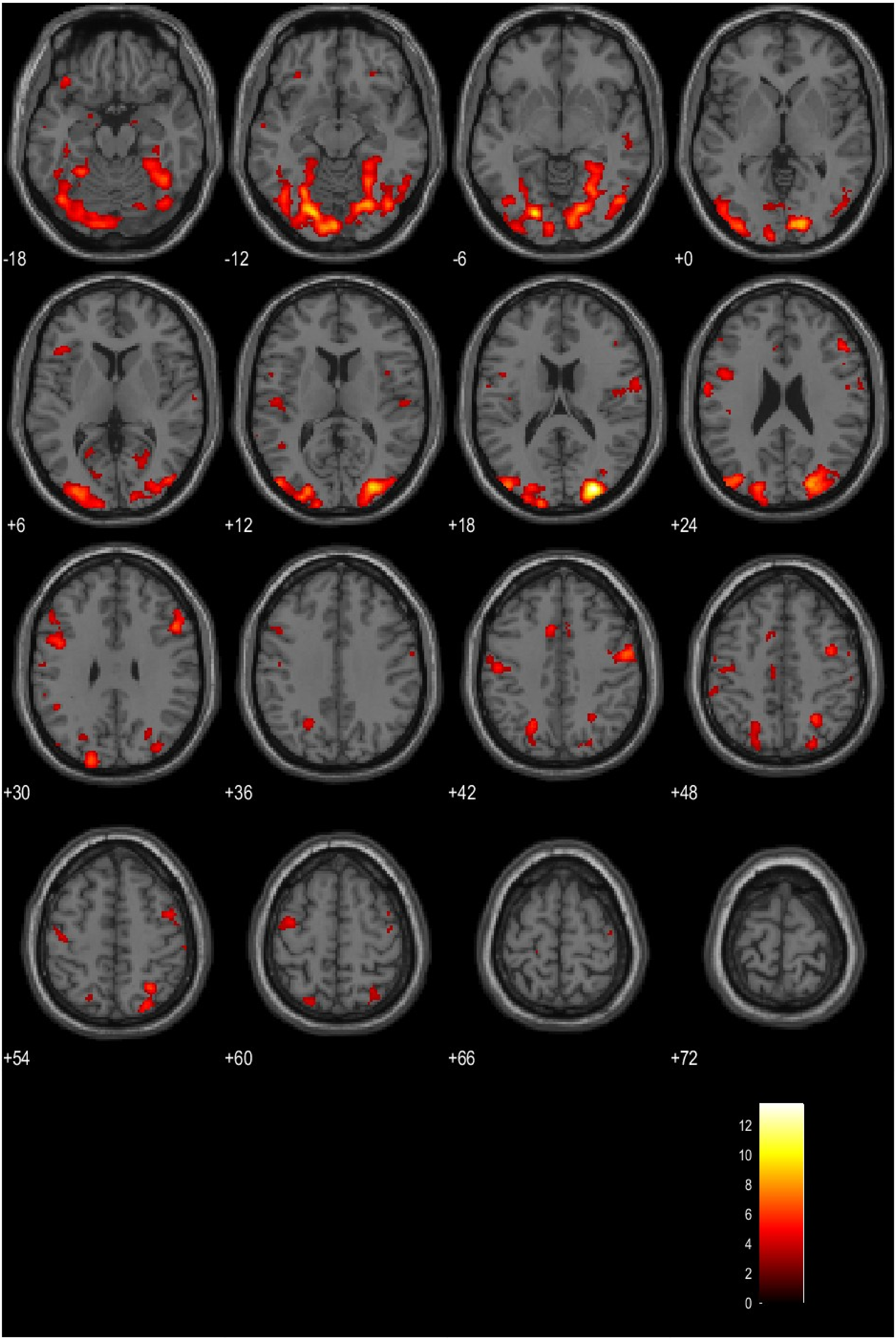

Combined Language Network for Subject 3

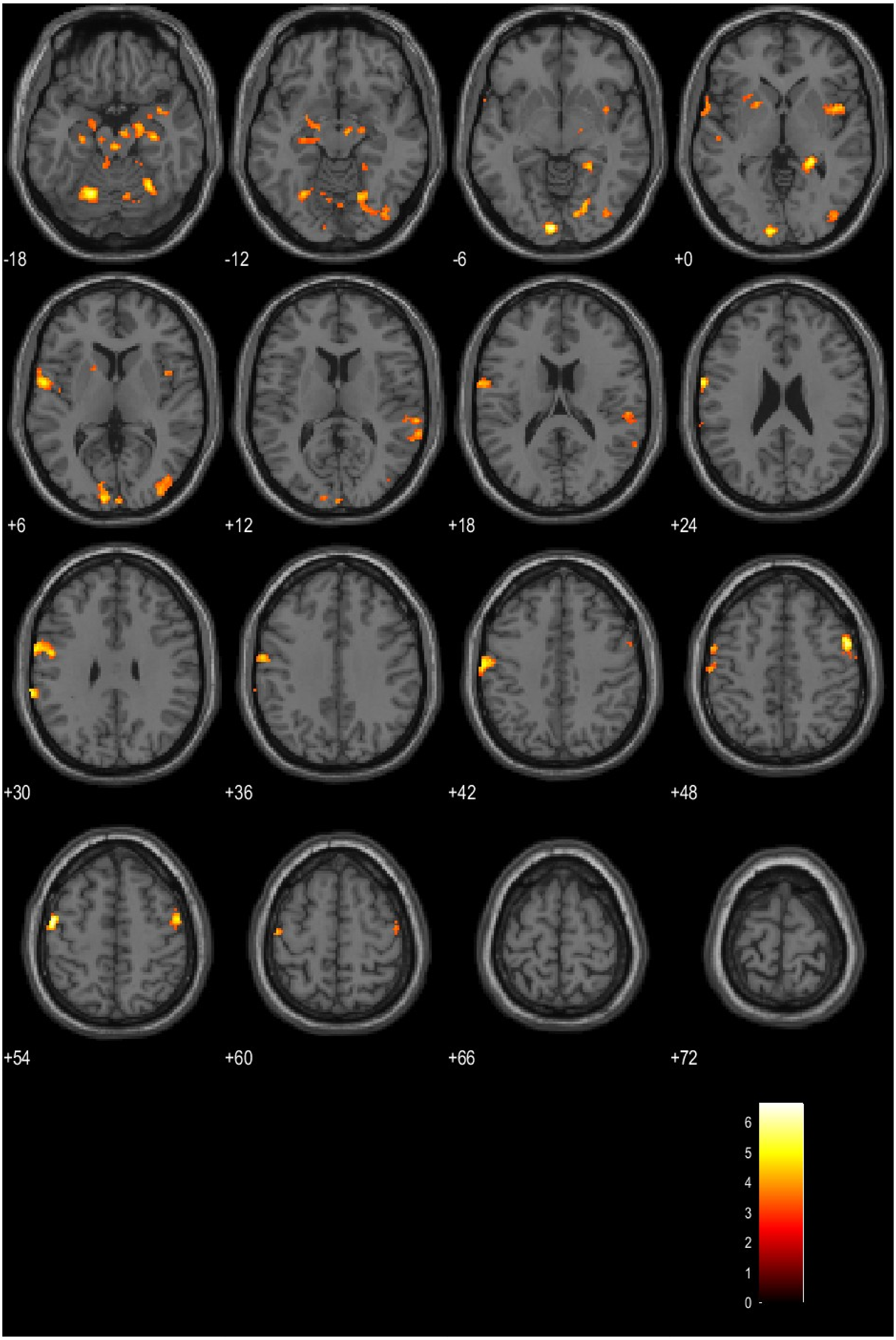

Combined Language Network for Subject 4

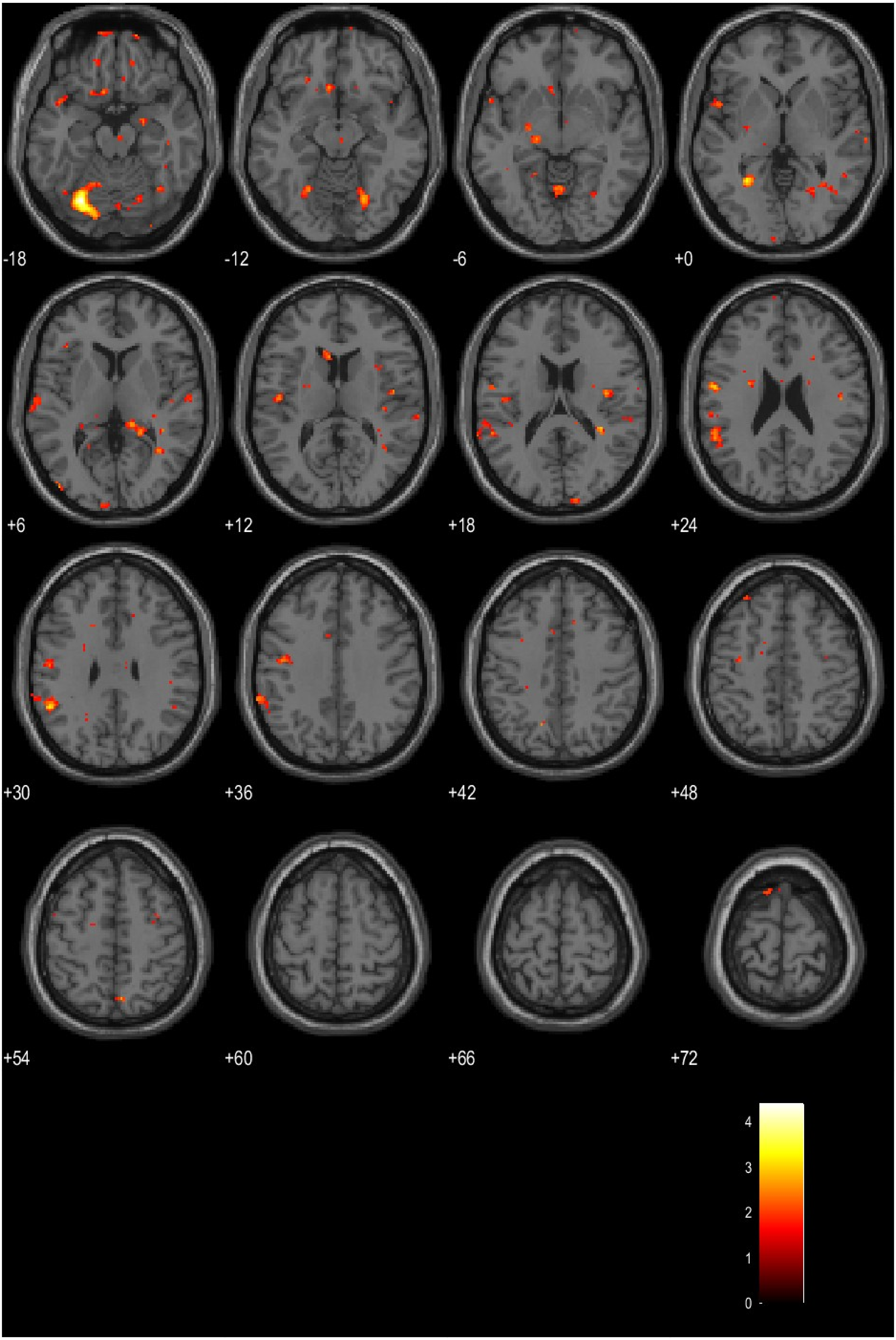

Combined Language Network for Subject 5

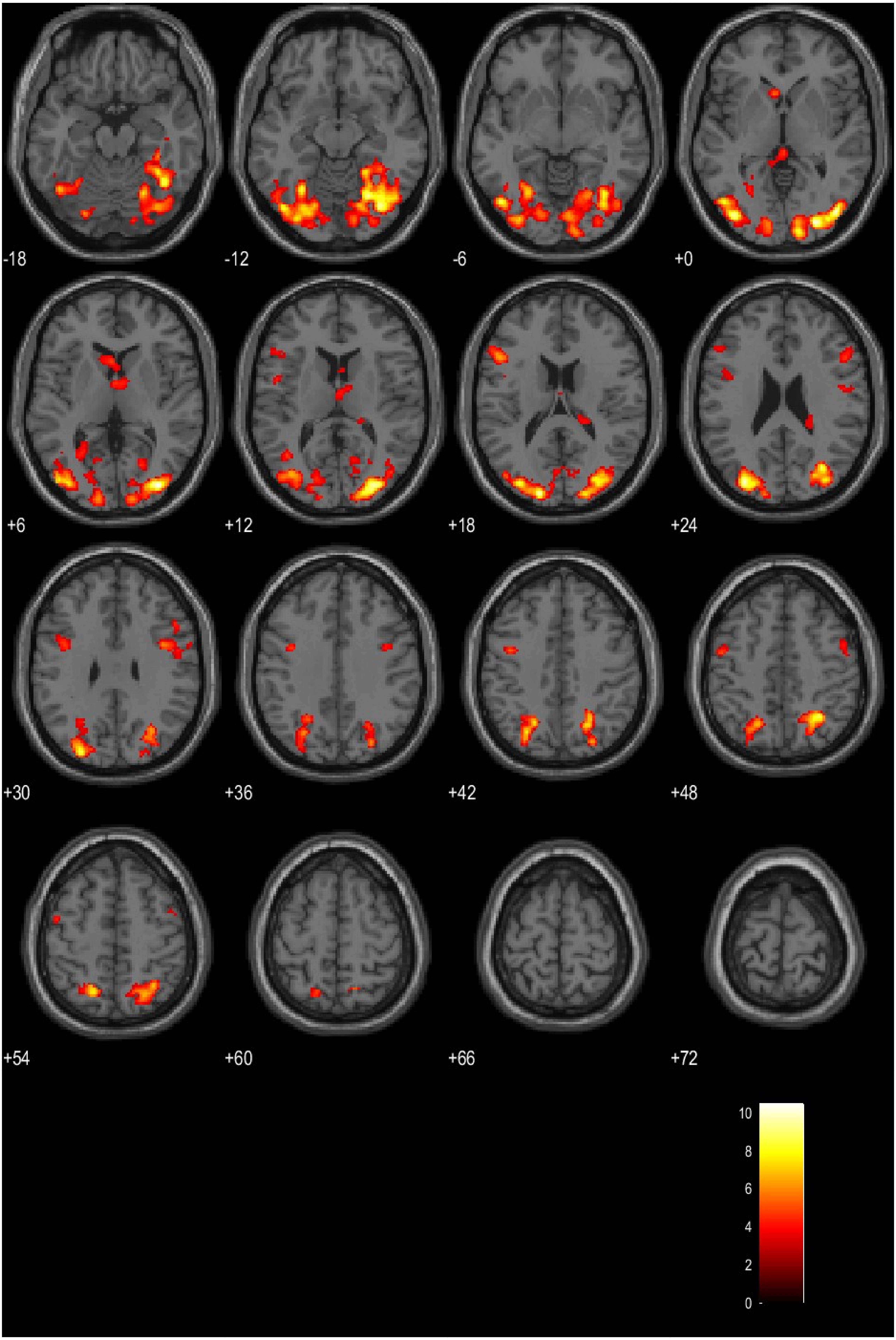

Combined Language Network for Subject 6

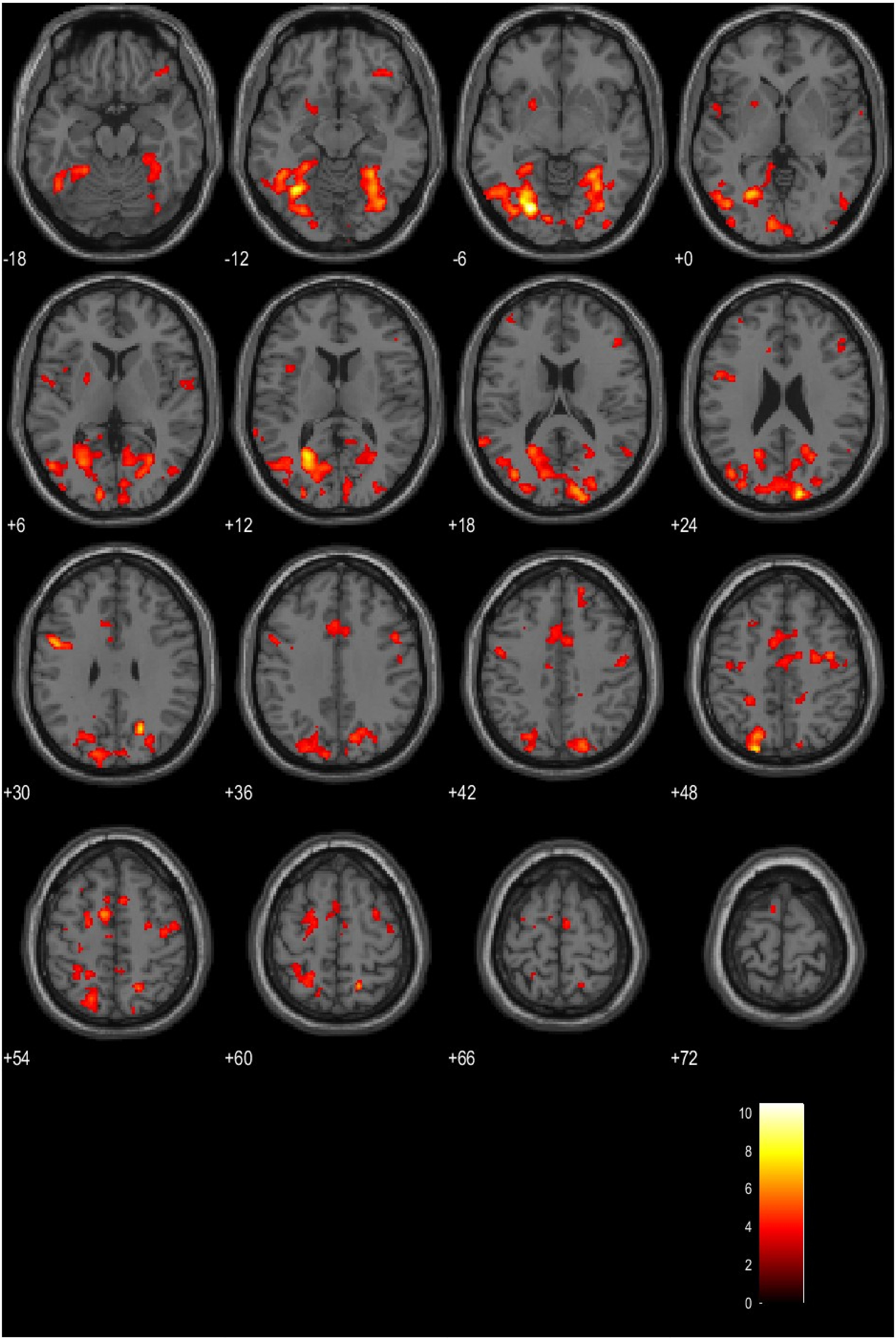

Combined Language Network for Subject 7

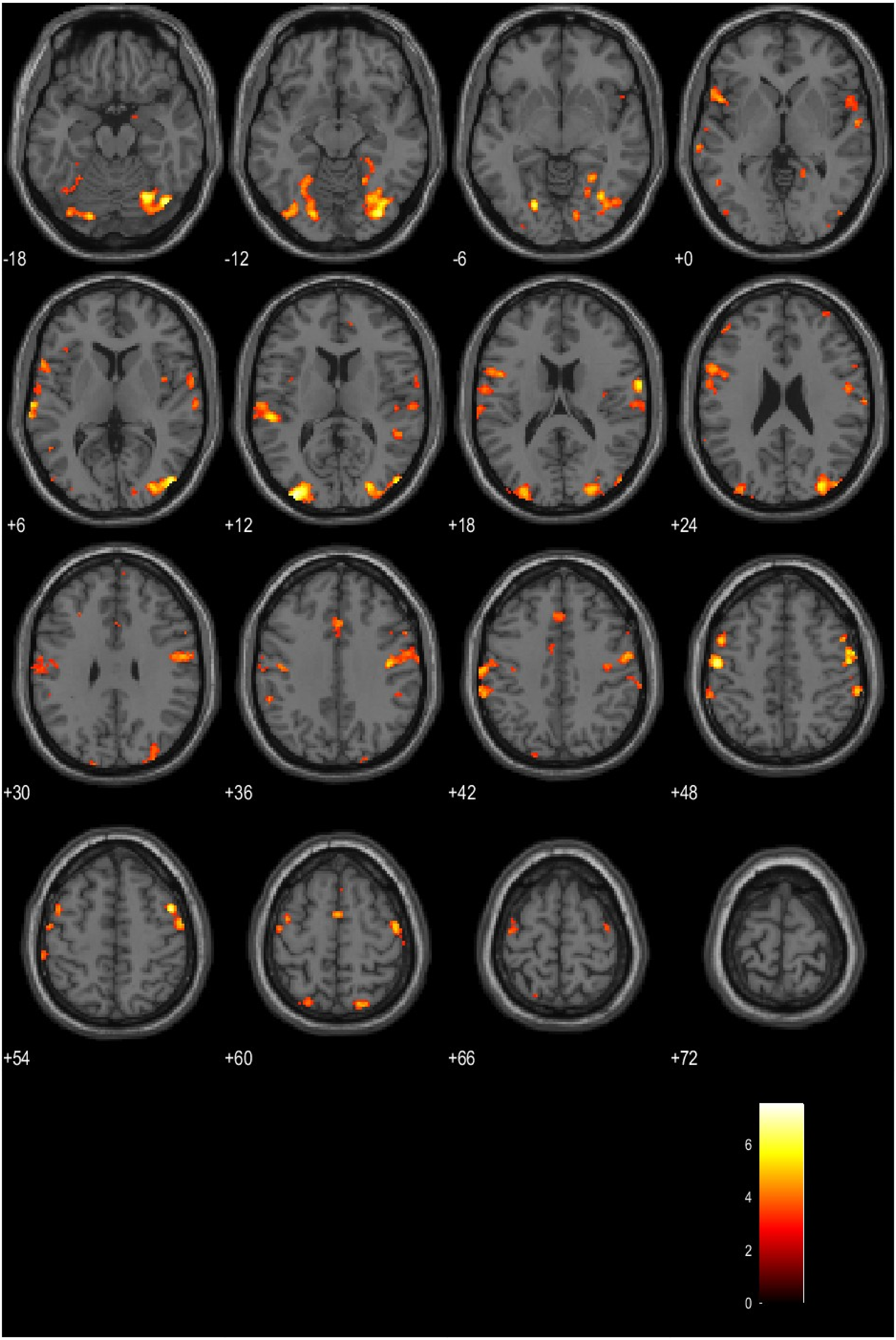

Combined Language Network for Subject 8

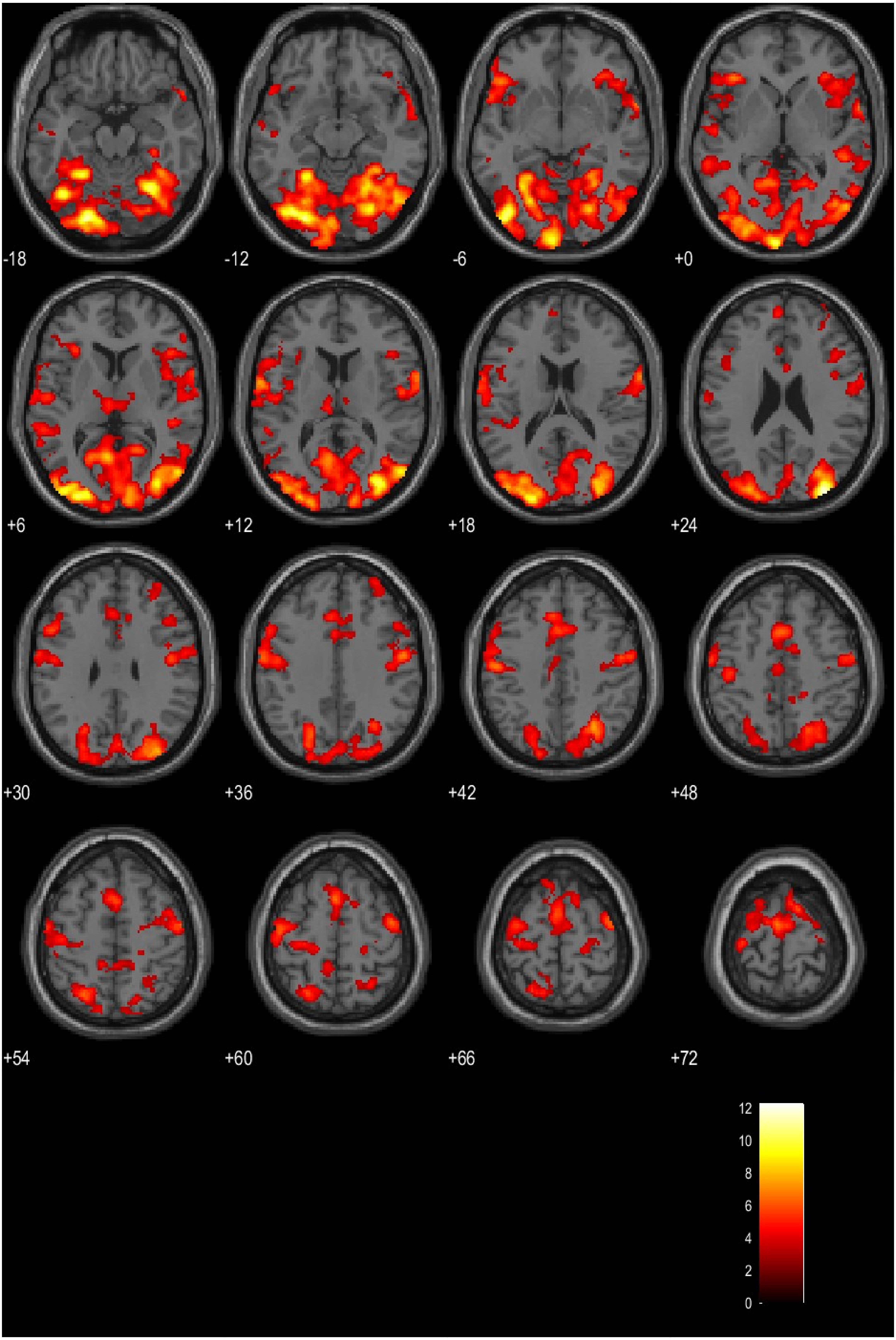

Combined Language Network for Subject 9

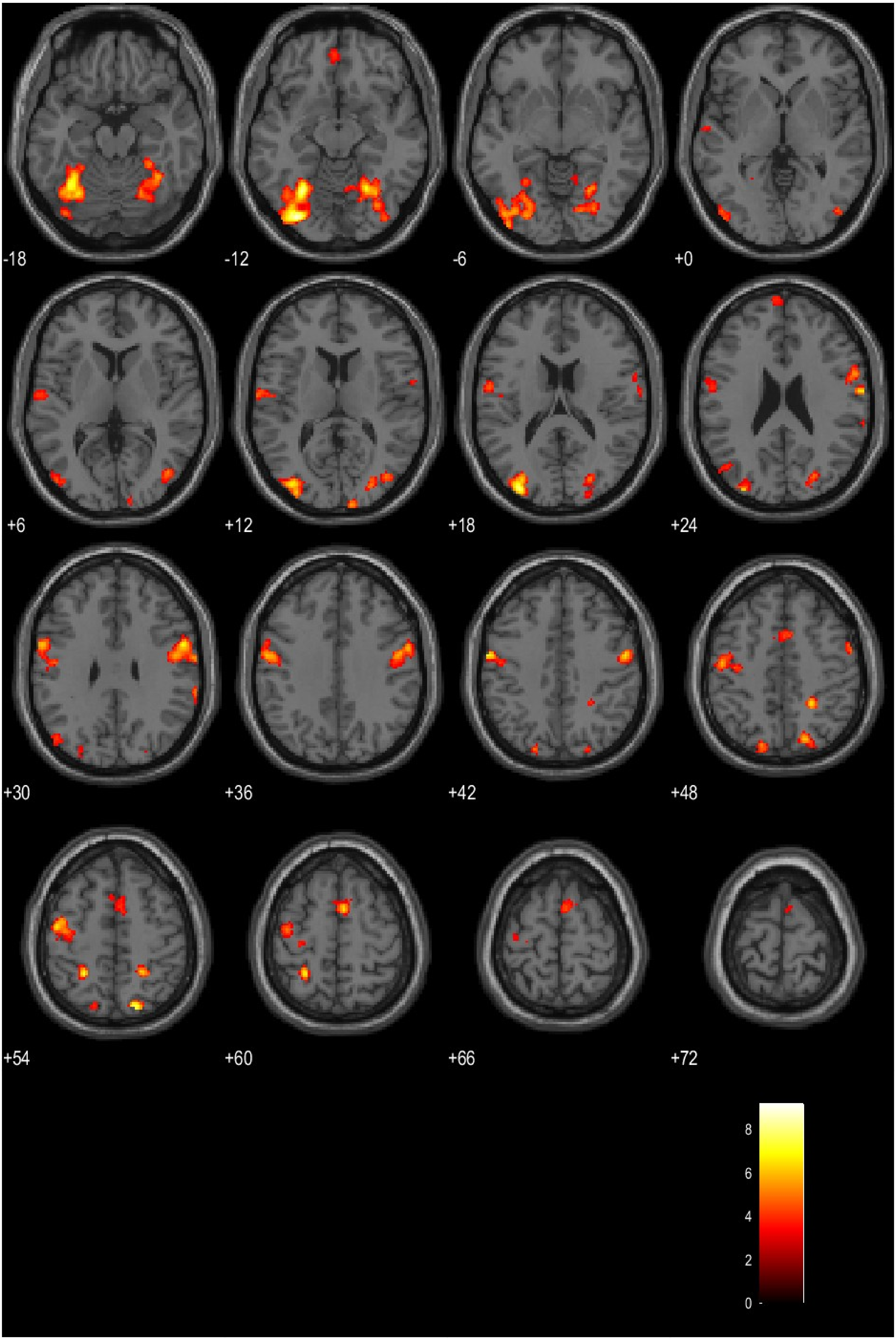

Combined Language Network for Subject 10

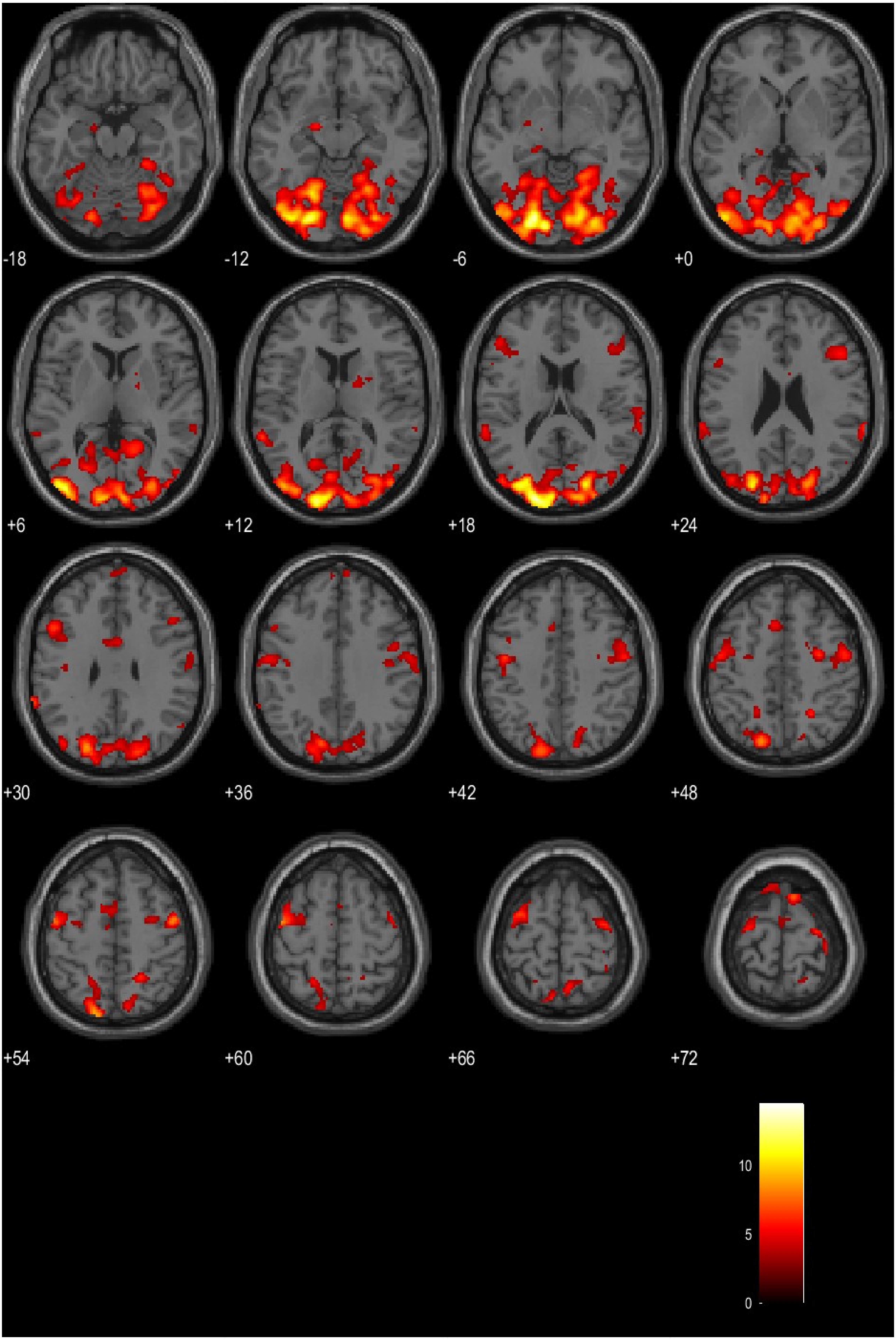

Combined Language Network for Subject 11

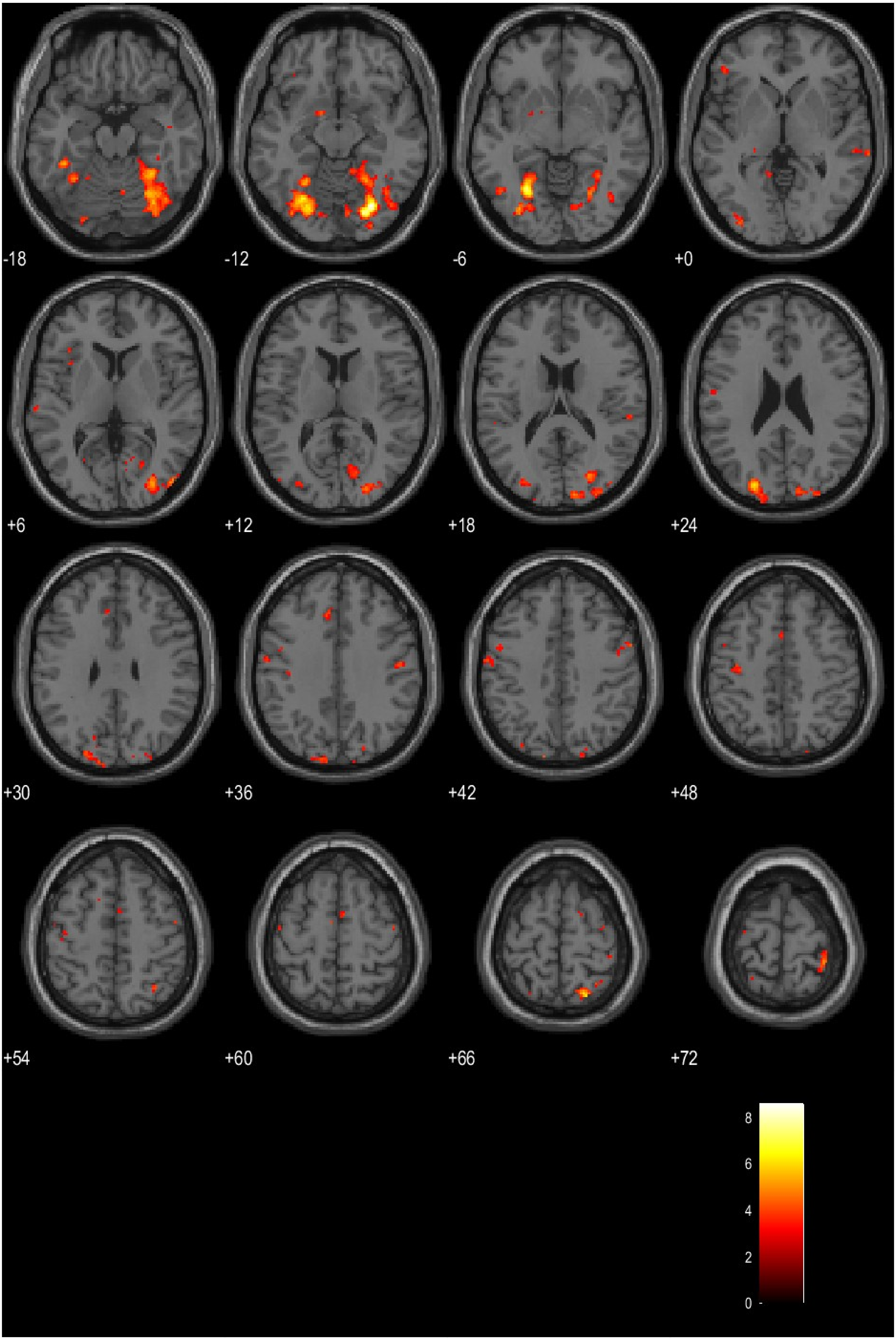

Combined Language Network for Subject 12

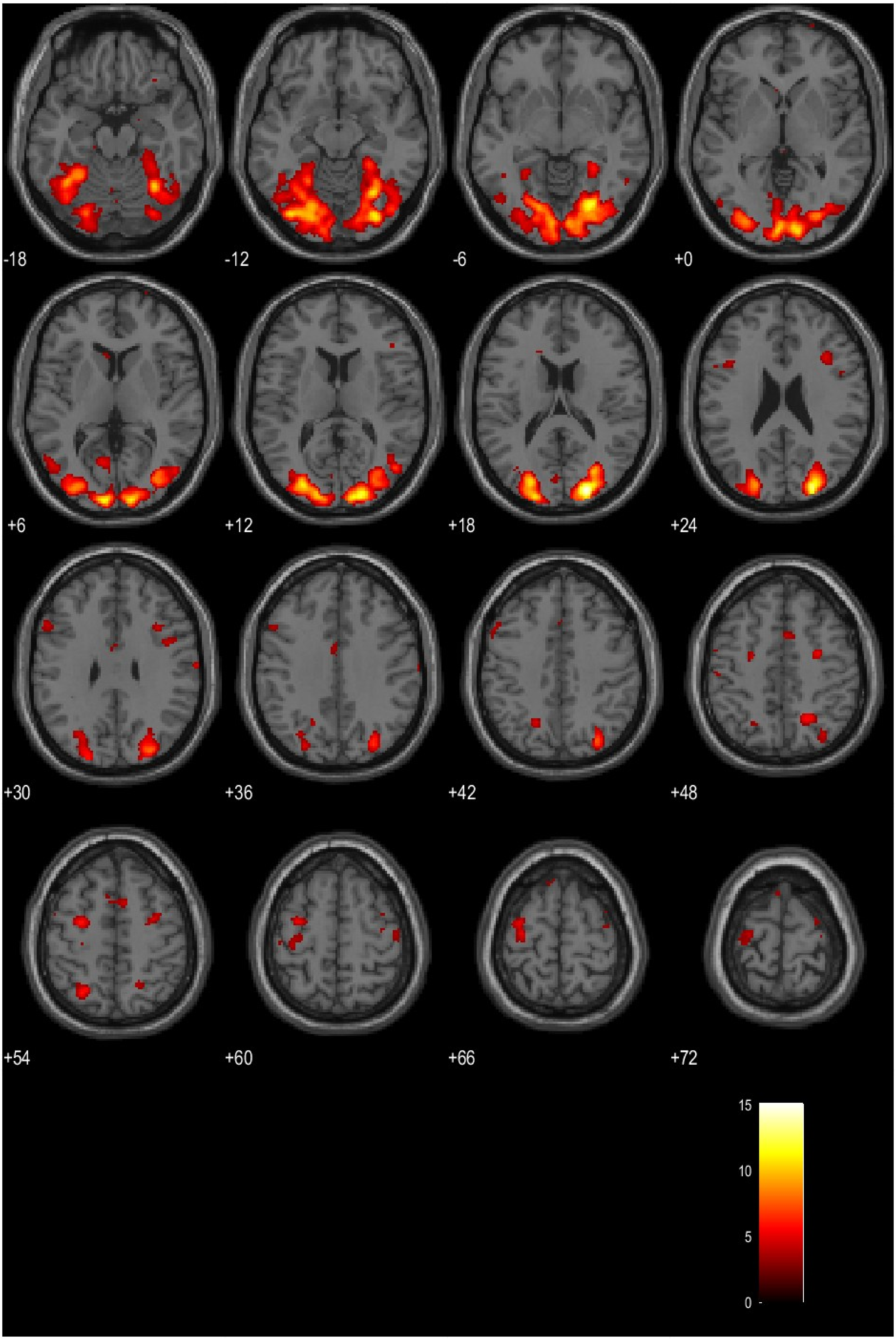

Combined Language Network for Subject 13

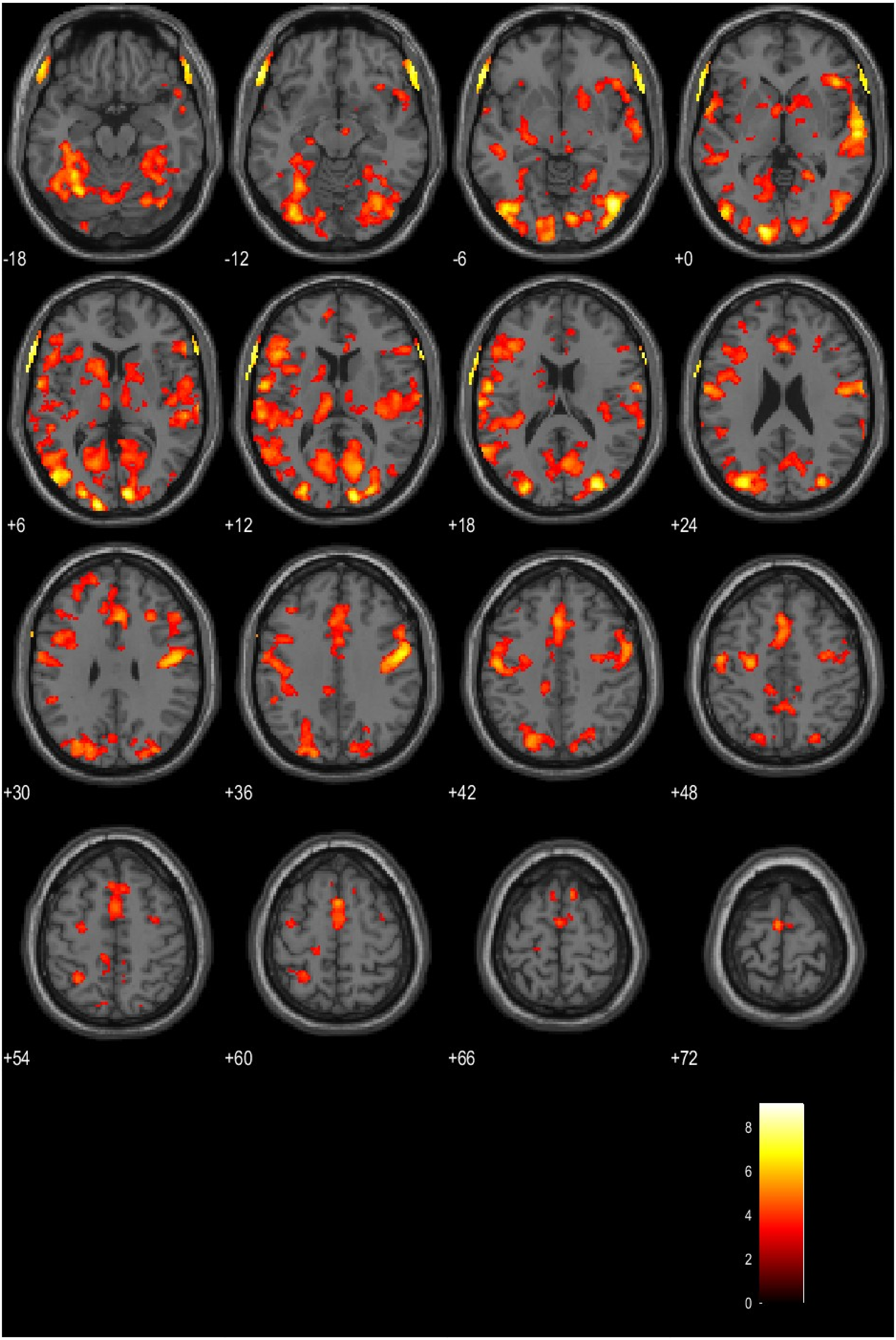

Combined Language Network for Subject 14

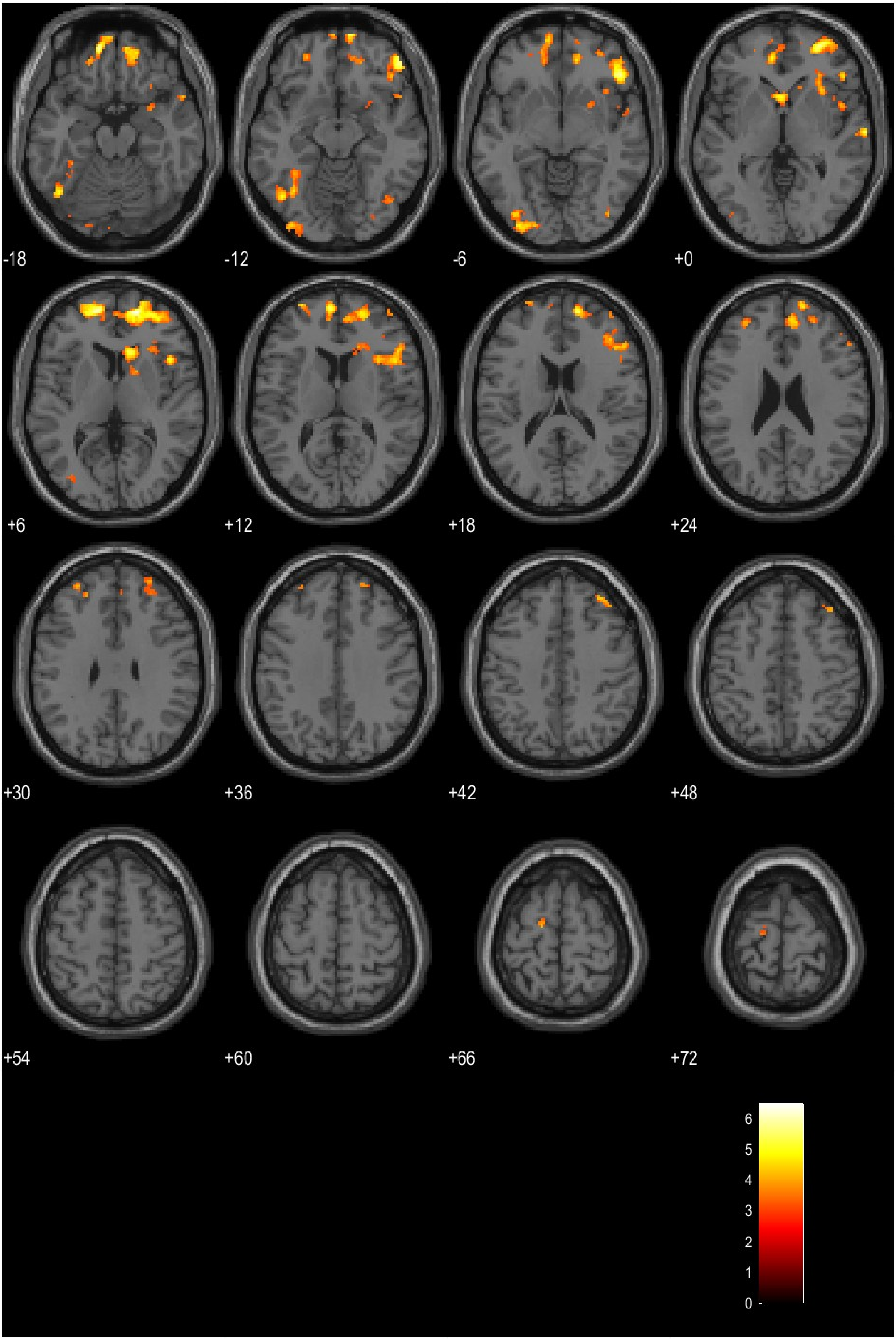

Combined Language Network for Subject 15

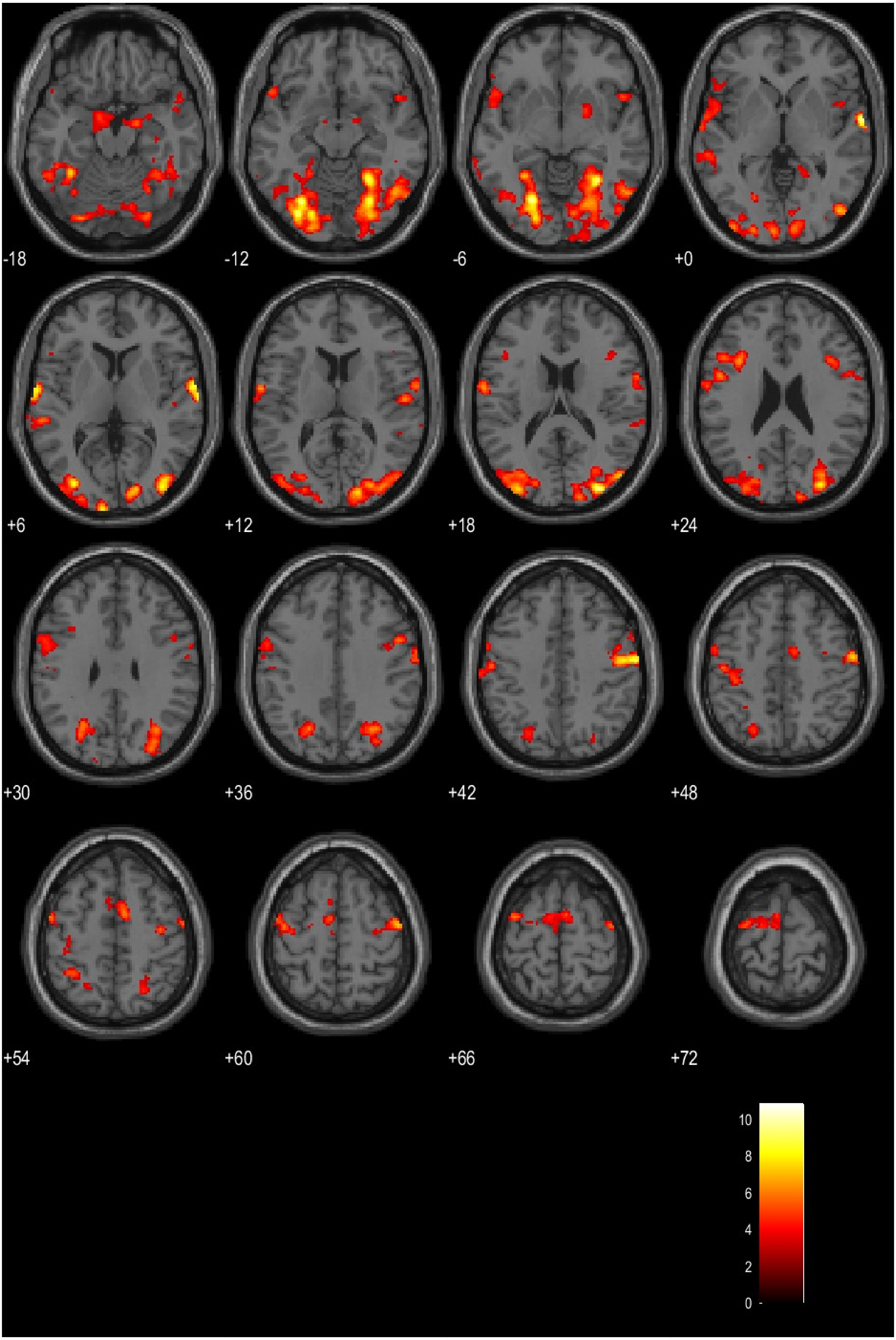

Supplement: Supplementary file 4 — Data S4:Combined language network for each subject. [file EJN-63-0-s002.pdf]
