# Supplementary material for: A Rapid fMRI Paradigm for Localisation of the Language Network
Source: Eur J Neurosci. 2026 Mar 6;63(5):e70448. doi: 10.1111/ejn.70448 (PMC12964186; doi:10.1111/ejn.70448)
Supplement: Supplementary file 5 — Data S5: Activations for each task in known language nodes. Contrast estimates have been shown in these language nodes. [file EJN-63-0-s005.pdf]

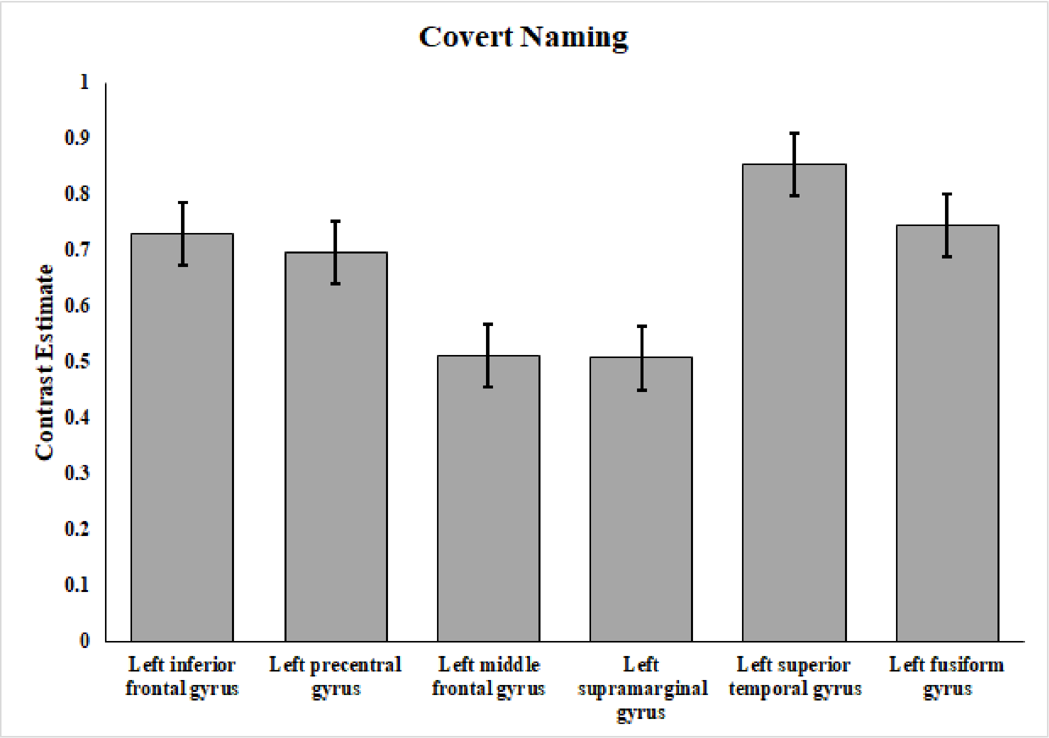
Covert Naming
Contrast Estimate
0
0.1
0.2
0.3
0.4
0.5
0.6
0.7
0.8
0.9
1
Left inferior frontal gyrus
Left precentral gyrus
Left middle frontal gyrus
Left supramarginal gyrus
Left superior temporal gyrus
Left fusiform gyrus

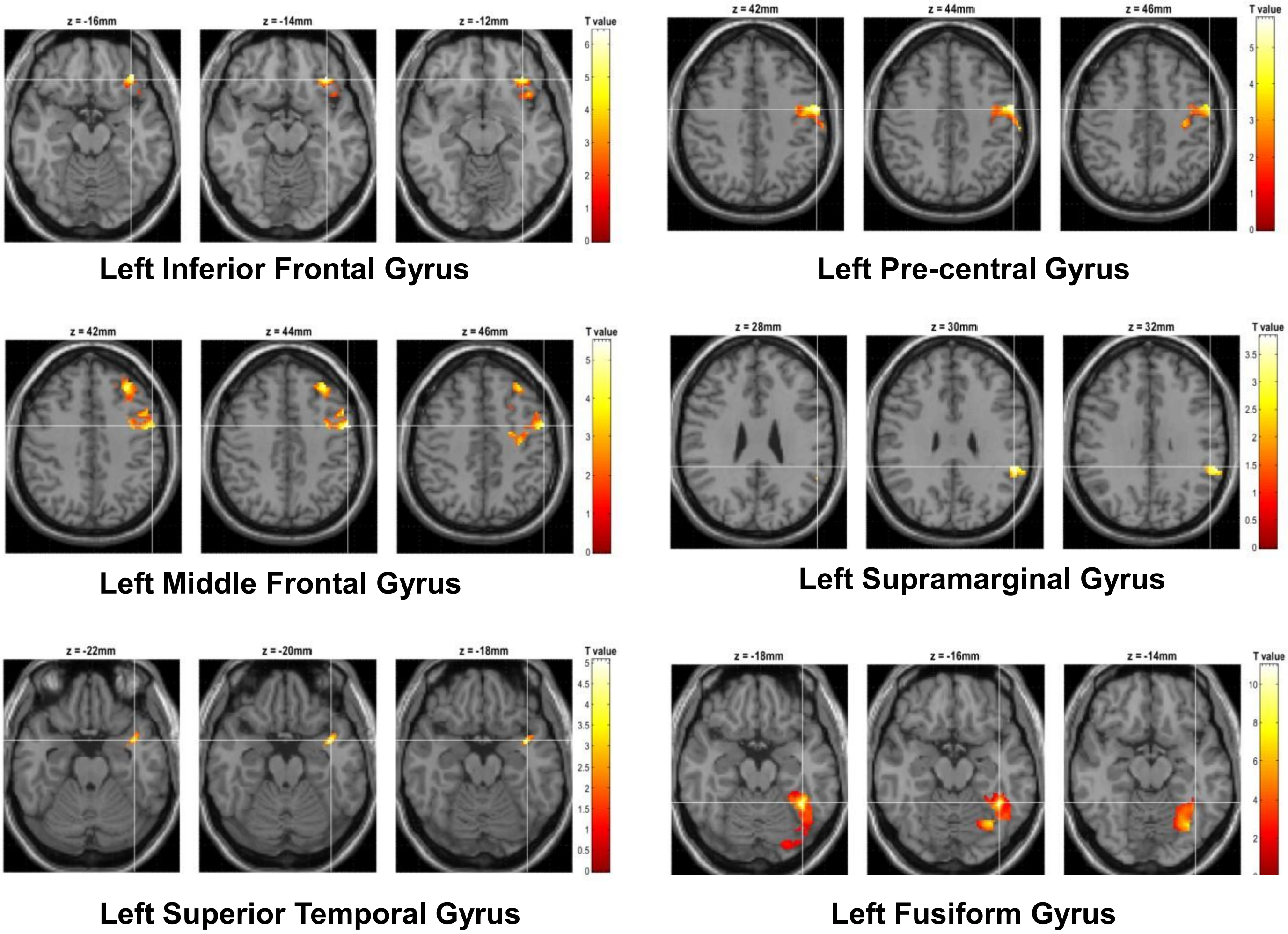
z = -16mm
z = -14mm
z = -12mm
T value
Left Inferior Frontal Gyrus
z = 42mm
z = 44mm
z = 46mm
T value
Left Pre-central Gyrus
z = 42mm
z = 44mm
z = 46mm
T value
Left Middle Frontal Gyrus
z = 28mm
z = 30mm
z = 32mm
T value
Left Supramarginal Gyrus
z = -22mm
z = -20mm
z = -18mm
T value
Left Superior Temporal Gyrus
z = -18mm
z = -16mm
z = -14mm
T value
Left Fusiform Gyrus

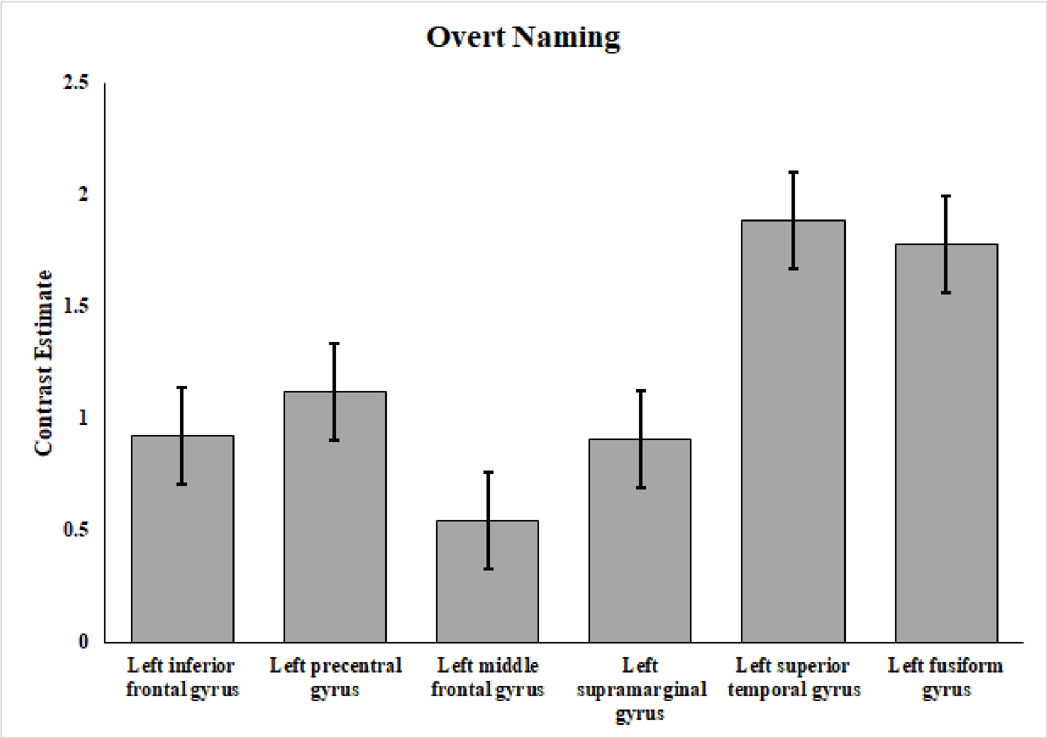

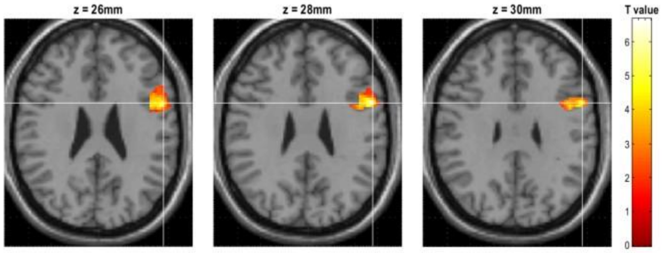

Left Inferior Frontal Gyrus

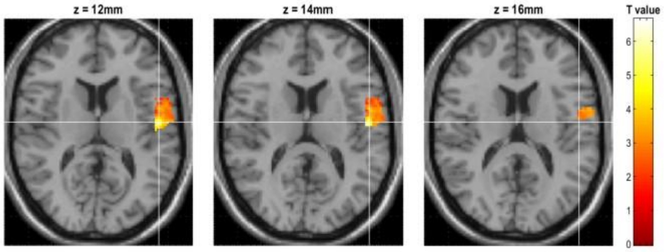

Left Pre-central Gyrus

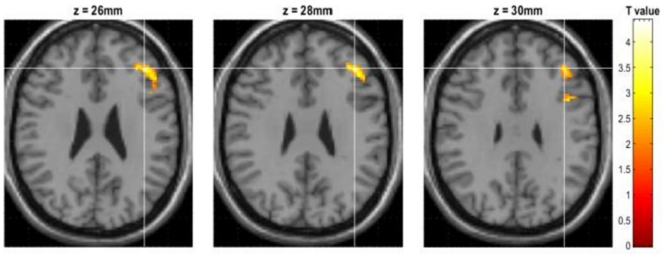

Left Middle Frontal Gyrus

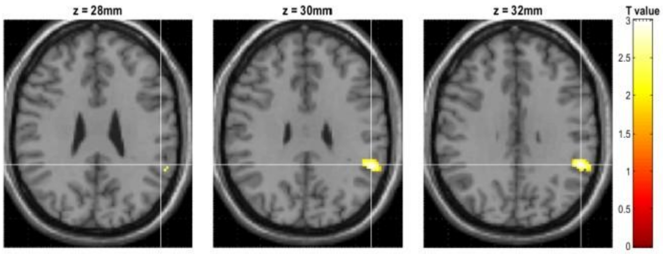

Left Supramarginal Gyrus

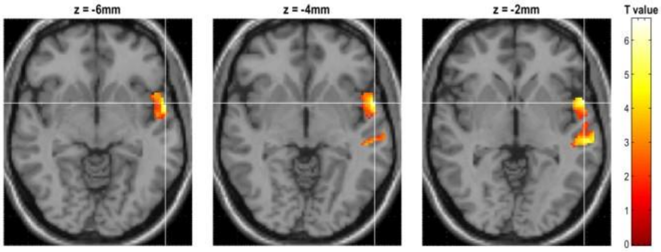

Left Superior Temporal Gyrus

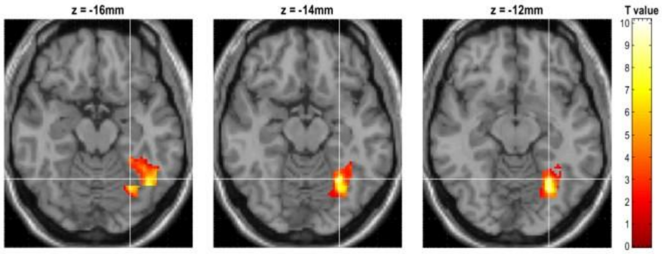

Left Fusiform Gyrus

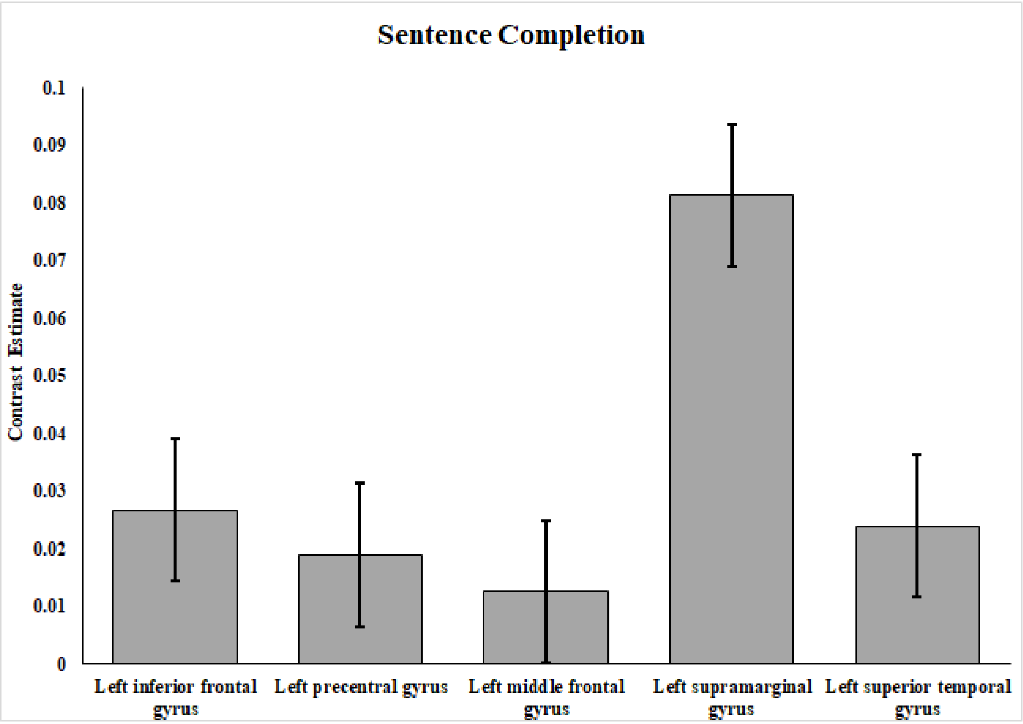

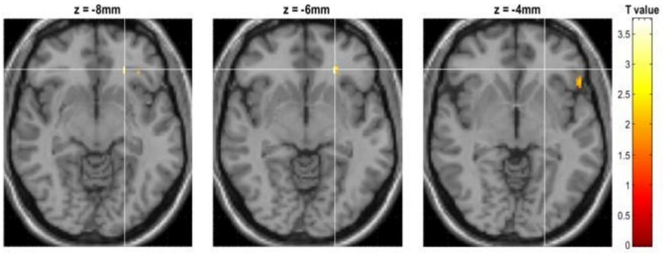

Left Inferior Frontal Gyrus

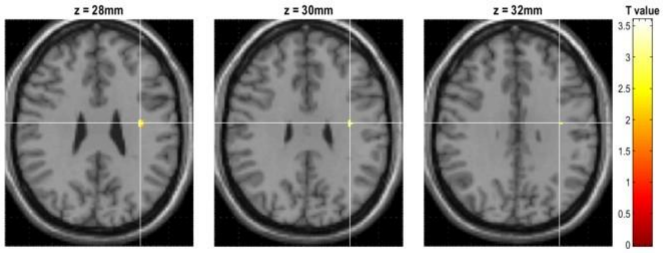

Left Pre-central Gyrus

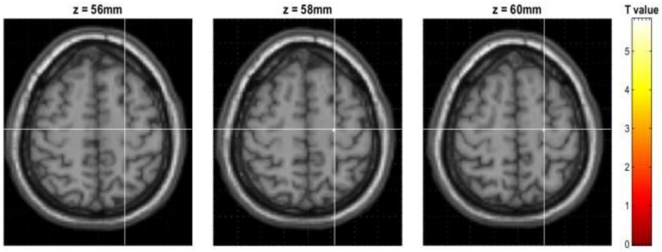

Left Middle Frontal Gyrus

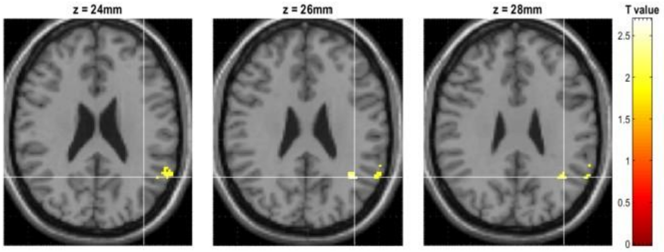

Left Supramarginal Gyrus

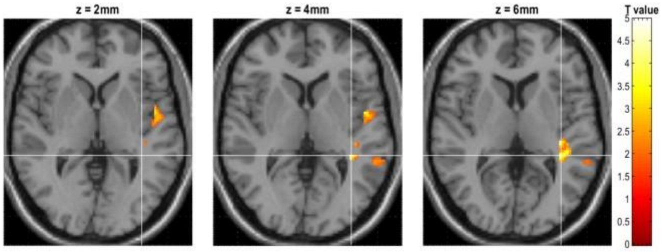

Left Superior Temporal Gyrus

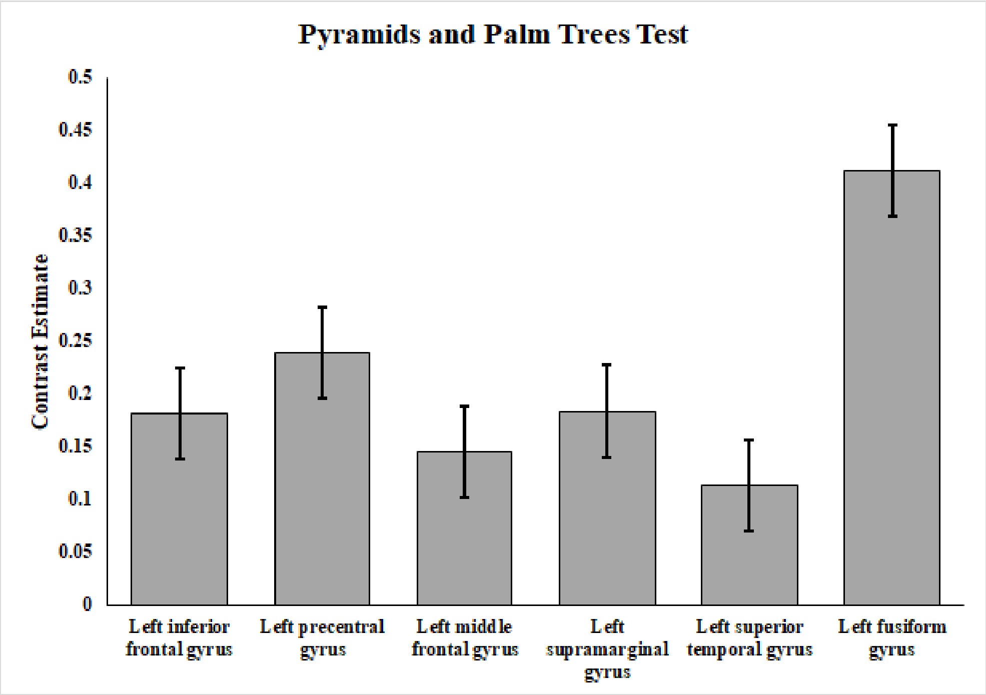

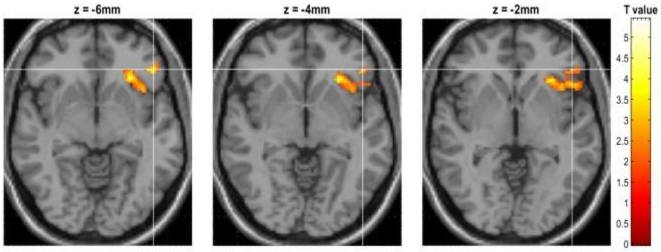

Left Inferior Frontal Gyrus

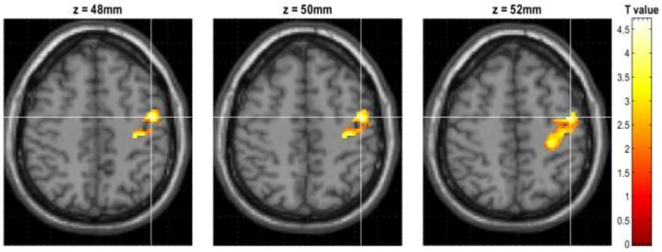

Left Pre-central Gyrus

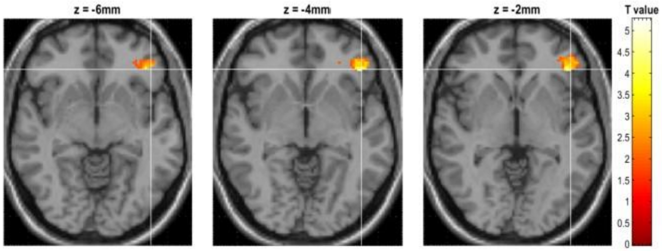

Left Middle Frontal Gyrus

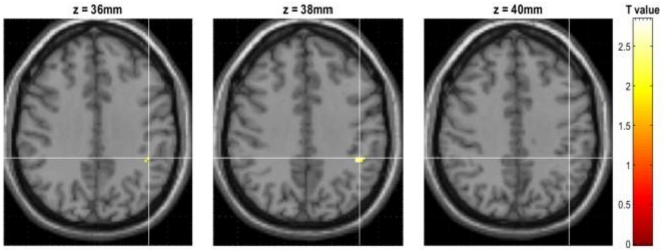

Left Supramarginal Gyrus

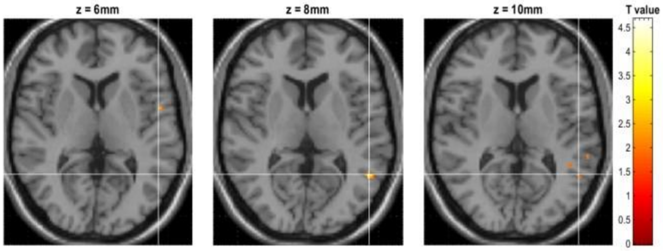

Left Superior Temporal Gyrus

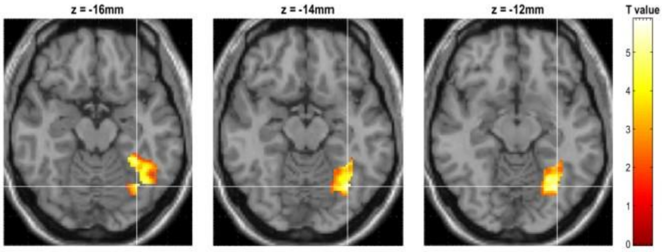

Left Fusiform Gyrus
